# Electromagnetic Modulation of the ALS Transcriptome: Toward a Non-Drug Therapeutic Strategy

**DOI:** 10.3390/cells15212003

**Published:** 2026-11-03

**Authors:** Arun Pal, Banaja P. Dash, Marc Uhlarz, Jonas Gronemann, Julia Japtok, Hannes Glaß, Jens Pietzsch, Joachim Wosnitza, Katrin Sameith, Andreas Dahl, Andreas Hermann, Richard H. W. Funk, Thomas Herrmannsdörfer

**Affiliations:** 1 Dresden High Magnetic Field Laboratory (HLD), Helmholtz-Zentrum Dresden-Rossendorf, 01328 Dresden, Germany; 2 Division for Neurodegenerative Disease, Department Neurology, Technische Universität Dresden, 01307 Dresden, Germany; 3 Translational Neurodegeneration Section “Albrecht-Kossel”, Department of Neurology, University Medical Center Rostock, University of Rostock, 18147 Rostock, Germany; 4 Department of Radiopharmaceutical and Chemical Biology, Institute of Radiopharmaceutical Cancer Research, Helmholtz-Zentrum Dresden-Rossendorf, 01328 Dresden, Germany; 5 Faculty of Chemistry and Food Chemistry, School of Science, Technische Universität Dresden, 01069 Dresden, Germany; 6 Institute of Solid State and Materials Physics, Technische Universität Dresden, 01307 Dresden, Germany; 7 DRESDEN-concept Genome Center (DcGC), Center for Molecular and Cellular Bioengineering (CMCB) Technology Platform, TUD Dresden University of Technology, 01062 Dresden, Germany; 8 Max Planck Institute of Molecular Cell Biology and Genetics, 01307 Dresden, Germany; 9 German Center for Neurodegenerative Diseases (DZNE) Rostock/Greifswald, 18147 Rostock, Germany; 10 Center for Transdisciplinary Neurosciences Rostock (CTNR), University Medical Center Rostock, University of Rostock, 18147 Rostock, Germany; 11 Institute of Anatomy, Medizinisch Theoretisches Zentrum, Technische Universität Dresden, 01307 Dresden, Germany; 12 Dresden International University, 01067 Dresden, Germany

**Keywords:** neurodegenerative disease, Lou Gehrig’s disease, magnetic-field-mediated neuroprotection, inducible pluripotent stem cells, motor neurons, axonal organelle motility, DNA damage response, global RNA-seq, extracellular matrix

## Abstract

**Highlights:**

**What are the main findings?**

- Systematic parameter optimization of electromagnetic alternating field stimulation in human neurons using quantitative disease-relevant functional readouts.
- Evidence for orientation-dependent efficacy of electromagnetic stimulation in compartmentalized axonal culture systems.

**What are the implications of the main findings?**

- Persistent and specific transcriptomic reprofiling following electromagnetic exposure.
- Electromagnetic stimulation restores both axonal organelle motility and genome maintenance in motor neurons of amyotrophic lateral sclerosis, thereby pointing to a therapeutic potential of this biophysical method.

**Abstract:**

Amyotrophic lateral sclerosis (ALS) is a fatal neurodegenerative disease hallmarked by progressive loss of neuromuscular junctions, cortical and spinal motor neurons (MNs), resulting in muscle paralysis. MNs are particularly vulnerable due to their long axons and high metabolic demands. ALS pathology involves impaired RNA metabolism, protein aggregation, cytoskeletal defects affecting organelle trafficking, and mitochondrial dysfunction, ultimately leading to neurodegeneration. Current treatments only marginally extend survival, calling for alternative therapies. We recently reported beneficial effects of electromagnetic alternating field stimulation (ACMS) on axonal regeneration and organelle motility in ALS. Here, we used iPSC-derived spinal MNs from familial ALS patients with FUS mutations to optimize magnetic field parameters regarding frequency, amplitude and duration. Using compartmentalized neuron cultures, we also tested different magnetic field orientations relative to axons. Following ACMS, we assessed axonal organelle trafficking and the nuclear DNA damage response (DDR) by live-cell imaging. Untreated ALS neurons showed clear defects in both assays. Exposure to magnetic AC sine waves above a threshold amplitude of 7 mT, applied perpendicular to the axonal plane with an optimal low frequency of 25 Hz, led to a sustained boost of mitochondrial and lysosomal motility for over one week and rehabilitation of DDR. These phenotypic improvements correlated with global transcriptomic changes revealed by RNA-sequencing. These phenotypic improvements correlated with global transcriptomic changes revealed by RNA-sequencing. While untreated mutant and wild-type neurons displayed different gene expression profiles, ACMS converged them to a novel, harmonized transcriptional state across all lines. Further analysis of differentially expressed genes (DEGs) yielded a conceptual model in which ACMS activates extracellular matrix (ECM)-mediated cell adhesion and neurotransmitter receptor, ion channel and kinase signaling pathways, ultimately modulating gene transcription and, thereby, motor- and microtubule-associated proteins. This model provides a framework for follow-up studies to experimentally validate the underlying mechanisms and assess the therapeutic potential of ACMS as an alternative biophysical treatment modality for neurodegenerative diseases.

## 1. Introduction

Amyotrophic lateral sclerosis (ALS) is a yet incurable disease affecting mainly motor neurons (MNs), leading to death, typically within 2–5 years after its onset. Progressive loss of spinal and cortical MNs results in muscle paralysis, atrophy, spasticity and ultimately respiratory failure [1]. Approximately 10% of cases are familial (fALS) with mostly autosomal dominant inherited genetic mutations, while the majority (~90%) occur sporadically (sALS) with unknown causes [2]. To date, more than 30 genes have been identified in fALS, most frequently affected being superoxide dismutase 1 (*SOD1*), TAR DNA-binding protein 43 (*TDP-43*), fused in sarcoma (*FUS*), and the GGGGCC hexanucleotide repeat expansion in the *C9ORF72* gene, which alone accounts for 40–50% of fALS cases [2]. Disease onset usually occurs between 60 and 70 years of age, whereas mutations in *FUS* or senataxin (*SETX*) often lead to much earlier onset in the late 20 s to 30 s [3,4,5,6,7]. The worldwide prevalence is approximately 4.5 per 100,000 individuals, with peak incidence around 70 years of age [8].

Despite extensive research, only a few FDA-approved pharmacological treatments are available, mainly Riluzole and Edaravone, all of which extend patient survival by only a few months [9,10,11,12,13]. Hallmarks of ALS pathology include toxic RNA accumulation, impaired protein quality control, cytoskeletal instability, disrupted axonal organelle trafficking, mitochondrial dysfunction [14], and defective DNA damage response (DDR) pathways [15]. In ALS-FUS, mutations occur across several functional domains, most commonly within the carboxyterminal nuclear localization signal (NLS). These mutations promote cytoplasmic mislocalization, aggregation, stress granule formation, oxidative stress, and disturbances in RNA and protein homeostasis [16]. Previous work by us and others demonstrated that spinal MNs derived from induced pluripotent stem cells (iPSCs) of fALS patients carrying FUS-NLS mutations show severely reduced mitochondrial and lysosomal axonal transport, impaired regeneration after axotomy, and pronounced DDR defects with accumulation of DNA damage [15,17,18].

The dramatic lack of effective therapies for ALS and other neurodegenerative diseases has motivated the exploration of non-pharmacological treatment strategies, including electromagnetic field stimulation (EMS). Time-varying magnetic fields can influence biological systems by exerting dynamic forces on magnetic ions and free radicals, inducing electrical currents in conductive tissues, affecting complex anisotropic diamagnetic macromolecules such as microtubules, membranes, and DNA, and modulating radical pair spin chemistry [19,20,21]. These effects can alter ion channel gating, ion pump activity, and signaling pathways, including Ca^2+^ influx and downstream transcriptional regulation [22,23,24]. Cryptochromes have been proposed as potential magneto-receptors capable of triggering G-protein–coupled signaling cascades in response to magnetic fields [20,25].

Across numerous experimental settings, two major EMS modalities have been established: pulsed electromagnetic fields (PEMFs) and alternating current magnetic stimulation (ACMS). PEMFs consist of trains of short pulses characterized by pulse duration and repetition rate. In contrast, ACMS generates continuous oscillating magnetic fields defined by waveform and frequency. Although both use Hertz as a unit, their technical characteristics are distinct and should not be conflated (Figure S1). Most studies to date have focused on PEMF exposure. Beneficial effects of PEMFs have been reported in bone and cartilage regeneration, osteoporosis, osteoarthritis, inflammation, wound healing, and trauma treatment [23,26,27,28,29]. Correctly tuned PEMFs can promote osteogenic, chondrogenic, and neurogenic differentiation by activating lineage-specific gene programs [30].

In neurology, magnetic stimulation has been established as a non-invasive diagnostic and therapeutic tool in the clinic for over two decades, most notably transcranial magnetic stimulation (TMS), also in ALS [31,32,33]. ACMS has also been shown to promote neuronal differentiation in vitro via calcium channel upregulation [34]. However, applications targeting peripheral nerves and MN diseases remain limited. PEMF treatment has been reported to enhance muscle and nerve regeneration after traumatic injury in animal models [35], and a clinical pilot study demonstrated improved muscle strength in ALS patients following PEMF stimulation, likely due to enhanced neuromuscular reinnervation [36].

Given the diversity of EMS parameters [37] and inconsistent reporting across studies, we aimed to systematically optimize ACMS parameters for therapeutic efficacy in ALS-relevant phenotypes. We previously showed that continuous 10 Hz square-wave ACMS more effectively rescued axonal trafficking defects and regeneration capacity in FUS-NLS mutant MNs than 2 Hz stimulation, indicating a frequency-dependent effect [18]. To identify the optimal frequency, we conducted a systematic screen using single-frequency sine waves across a range of 0–200 Hz. Since our previous work demonstrated sustained phenotypic rescue after cessation of ACMS [18], we further investigated the persistence of beneficial effects in chase experiments. Conversely, we assessed potential ad hoc effects by applying ACMS during live imaging to test for feasible direct interactions with cytoskeletal components or motor proteins. Additionally, we determined the minimum effective field strength required for phenotypic rescue, an important consideration for future clinical or domestic applications.

Finally, to elucidate the molecular mechanisms underlying ACMS-mediated sustained rescue, we performed whole transcriptome analysis using next-generation RNA-sequencing (RNA-seq). RNA-seq-based differential gene expression analysis of iPSC-derived spinal MNs from ALS-FUS patients was instrumental in identifying key genes and molecular pathways driving ALS pathogenesis [38,39]. These studies highlighted that mutant FUS causes severe transcriptional dysregulation, particularly involving the cytoplasmic mislocalization of FUS, which drives neurodegeneration, DNA damage, and synaptic dysfunction [40,41].

In summary, we successfully optimized ACMS parameters to robustly improve impaired axonal mitochondrial and lysosomal trafficking as well as DDR defects in ALS-FUS spinal MNs in vitro. The accompanying transcriptomic analysis provides insights into the cellular response to ACMS and identifies molecular pathways potentially responsible for the observed phenotypic improvements.

## 2. Materials and Methods

### 2.1. Ethics Statement and Patient Characteristics

Spinal motor neuron (MN) cultures were generated from induced pluripotent stem cells (iPSCs) obtained from patients with fALS carrying the pathogenic FUS variants R521C^het^, R521L^het^, or R495QfsX527^het^. These cultures were compared with MNs derived from three independent healthy donor cell lines carrying the corresponding human wild-type alleles (WT; Ctrl 1–3; Table 1). In addition, the parental FUS R521C line was used to establish an isogenic pair consisting of FUS-GFP P525L MNs and a FUS-GFP WT control by CRISPR-Cas9 gene editing, as previously described [15].

The original patient- and control-derived cell lines were established from skin biopsy material and have been characterized in previous studies [15,42,43] (Table 1). All procedures involving human material were conducted in accordance with the Declaration of Helsinki (WMA, 1964) and were approved by the Ethics Committee of the Technische Universität Dresden, Germany (EK 393122012, approved 11 November 2013, and EK 45022009, approved 24 March 2009). Written informed consent for the use of the samples and publication of the resulting research data was obtained from all participants.

### 2.2. Genotyping

Genomic DNA obtained from all cell lines was analyzed using the diagnostic human genetics laboratory CEGAT (Tübingen, Germany) to confirm their respective genotypes. Corresponding analyses of the control cell lines revealed no mutations associated with ALS.

### 2.3. Mycoplasma Testing

All cell lines were tested for mycoplasma contamination upon receipt in our laboratory and again following reprogramming. Thereafter, routine mycoplasma screening was performed at intervals of three to six months. Mycoplasma testing was carried out using the Venor^®^GeM Mycoplasma Detection Kit (Minerva Biolabs GmbH, Berlin, Germany, cat. no. 11-1025) according to the manufacturer’s instructions.

### 2.4. Generation, Gene-Editing MN Differentiation

**Figure 1:**
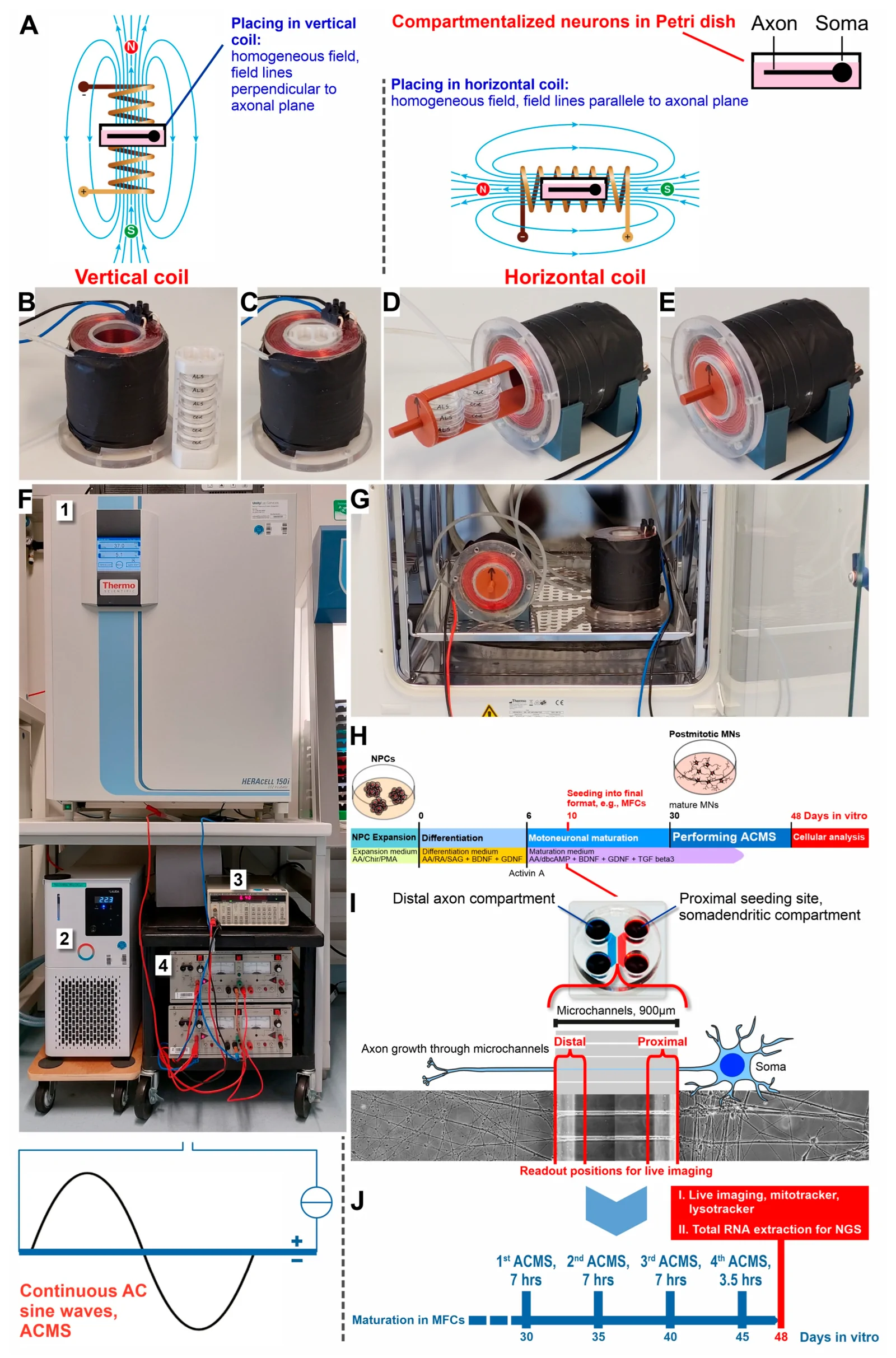
Experimental setup for ACMS of iPSC-derived human spinal MNs. (**A**) Cartoon of compartmentalized neurons cultured in MFC in Petri dish (**top right**) and placed inside the central bore of a solenoid in either vertical (**left**) or horizontal orientation (**right**). Magnetic field lines (in blue) are perpendicular with respect to the axonal plane (**left**) or in parallel (**right**). Note that the illustrated field polarities (north-south, N-S) only present schematic snapshots, as the solenoids are powered by ACs. (**B**) Vertical solenoid with electric wires and tubes for water cooling with its custom-tailored sample holder for 6 stapled glass-bottomed Petri dishes (3.5 cm Ø), each harboring one MFC. (**C**) Ditto with the sample holder placed inside the solenoid’s central bore. (**D**) Horizontal solenoid with similar wires and tubes with its custom-tailored sample holder for 6 Petri dishes. (**E**) Ditto with the sample holder placed inside the solenoid’s central bore. (**F**) Cell culture incubator (37 °C, 5% CO_2_, humid air) (1) harboring the solenoids, equipped with a thermostatic water pump (2) underneath, a function generator (3) to create AC sine waves at the desired frequency (Hz), and electrical amplifiers (4) to boost the AC amplitude up to the desired magnetic field strength (mT). (**G**) Incubator with opened front door and solenoids in horizontal (**left**) and vertical (**right**) configuration placed inside. Note that the wires for electrical support run across the seal of the front door, whereas the thicker tubes for the water cooling enter through a plug in the rear wall (outside the image). The interior is made of non-magnetic stainless steel to minimize any interactions and distortions of the AC magnetic field as well as the induction of eddy currents. (**H**) Time line cartoon illustrating the in vitro differentiation of human iPSC-derived spinal MNs starting with the expansion of NPCs in ascorbic acid (AA), chiron (Chir) and purmorphamin (PMA), the commencement of neurite differentiation at 0 days in vitro (DIV) in AA, retinoic acid (RA), smoothened agonist (hedgehog pathway activator, activates smoothened) (SAG), brain-derived neurotrophic factor (BDNF) and glial-cell-derived neurotrophic factor (GDNF), via the maturation in AA, dibutyryl-cyclo-adenosine monophosphate (dbcAMP), BDNF, GDNF and tissue growth factor beta3 (TGF beta3) from 6 DIV onwards to the final seeding into MFCs at 10 DIV to obtain compartmentalized cultures for ACMSs performed from 30 DIV onwards and final readout assays hereafter at 48 DIV. (**I**) Illustration of compartmentalized neuron cultures in Xona MFCs with the proximal seeding site on the right to obtain a somadentritic compartment in the channel connecting the upper with the lower well (stained in red). The somadenritic compartment is physically separated from the distal axon compartment on the left (stained in blue) by a groove of 900 µm microchannels which can only be penetrated by axons eventually sprouting out into the distal compartment beyond the microchannels’ exit on the left. Readout positions for live imaging of distal versus proximal axonal organelle motility at 48 DIV are highlighted in red. (**J**) Timeline cartoon of the four consecutive ACMS sessions from 30–45 DIV and the readout assays performed at 48 DIV to score for sustained, beneficial effects lasting beyond the ACMS sessions, namely on axonal organelle motility by live imaging (mitotracker, lysotracker) and on changed global gene expression profiles (transcriptomics, RNA-seq).

The derivation, comprehensive characterization, and expansion of the iPSC lines used in this study have been reported previously [15,42,43]. These included lines from healthy control donors and patients with fALS carrying defined NLS mutations in FUS, as well as the isogenic pair comprising the carboxy-terminally tagged mutant FUS-GFP P525L line and its gene-corrected FUS-GFP WT counterpart (Table 1). The procedures used to differentiate all iPSC lines into neuronal progenitor cells (NPCs) and subsequently mature them into spinal MNs have likewise been described previously [15,43]. The NPC-to-MN differentiation workflow is schematically depicted in Figure 1A of Naumann et al. (2018) [15] and in Figure 1H of the present study.

### 2.5. Growth of MNs in Microfluidic Chambers (MFCs)

**Figure 2:**
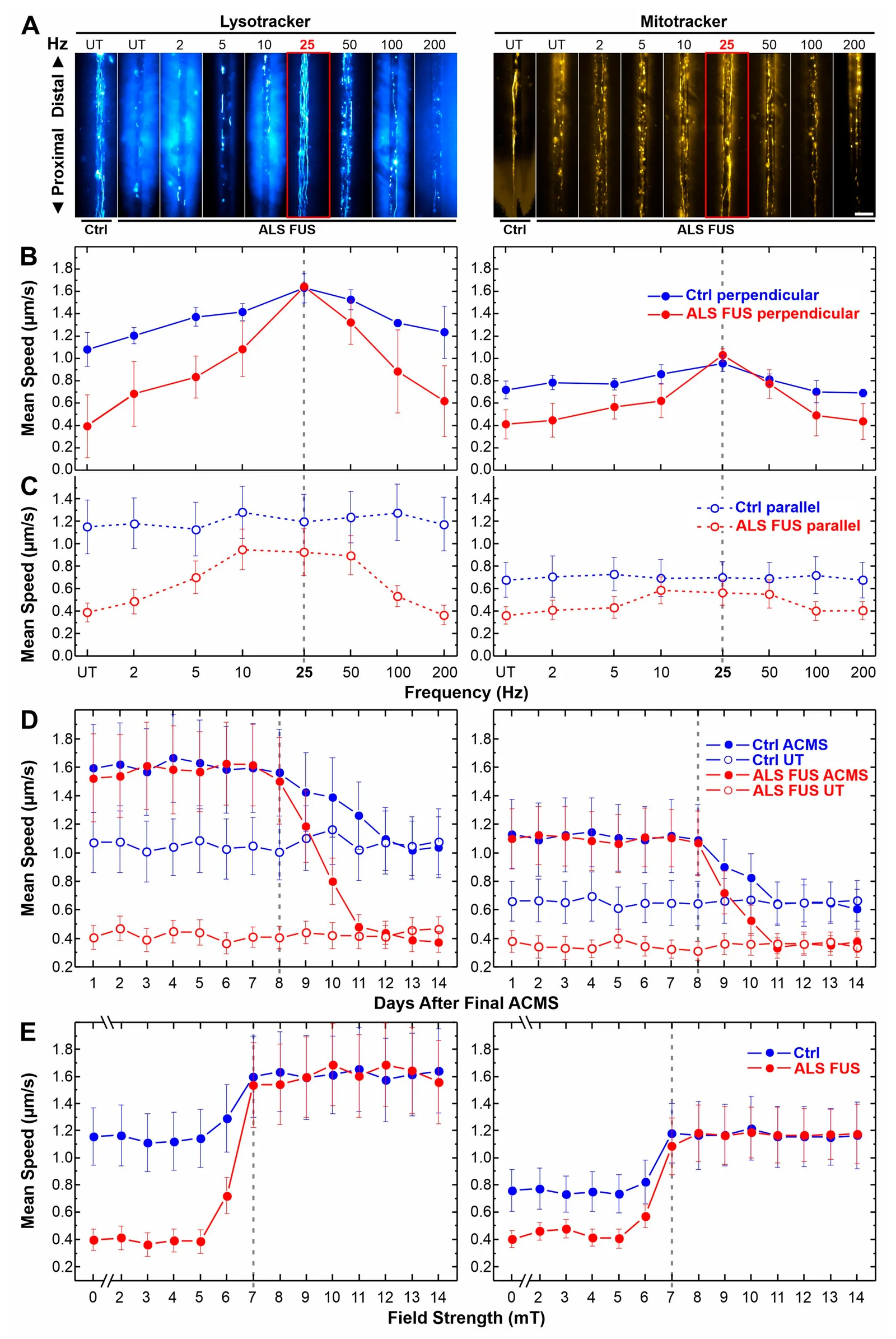
Rescue of axonal organelle motility is sustained beyond the final ACMS session and depends on frequency, orientation and strength of the magnetic field. (**A**) Maximum intensity projections of movies acquired with lysotracker (**left**, in cyan) and mitotracker (**right**, in yellow) on the isogenic pair of FUS-GFP WT MNs (Ctrl) versus the mutant FUS-GFP P525L (ALS-FUS) (Table 1) at the distal microchannel end in MFCs as shown in Figure 1I after the final ACMS session at 48 DIV, as shown in Figure 1J. All four consecutive ACMS sessions were performed as shown in Figure 1J at 10 mT of perpendicular field orientation (Figure 1A–C) and the indicated AC frequency, respectively, and compared against untreated (UT) cells. Scale bar = 10 µm; corresponds to Movie S1. Straight organelle movements appear as long trajectories, whereas immobile organelles project as punctae. Note the drastically reduced organelle motility in the untreated ALS-FUS mutant as compared to untreated Ctrl cells and the rescue through ACMS with a clear optimum at 25 Hz for either type of organelle (boxed in red). (**B**–**E**) Quantitative tracking analysis of organelle motility, e.g., as shown in panel (**A**). Individual organelles in the entire pool of examined movies were analyzed with respect to mean speed and further tracking parameters (i.e., track displacement, moving fraction, and ratio of antero-/retrograde motility; see Figures S2–S5) as a function of ACMS frequency (panel (**B**)), field orientation (panel (**C**)), chase time after the final ACMS session (panel (**D**)) or field strength (panel (**E**)) and compared against untreated cells (UT, 0 mT), respectively. Data points are the means of the entire analyzed organelle population, resulting in sample sizes in the tens of thousands across all pooled movies per condition, e.g., for a specific frequency and cell line, with error bars presenting the standard deviation. (**B**) Mean speed (*y*-axis) of either lysosomes (**left**) or mitochondria (**right**) deduced at 48 DIV (Figure 1J) and plotted over ACMS frequency (*x*-axis) in the 4 preceding ACMS sessions (Figure 1J), each performed at perpendicular orientation of the field (Figure 1A–C), 10 mT, and the indicated frequency. Note the clear optimum of ACMS at 25 Hz, leading to a rescue of organelle motility in the ALS-FUS mutant even beyond the natural (UT) Ctrl level. Moreover, Ctrl cells responded to that treatment in a similar way, i.e., they showed the same stimulated motility level. (**C**) Same as panel (**B**) except that all 4 ACMS sessions (Figure 1J) were performed at parallel field orientation (Figure 1A–C). Note that the rescue of organelle motility as compared to perpendicular field orientation (panel (**B**)) was far less efficient. (**D**) Mean speed (*y*-axis) of either lysosomes (**left**) or mitochondria (**right**) deduced over a chase from 1–14 days after the final ACMS session (Figure 1J). All 4 preceding ACMS sessions (Figure 1J) were performed at a perpendicular field orientation (Figure 1A–C), 10 mT, and the optimal frequency of 25 Hz. Note the beneficial, stimulatory effect on the motility of both types of organelles (lysosomes and mitochondria) that was fully sustained beyond the final ACMS session (Figure 1J) for at least 8 days (compare closed versus open circles). Moreover, the stimulation of organelle motility in mutant ALS-FUS (red closed versus red open circles) was even beyond the natural (UT) Ctrl level (blue open circles) and Ctrl cells responded to the ACMS treatment in a similar way, i.e., they showed the same stimulated motility level (blue closed versus blue open circles). (**E**) Mean speed (*y*-axis) of either lysosomes (**left**) or mitochondria (**right**) deduced at 48 DIV (Figure 1J) and plotted over ACMS field strength (*x*-axis) applied in the 4 preceding ACMS sessions (Figure 1J), each performed at a perpendicular orientation of the field (Figure 1A–C), the optimal frequency of 25 Hz, and the indicated field strength (mT). Note the full stimulatory effect on the motility of both types of organelles (lysosomes and mitochondria) at 7 mT and higher in both lines (Ctrl & ALS-FUS), whereas below 7 mT organelle motility remained indistinguishable from untreated cells; i.e., motility did not gradually increase over field strength but, instead, suddenly switched at the critical threshold of 7 mT from an unstimulated low to a stimulated higher level even beyond untreated Ctrl cells.

MFCs (Xona Microfluidics^®^ Inc., Durham, NC, USA, cat. no. RD900) were coated and assembled as previously described [15,46] before the seeding of MNs. MNs were introduced into one compartment of the chamber and allowed to mature, thereby establishing a compartmentalized culture system in which neuronal somata and dendrites remained spatially separated from distal axons. Only axons were able to traverse the microgroove barrier consisting of 900 µm long microchannels and subsequently emerge into the distal compartment, where they extended further into the open chamber (Figure 1I, [15]). A schematic representation of this compartmentalized MFC culture system has been provided previously in Figure 2A of Naumann et al. (2018) [15]. All imaging experiments were performed at 48 days in vitro (DIV), corresponding to 48 days of axonal growth and MN maturation, with DIV 0 defined as the onset of differentiation (Figure 1H). Images and corresponding movies shown in this study were acquired from strictly standardized regions of individual microchannels immediately adjacent to their distal exits (Figure 1I and Figure 2A). These regions typically contained axon bundles comprising approximately 5–20 individual axons. Because of the optical resolution limits and the dense three-dimensional architecture of the compartmentalized cultures, individual axons within these bundles could not be reliably distinguished or followed continuously from their somata in the proximal compartment through the microchannels to their distal growth cones. Consequently, the organelle trafficking and morphological parameters determined in the present study could not be related to the total length of individual axons or similar single-axon characteristics. This technical limitation, however, did not affect the bulk analysis of organelle dynamics or the biological questions addressed here. As demonstrated in our previous work [15], the neurites present within the microchannels and extending beyond their distal exits consisted exclusively of MN axons. No downstream target cells, such as myotubes connected to the distal growth cones, were present in these cultures; thus, no composite or co-culture system was established.

### 2.6. Alternating Current Magnetic Field Stimulation (ACMS)

Cylindrical magnetic field coils (solenoids) were designed and fabricated for operation inside standard cell culture incubators. To counteract temperature increases above 37 °C caused by ohmic losses, the coils were equipped with a water-cooling system. The cooling circuit was supplied by a pump connected to a thermostatic water bath maintained at 37 °C. Thus, active cooling of the solenoids below 37 °C was not possible, whereas excess heat generated during ACMS was efficiently dissipated. Each solenoid had an open central bore with a diameter of 50 mm, a height and total diameter of 100 mm, and accommodated up to six cell culture dishes (35 mm diameter, 12 mm height) in custom-designed sample holders. This configuration allowed simultaneous exposure of the cultures to the magnetic field within the central bore (Figure 1A–E). The temperature was continuously monitored within the central bore of the solenoid using a sensor positioned at a blank location of the sample holder (Figure 1B,C). Even after 5 h of continuous ACMS operation, the temperature remained within a narrow range of 36.5–37.5 °C, indistinguishable from temperatures measured at other locations within the incubator but outside the solenoid (Figure 1F,G). DC stimulation performed at comparable electrical power dissipation, and therefore under comparable thermal conditions, did not reproduce the effects observed at optimized ACMS conditions, thereby arguing against temperature effects as the primary cause of the observed phenotypic changes. The spatial distribution and homogeneity of the magnetic field inside the central bore were precisely modeled with the solenoid’s dimensions (see above) using COMSOL Multiphysics v6.3 (COMSOL Multiphysics GmbH, Göttingen, Germany) and predicted a highly homogeneous field in the central region of the bore. At the mid-height of the solenoid, the field was predominantly axial, with field lines running essentially in parallel and being very evenly distributed across the bore cross-section. As expected for a finite solenoid, increasingly pronounced deviations from parallelism occurred only toward the open ends of the bore, where the field lines gradually spread radially and form the characteristic fringe field outside the solenoid (Figure 1A,B; the schematic in Figure 1A is intentionally simplified for visualization). Our experimental geometry was specifically optimized to minimize exposure to these edge effects: the MFCs were positioned in the center of the culture dishes, and the dishes themselves were precisely centered on the longitudinal axis of the solenoid within the sample holder (Figure 1B). Consequently, the compartmentalized axons analyzed in our experiments were located in the central region of the bore and in close proximity to its longitudinal axis, where deviations of the field direction from axial alignment were negligible. We verified the predicted field distribution along the entire inner central axis of the solenoid using a calibrated manual Hall sensor at multiple positions. The measured field strength remained within 10 ± 0.5 mT along the complete measurement trajectory, corresponding to a maximum spatial variation of only ±5% around the nominal field strength. Notably, even the lowest field strength measured along the central axis (9.5 mT) remained well above the approximately 7 mT threshold at which the hyper-stimulated phenotype emerged, and therefore fully within the robust response plateau (see Results). Thus, the minor spatial variations in field strength within the central bore were unlikely to account for, or substantially influence, the pronounced and reproducible phenotypic effects observed upon ACMS. Moreover, the absence of ferromagnetic and magnetostrictive materials in our solenoids and incubator (interior made of non-magnetic stainless steel, Figure 1G) excluded magnetostriction and magnetically induced vibrations of ferromagnetic components. In the air-core copper solenoid, the only remaining intrinsic electromagnetic source of mechanical vibration was the Lorentz force acting on the current-carrying windings. However, we tested directly whether such vibrations at optimized ACMS conditions exerted any ad hoc effect on axonal organelle motility using a solenoid with similar heat dissipation and water-cooling that was placed directly on the sample holder of the live microscope (see Results). When the solenoid was switched on during the movie recording of so far untreated MNs, we neither observed any blur or jitter arising from vibrations, nor any change in the analyzed motility parameters, thereby arguing against temperature changes or other nonspecific effects of operating the solenoid as the primary explanation for the sustained biological responses observed at later times after accomplishment of all ACMS sessions, as shown in Figure 1J.

Alternating magnetic fields were generated by supplying the solenoids with alternating current (AC) from a function generator connected to a power amplifier (Figure 1F). As a refinement of our previously reported stimulation approach [18], sinusoidal rather than square-wave signals were used. Square waves contain multiple odd harmonics of the fundamental frequency, making it difficult to assign potential biological effects to a single frequency component. For example, a rectangular waveform with a fundamental frequency of 10 Hz contains harmonic components at 10, 30, 50, 70 Hz, and higher odd multiples, with relative amplitudes of 1, 1/3, 1/5, 1/7, and so forth, progressively decreasing for higher harmonics (Figure S1). In contrast, a sinusoidal waveform is characterized by a single defined frequency and amplitude, thereby facilitating reproducibility and a clearer interpretation of frequency-dependent biological effects (Figure S1). Unless stated otherwise (Figure 2E), alternating current magnetic stimulation (ACMS) was applied using a biphasic sinusoidal waveform with a time-averaged mean magnetic field strength of 10 mT. The stimulation protocol consisted of four treatment sessions separated by field-free resting intervals, as illustrated in Figure 1J. Frequency and waveform were continuously verified using an oscilloscope, whereas the magnetic field strength was monitored with a Gauss meter. The first three ACMS sessions were each performed continuously for 7 h, whereas the fourth and final session lasted 3.5 h. Treatments were initiated at DIV 30 and subsequently repeated at DIV 35, DIV 40, and DIV 45, with four-day stimulation-free intervals between consecutive sessions (Figure 1J). The first exposure was initiated after the human MN cultures derived from patients with ALS-associated FUS mutations had already developed characteristic disease-associated phenotypes, including impaired axonal organelle trafficking [15]. Following the final stimulation session, cultures were maintained for an additional two days without magnetic field exposure. The sustained effects of ACMS were subsequently evaluated at DIV 48 by live-cell imaging, as described below.

### 2.7. Imaging of MNs in MFCs

Time-lapse imaging was performed as described previously [15,46]. For simultaneous visualization and tracking of lysosomes and mitochondria, live MN cultures were labeled with LysoTracker™ Red DND-99 (Thermo Fisher Scientific Inc., Waltham, MA, USA, cat. no. L7528) and MitoTracker™ Deep Red FM (Thermo Fisher Scientific Inc., cat. no. M22426), each at a final concentration of 50 nM. Dyes were prepared from 1 mM DMSO stock solutions, added directly to the culture medium, and incubated for 1 h at 37 °C. Live-cell imaging was subsequently performed without further washing. Time-lapse microscopy was conducted at the light microscopy facility of the Center for Molecular and Cellular Bioengineering (CMCB), Technische Universität Dresden, using an inverted Leica DMI6000 fluorescence microscope (Leica Microsystems GmbH, Wetzlar, Germany) equipped with a Leica HC PL APO 100×/1.46 oil-immersion objective and a 12-bit Andor iXON 897 EMCCD camera (Andor Technology Ltd., Belfast, Northern Ireland, UK). Cultures were maintained at 37 °C and 5% CO_2_ under humidified conditions. Images were acquired at 512 × 512 pixels, corresponding to an effective sample-plane pixel size of 229.55 nm at 100× magnification after 0.7× intermediate demagnification. Sequential dual-channel recordings were acquired at 3.3 frames per second per channel over 2 min, yielding 400 images per channel, with an exposure time of 115 ms. LysoTracker™ Red was excited at 561 nm and detected using a TRITC 605/65-nm emission filter, whereas MitoTracker™ Deep Red was excited at 633 nm and detected through a Cy5 720/60-nm filter. Rapid sequential switching between the corresponding laser lines and emission filters using a motorized filter wheel prevented spectral crosstalk between the probes. Immunofluorescent (IF) stainings after ACMS and fixation at 48 DIV (Figure 1J) were carried out with fluorescently labeled secondary antibodies following standard protocols [15]. Glial fibrillary acidic protein (GFAP) was revealed with chicken polyclonal antibody ab4674 from Abcam Ltd. (Cambridge, UK) diluted 1:1000, neurofilament heavy chain (NF-H) was revealed with mouse monoclonal antibody BLD-801701 from BIOZOL Diagnostica Vertrieb GmbH (Hamburg, Germany) against the non-phosphorylated epitope SMI32 of NF-H diluted 1:1000, and nuclei were revealed with Hoechst 33342 from any provider at a final concentration of 0.5 µg/ml. Confocal images of IF stainings were acquired on an Evident (formerly Olympus) FLUOVIEW FV4000 microscope (Evident Corporation, Tokyo, Japan) with a silicon oil 60×/NA 1.2 apochromatic objective at identical settings for all cell lines and conditions during acquisition and post-processing (brightness, contrast) for figure preparations.

### 2.8. Organelle Tracking Analysis of Live Imaging Movies

**Figure 3:**
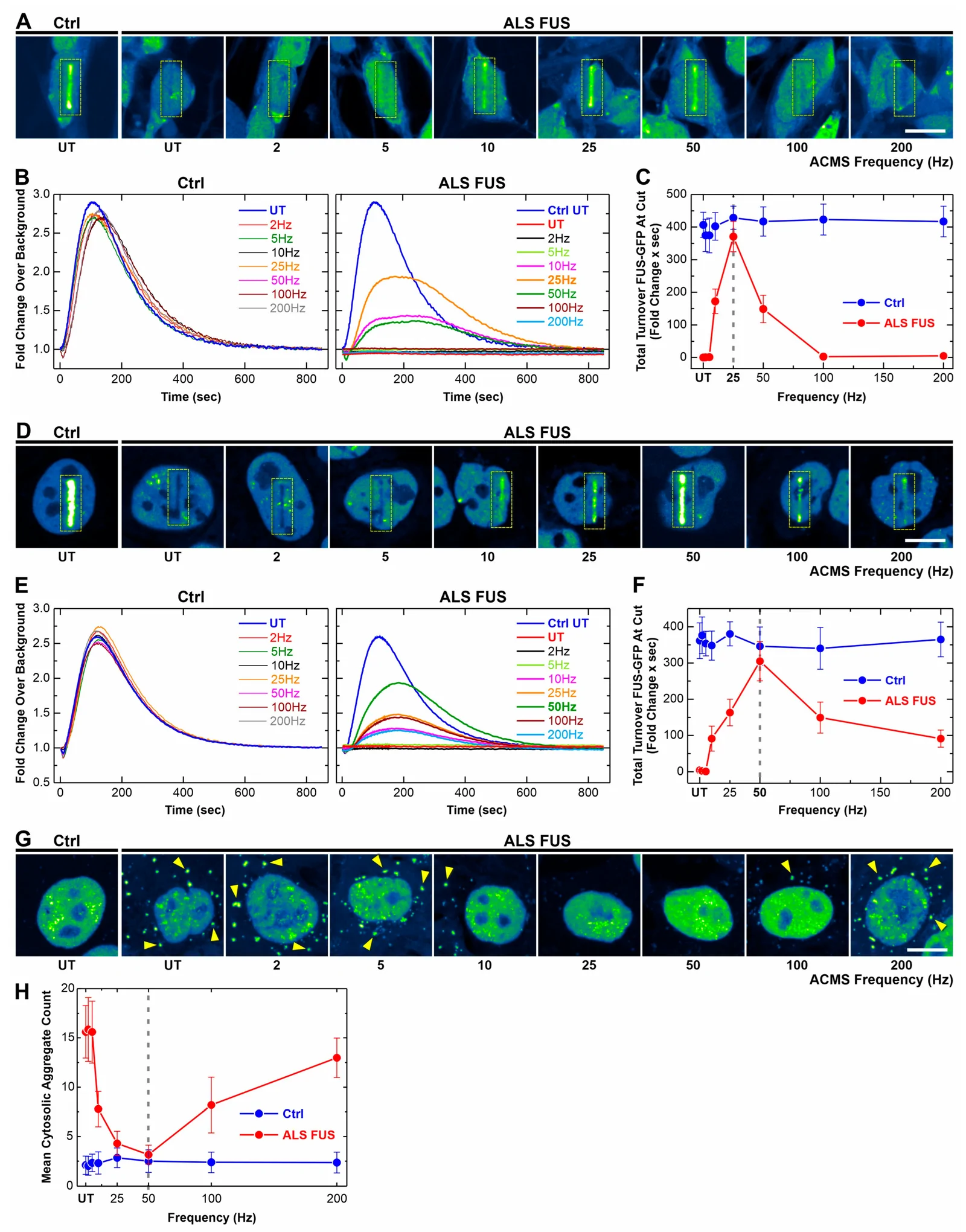
ACMS-mediated rescue of axonal organelle motility occurs concomitantly with a rescue of nuclear DDR. (**A**) Gallery of live images of the isogenic pair of FUS-GFP WT (Ctrl) versus the mutant FUS-GFP P525L MNs (ALS-FUS) (Table 1) at 48 DIV, as shown in Figure 1J, after the final ACMS session. All four consecutive ACMS sessions were performed as shown in Figure 1J at 10 mT and the indicated AC frequency at perpendicular orientation (Figure 1A–C), respectively, and compared against untreated (UT) cells. Recruitment-withdrawal of FUS-GFP to UV Laser cuts in nuclei (DDS, boxed area) was imaged live. Shown are single movie frames at 150 s after Laser irradiation when the eGFP intensity was around its maximum. The eGFP intensity is shown in the LUT “Green Fire Blue” of the FIJI software, i.e., low eGFP intensities are shown in blue and high intensities in green shades; no nuclear HOECHST staining or other fluorescent dyes were used. Each viewing field centers a single nucleus with its surrounding cytosol and protruding neurites. Note the failed recruitment in untreated ALS-FUS as compared to untreated Ctrl cells and its rescue through ACMS depending on the frequency, with an apparent maximum around 25 Hz. In case of failed FUS-eGFP recruitment (e.g., ALS-FUS UT), the Laser beam left a dark line due to photobleaching that is not to be mistaken for FUS-eGFP withdrawal from the DNA damage site. Refers to Movie S5. Scale bar = 10 µm. (**B**) Quantification of panel (**A**) as ratio fold change of eGFP intensity at the DDS over nuclear background (*y*-axis) plotted over time (*x*-axis). Note the failed recruitment in untreated ALS-FUS (**right**, red curve UT, flat line) over the entire recording time of 850 s whereas ACMS at 25 Hz led to the best partial rescue of the recruitment-withdrawal toward Ctrl kinetics (**right**, compare orange against blue Ctrl reference curve UT), whereas the physiological recruitment-withdrawal of FUS-eGFP in Ctrl cells was not further altered at any ACMS frequency (**left**). (**C**) Quantification of the total turnover of FUS-eGFP at the DDS. The entire area (integrals) underneath the recruitment-withdrawal curves in panel (**B**) after Laser irradiation was taken as a measure of total FUS-eGFP turnover at the DDS (*y*-axis) and plotted as a function of ACMS frequency. Data points are the means over all analyzed curve integrals for a given cell line at a specific frequency (*N* = 100, i.e., 100 curve integrals from three independent experiments were analyzed), with error bars showing the standard deviations. Note the maximum at 25 Hz in ALS-FUS (red curve), whereas the physiological turnover in Ctrl cells (blue curve) was not further altered at any ACMS frequency. (**D**) Similar to panel (**A**), except that the transgenic BAC HeLa lines stably expressing either FUS-GFP WT (Ctrl) or FUS-GFP P525L (ALS-FUS) (Table 1) were utilized to perform ACMS at the indicated frequency and then imaged to assess the DDR after Laser irradiation. Note the phenotypical similarity between the HeLa and motoneuronal ALS-FUS model (panel (**A**)), except that the optimal ACMS frequency was seemingly shifted to 50 Hz. Refers to Movie S6. Scale bar = 10 µm. (**E**) Quantification of panel (**D**), similar to panel (**B**), except that the optimal ACMS frequency for rescue was at 50 Hz (**right**; compare dark green against blue Ctrl reference curve UT). (**F**) Quantification similar to panel (**C**), except that the optimal ACMS frequency for rescue was at 50 Hz (red curve). (**G**) ACMS rescues from cytosolic FUS aggregation. Shown are maximum intensity projections of confocal Z-stacks imaged live in the transgenic BAC HeLa cell model expressing normal FUS-eGFP WT (Ctrl) or mutant FUS-eGFP P525L (ALS-FUS) (Table 1) after the final ACMS session similar to panels (**A**,**D**). All four consecutive ACMS sessions were performed as shown in Figure 1J at 10 mT and the indicated AC frequency at perpendicular orientation (Figure 1A–C), respectively, and compared against untreated (UT) cells. The eGFP intensity is shown in the LUT “Green Fire Blue” of the FIJI software, i.e., low eGFP intensities are shown in blue and high intensities in green shades; no nuclear HOECHST staining or any other fluorescent dye was used. Each viewing field centers a single nucleus with its surrounding cytosol. The cytosol, particularly in Ctrl cells, appears very dark, similar to the intercellular blank background due to its low content of eFUS-GFP. Note the occurrence of cytosolic FUS aggregates in untreated ALS-FUS as compared to untreated Ctrl Mock cells (arrowheads) that were rescued through ACMS, depending on the frequency, with an apparent maximum around 50 Hz. Scale bar = 10 µm. (**H**) Quantification of panel (**G**) as the count of cytosolic FUS aggregates (*y*-axis) plotted as a function of ACMS frequency (*x*-axis). Data points are the means of cytosolic aggregate counts per cell across all analyzed images (*N* = 100) for a given cell line at a specific frequency, with error bars presenting the standard deviations. Note the optimal rescue at 50 Hz in mutant ALS-FUS cells (red curve), whereas the low, physiological level in Ctrl cells was not further altered at any ACMS frequency (blue curve).

A comprehensive description of the automated analysis pipeline used in this study has recently been published [46], covering the entire workflow from object detection in raw time-lapse recordings to the quantitative characterization of organelle motility and morphology. Briefly, fluorescently labeled lysosomes and mitochondria were identified and tracked using the TrackMate plugin v7.9.2 in FIJI (ImageJ v1.53t, National Institute of Health, Bethesda, MD, USA). For each individual organelle, the analysis provided mean speed and track displacement, whereas the fraction of mobile organelles and the ratio of anterograde-to-retrograde trajectories were determined on a per-movie basis. Further details of these analyses are provided in the Results section. Individual movie-based output files were subsequently processed and integrated using KNIME v4.7.0 (KNIME Analytics Platform, KNIME AG, Zurich, Switzerland) to generate final datasets containing annotated organelle-specific parameters. This workflow enabled pooling of all measurements obtained under identical experimental conditions, for example, mitochondria- or lysosome-derived measurements acquired at the distal microchannel position from a defined cell line exposed to a specific treatment condition, such as ACMS at 25 Hz. Depending on the dataset, this bulk analysis typically comprised tens of thousands of individual organelles per pooled experimental condition. Data presented in Figure 2B–E, Figure 3C,F,H, Figures S3B–E, S4B–E, S5B–E, S6B–E and S7D–G were pooled from four independent experiments. The data shown in Figure S2 were derived from one representative experiment selected from four independent experiments. All other data shown are representative examples.

### 2.9. DNA Damage Laser Irradiation Assay

Isogenic FUS-GFP WT and FUS-GFP P525L spinal MNs (Table 1) were differentiated from NPCs as described above (Figure 1H), dissociated, and seeded at 300,000 cells per 3.5-cm culture dish instead of into microfluidic chambers. Dishes were pre-coated with poly-L-lysine and laminin as described previously [15]. DDR experiments were performed at DIV 48 following the ACMS protocol shown in Figure 1J [15,47]. Briefly, a focused 355 nm UV laser beam was directed to defined x–y–z coordinates using a stereotactic galvanometric mirror system coupled to an inverted Zeiss Axio Observer Z1 microscope (Carl Zeiss Microscopy GmbH, Jena, Germany) with a motorized stage and piezoelectric z-actuator. Using a Zeiss alpha Plan-Fluar 100×/1.45 oil-immersion objective, 24 laser pulses at 0.5 µm intervals generated 12 µm linear DNA damage tracks within individual nuclei. Rapid recruitment of FUS-GFP to the irradiated sites and its subsequent slower dissociation were recorded as characteristic FUS on–off kinetics by live spinning-disk confocal imaging for 15 min. GFP was imaged using a 488 nm laser and a 12-bit Andor iXON 897 EMCCD camera (512 × 512 pixels; effective sample-plane pixel size, 229.55 nm at 100× magnification), initially at 1 fps and subsequently at 0.2 fps during the slower dissociation phase. For quantitative analysis, integrated GFP fluorescence at the laser-induced damage tracks was measured in FIJI, normalized to the surrounding nuclear background, and plotted as fold change over time to visualize FUS recruitment and withdrawal kinetics (Figure 3B,E).

### 2.10. Image/Movie Quantification

For analysis of cytosolic FUS-eGFP mislocalization in HeLa cells (Figure 3G,H), three independent experiments were performed, with at least 10 confocal z-stacks analyzed per experiment as described previously [15]. Standard object segmentation tools of FIJI (“Analyse Particles…”) were used to quantify the counts of discrete cytosolic FUS-positive objects. Aggregate counts per image were pooled across all images and experiments (*N* = 30) and plotted as mean values over ACMS frequency, with error bars indicating standard deviations (Figure 3H). For organelle tracking in MFC cultures, at least five movies were acquired per MFC, each representing one biological replicate. Three MFCs were analyzed per experiment and four independent MN differentiation experiments were performed per cell line, typically yielding pooled datasets comprising tens of thousands of organelles. To eliminate any potential bias in our analysis, we took the following measures: (i) all acquired images and movies were included in the analysis, i.e., no visual inspection or preselection of data was performed; (ii) cell samples provided to the person operating the microscope were labeled with a blinding code instead of the original cell line name, and unblinding was performed only after completion of the image acquisition and analysis; (iii) identical settings and thresholds were applied across all cell lines and conditions during data acquisition, analysis and post-processings; (iv) movies were consistently acquired at highly standardized MFC positions at the distal end of the microchannels (Figure 1I); (v) organelle tracking and the subsequent compilation of the complete result tables were performed in an automated manner using the FIJI TrackMate plugin and KNIME as described [15,46].

### 2.11. RNA Extraction

Total RNA from entire MFC MN cultures was extracted and purified at 48 DIV (Figure 1J) either after ACMS at 25 Hz, perpendicular field orientation (Figure 1A–C) and 10 mT or from untreated controls (UT). The preparation was performed with the QIAGEN RNeasy^®^ Plus kit (QIAGEN GmbH, Hilden, Germany, cat no. 74134) according to the manufacturer’s manual and typically resulted in 5–10 µg of total RNA dissolved in a total volume of 80 µL of DNAse/RNAse-free water. For each cell line (four control and four mutant ALS-FUS lines, eight lines in total, Table 1) and condition (ACMS versus UT), four biological replicates (=MFC cultures) were used, resulting in 16 conditions (eight lines, each ACMS versus UT) and altogether 64 samples (16 conditions, each with four biological replicates). Concentration and integrity of each RNA sample were analyzed on an Agilent^®^ 2100 Bioanalyzer (Agilent Technologies, Inc., Santa Clara, CA, USA, assay class: eukaryote total RNA nano, version 2.6); all resultant RNA integrity numbers (RIN) were in the range of 8.7–9.7, indicating high purity and integrity of all samples.

### 2.12. Library Preparation

Messenger RNA was isolated from 500 ng of each total RNA sample (see above) by poly-dT enrichment using the New England Biolabs^®^ Inc. Next Poly-A mRNA Magnetic Isolation Module (NEB, Ipswich, MA, USA, cat. no. E7490L) according to the manufacturer’s instructions. Samples were then directly subjected to the workflow for strand-specific RNA-seq library preparation (Ultra II Directional RNA Library Prep, NEB, cat. no. E7765L). For ligation, Next Adapters for Illumina from the NEB Next Multiplex Oligos for Illumina kit were used (NEB, cat. no. E7335). After ligation, adapters were depleted using XP bead purification (Beckman Colter, Indianapolis, IN, USA, cat. no. A63881) by adding the bead solution in a ratio of 0.9:1 to the samples. Unique dual indexing was performed during the following PCR enrichment (12 cycles) using amplification primers carrying the same sequence for i7 and i5 index (i5: AAT GAT ACG GCG ACC ACC GAG ATC TAC AC NNNNNNNN ACA TCT TTC CCT ACA CGA CGC TCT TCC GAT CT, i7: CAA GCA GAA GAC GGC ATA CGA GAT NNNNNNNN GTG ACT GGA GTT CAG ACG TGT GCT CTT CCG ATC T). After two more XP bead purifications (0.9:1), libraries were quantified using the Fragment Analyzer (Agilent^®^).

### 2.13. RNA Sequencing and Differential Gene Expression Analysis

Libraries from above were sequenced on an Illumina^®^ NovaSeq 6000 (Illumina Inc., San Diego, CA, USA) in 100 bp paired-end mode to a depth of 40 million fragments (short reads) per library. Short reads were aligned to the human reference genome hg38 with support of the Ensembl genome annotation (release 104, EMBL-European Bioinformatics Institute [EMBL-EBI], Hinxton, UK) [48] splice sites using the aligner STAR v2.7.10b (Alexander Dobin, Cold Spring Harbor Laboratory, Cold Spring Harbor, NY, USA) [49], and the custom settings “--outSAMmultNmax 1 --sjdbOverhang 100”. Counts per gene and sample were obtained based on the overlap of the uniquely mapped fragments with the same Ensembl annotation using featureCounts v2.0.1 (Subread package, Walter and Eliza Hall Institute of Medical Research, Parkville, VIC, Australia) [50], and the settings “-s 2 -t ‘exon’ -p -Q 1”. Normalization of raw fragments based on library size and testing for differential expression between the different conditions were done with the DESeq2 R package (DESeq2: v1.38.3, Michael Love, Simon Anders, Wolfgang Huber, Bioconductor project, European Molecular Biology Laboratory [EMBL], Heidelberg, Germany; R package: v4.2.2, R Foundation for Statistical Computing, Vienna, Austria) [51]. Principal Component Analysis (PCA) based on the 500 most variable genes was performed in R package to explore the relationship between biological replicates and different libraries. DEGs were determined using the Wald test of DESeq2. Resulting adjusted *p*-values of DEGs were calculated from the variance across the four biological replicates of each condition (see above) and corrected for multiple testing with the Independent Hypothesis Weighting package (IHW 1.26.0, Nikos Ignatiadis, Wolfgang Huber, Bioconductor project, EMBL) [52]. Genes with a maximum false discovery rate of 5% (adjusted *p*-value ≤ 0.05) and |log2FC| ≥ ± 1 were considered significantly differentially expressed. DEGs were calculated for each cell line for ACMS versus UT, respectively, as well as for FUS-GFP P525L UT versus isogenic FUS-GFP WT UT. Full differential expression analysis results are provided in Table S1 along with a complete, unfiltered ranked gene list in Table S2 using the standard GSEA ranking metric calculated from our DESeq2 analysis:

sign(log2FC) × [−log10(adjusted *p*-value)].

### 2.14. Functional Enrichment Analysis of DEGs

To investigate the biological relevance of genes showing differential expression in the ALS-FUS model, functional enrichment analyses were performed with the Enrichr web platform (Ma’ayan Laboratory, Icahn School of Medicine at Mount Sinai, New York, NY, USA, https://maayanlab.cloud/Enrichr/, accessed on 20 June 2025) [53]. GO enrichment was assessed separately for the biological process (BP), molecular function (MF), and cellular component (CC) ontologies. In addition, pathway enrichment was evaluated using the Kyoto Encyclopedia of Genes and Genomes (KEGG, Kanehisa Laboratories, Kyoto, Japan) and Reactome (Reactome Project, EMBL-EBI, Hinxton, UK) databases. Analyses were performed using the default settings of Enrichr. GO terms and pathways with *p* ≤ 0.05 were considered significantly enriched and were subsequently evaluated according to their biological relevance. Differentially expressed target genes meeting the criteria of an absolute fold change of at least 2 and an adjusted *p*-value ≤ 0.05 were extracted using Partek™ Flow v11.0 (Partek Inc., San Diego, CA, USA) and used to generate Venn diagrams identifying overlapping candidate genes for downstream comparison of non-isogenic ALS model cultures.

### 2.15. Gene Set Enrichment Analysis (GSEA)

Gene set enrichment analysis (GSEA) [54] was performed to identify coordinated changes in biological pathways and functional gene sets without restricting the analysis to individually selected DEGs. Normalized RNA-seq count data generated with DESeq2 were analyzed in Partek™ Flow v11.0. Comparisons between mutant and control samples (each ACMS versus untreated) were evaluated against GO-derived gene set collections representing biological process, molecular function, and cellular component categories, as well as the KEGG pathway database. Genes were ranked using the signal-to-noise metric. Enriched gene sets and pathways were considered significant when they reached a normalized enrichment score (|NES|) of at least 1 together with *p* ≤ 0.05.

### 2.16. Protein–Protein Interaction (PPI) Network Construction and Identification of Key Genes

A PPI network was established for differentially expressed and functionally related genes (DEFRGs) using the STRING database (STRING Consortium, University of Zurich, Zurich, Switzerland, https://string-db.org/, accessed on 23 June 2025) [55]. Interactions were included at a minimum required confidence score of 0.4. Within the resulting network, nodes represent the DEFRGs/proteins, whereas edges denote predicted functional associations between pairs of proteins. These associations were supported by evidence from curated databases, high-throughput experiments, co-expression or co-occurrence analyses, text mining, and genomic neighborhood information. The PPI network was subsequently transferred to Cytoscape v3.10.3 (Cytoscape Consortium, San Diego, CA, USA, https://cytoscape.org/, accessed on 24 June 2025) [56] for network visualization and topological analysis. Key network nodes were identified using the maximal clique centrality (MCC) algorithm implemented in the cytoHubba plugin (Institute of Information Science, Academia Sinica, Taipei, Taiwan) [57], which enables the ranking of important nodes and subnetworks based on network topology. The 15 genes with the highest MCC scores were selected as hub genes in the present study.

### 2.17. Statistical Analysis

All graphical plotting and statistical analysis (ANOVA, Bonferroni post hoc tests, Student’s *t*-tests) for organelle motility and DDR were performed with OriginPro software v2021b (64-bit) SR2 9.8.5.212 (OriginLab Corporation, Northampton, MA, USA). The general pipeline for RNA-seq analysis used in this study, including alignment, quantification, normalization, and differential gene expression analysis, as well as statistical analyses including Venn comparisons, GSEA, PCA, Hierarchical clustering (with Euclidean distance measure and average linkage clustering), visualization maps, and QC assessment of differentially expressed mRNAs, was performed using the RStudio v2024.09.0 (Posit Software, PBC, Boston, MA, USA) and Partek™ Flow™ software v11.0 to aid the visualization and interpretation of the expression patterns of DEGs. Differential expression was analyzed by applying an adjusted *p*-value ≤ 0.05 to the DESeq2 results, followed by false discovery rate (FDR) step-up for multiple comparisons. Genes with FDR/adjusted *p*-value ≤ 0.05 and log2FC ≥ 1 or log2FC ≤ −1 were considered significant for the identification of DEGs. All of the gene expression samples presented in this study were designed with a minimum of 3 biological replicates (mean ± standard error (SEM), n ≥ 3). For the functional enrichment analysis, significantly enriched GO terms, pathways, and PPI networks were identified using adjusted *p*-values ≤ 0.05 as the cutoff value for statistical significance. GSEA was performed in Partek™ Flow™ software v11.0 using normalized gene count values. Validation data were plotted and statistically analyzed on GraphPad Prism v9.4.1 (GraphPad Software, Boston, MA, USA). Differences were considered statistically significant for *p*-values < 0.05. *p*-values were depicted in graphs as *, **, ***, **** representing *p* ≤ 0.05, *p* ≤ 0.01, *p* ≤ 0.001, and *p* ≤ 0.0001, respectively.

### 2.18. Declaration of Generative AI and AI-Assisted Technologies in the Manuscript Preparation Process

During the preparation of this manuscript, the author(s) used ChatGPT powered by the GPT-5.5 language mode (OpenAI, San Francisco, CA, USA) for stylistic improvements, greater concision and clarity, and condensing text modules by removing redundancies. The authors reviewed and edited the output and take full responsibility for the content of the published article.

## 3. Results

We selected an isogenic pair of iPSC-derived spinal MN lines expressing either wild-type FUS or the clinically severe P525L point mutation in the carboxyterminal NLS domain, fused to eGFP (FUS-GFP WT and FUS-GFP P525L, Table 1) as previously described [15,18,47]. This isogenic design ensured that all phenotypic differences observed between mutant and control MNs, particularly in response to ACMS treatment, were attributable to the FUS-NLS mutation rather than differences in genetic background. We confirmed that both lines displayed a comparable propensity to express MN markers during differentiation and maturation, and that distal axonal trafficking perturbations in the mutant line were consistent with those described for other non-isogenic FUS-NLS mutants [15]. Using our established live-cell imaging assays of compartmentalized MNs with aligned axons in Xona microfluidic chamber cultures (MFCs) (Figure 1H–J), we evaluated the effects of ACMS field orientation, frequency, amplitude, and duration on axonal organelle trafficking and DDR. The culture system allowed precise alignment of the magnetic field relative to axonal orientation and physical segregation of axons from the somatodendritic compartment (Figure 1I). At the time of ACMS treatment and subsequent assays, all lines reliably produced axon-pure neurites at the distal compartment of MFCs (Figure 1H–J).

The eGFP tag enabled real-time imaging of the recruitment-withdrawal cycle of FUS at nuclear DNA damage sites (DDSs) following laser microirradiation, allowing us to assess DNA damage response (DDR) kinetics in living cells as previously established [15,47]. This assay has revealed the failure of FUS-GFP P525L mutant MNs to recruit FUS to DDSs [15,47].

Starting from neuronal progenitor cells (NPCs) pre-differentiated from iPSCs, we employed our established in vitro differentiation pipeline to generate spinal MNs, which were subsequently seeded and matured in MFCs (Figure 1H) as previously described [15]. At 30 days in vitro (DIV) after initiation of NPC differentiation, a time point at which FUS-GFP P525L MNs and other FUS-NLS mutants exhibit fully developed phenotypes, including severely impaired distal axonal organelle motility, abolished FUS recruitment to DDSs, along with its aberrant nucleo-cytosolic shuttling and increased DNA damage accumulation [15], we initiated ACMS treatment.

The ACMS protocol consisted of four consecutive stimulation sessions (three sessions of 7 h and one session of 3.5 h) separated by four-day resting intervals (Figure 1J), as previously established [18]. For ACMS delivery, up to six Petri dishes containing individual MFCs were placed in a custom-tailored sample holder within the bore of an upright solenoid, allowing homogeneous field lines to be oriented perpendicular to the axonal growth plane (Figure 1A–C). In parallel experiments, Petri dishes were placed in a horizontal solenoid using an alternative holder to generate a magnetic field oriented parallel to the axonal plane (Figure 1A,D,E). All solenoids were housed inside a standard cell culture incubator, and heat generated during ACMS was dissipated via water-cooled tubing connected to an external thermostat to maintain a stable temperature of 37 °C (Figure 1B–G). Alternating sine-wave currents of defined frequency and amplitude were generated and amplified to achieve magnetic field strengths typically of 10 mT (Figure 1F).

Fast dual-channel live imaging of distal and proximal compartments of MFCs was performed at 48 DIV, i.e., two days after the final ACMS session at 45 DIV (Figure 1J), to assess sustained effects of the stimulation protocol with potential clinical relevance [18]. We first optimized ACMS parameters by varying the frequency of the alternating magnetic field from 0 Hz (untreated controls, UT) to 200 Hz, while maintaining a constant field strength of 10 mT and perpendicular field orientation. Specifically, all four consecutive ACMS sessions were performed at 0, 2, 5, 10, 25, 50, 100, or 200 Hz, respectively, on distinct MFC cultures, followed by live imaging of lysosomes and mitochondria using mito- and lysotracker dyes to quantify axonal organelle motility (Figure 1J).

Axonal organelle motility was quantified using our established semi-automated tracking pipeline [15,46], which deduces four key parameters from raw movie frames: (i) mean speed, (ii) track displacement, (iii) moving fraction (defined as the proportion of organelles exceeding a track displacement threshold of 1.2 µm), and (iv) the ratio of anterograde-to-retrograde transport events as a measure of net directional bias. Track displacement served as a measure for effective, processive transport, distinguishing sustained directional movement from non-processive jitter.

In untreated MNs, FUS-GFP P525L (ALS-FUS) exhibited a pronounced reduction in lysosomal mean speed (~0.4 µm/s) compared to FUS-GFP WT (Ctrl, ~1.1 µm/s) in distal axons, as well as a comparable reduction in mitochondrial mean speed (~0.4 µm/s versus ~0.7 µm/s in Ctrl), consistent with previous observations [15,47] (Figure S2). Both cell lines displayed characteristic top-tailed speed distributions, with lysosomes generally moving faster than mitochondria.

ACMS induced a robust, frequency-dependent increase in axonal organelle motility in both genotypes (Figure S2). A striking optimum was observed at 25 Hz, whereas stimulation at 2 Hz or 200 Hz had no appreciable effect relative to untreated controls. At 25 Hz, mean lysosomal speed in mutant MNs reached ~1.5 µm/s, exceeding untreated control levels, and mitochondrial speeds increased to ~1.0 µm/s (Figure S2). Control MNs similarly exhibited stimulation beyond physiological baseline levels at this frequency. Thus, ACMS did not merely restore mutant motility to control levels but instead induced a hyper-physiological motility state common to both genotypes. For clarity, subsequent analyses are presented as population averages with standard deviations (error bars) rather than box plots (Figure 2A,B and Figure S3A,B, Movie S1).

Increases in mean speed were accompanied by concordant increases in track displacement and moving fraction, again peaking at 25 Hz for both lysosomes and mitochondria (Figure S3C,D). Analysis of net transport direction revealed a baseline anterograde bias in untreated MNs of both genotypes, with anterograde-to-retrograde ratios of ~1.6 for lysosomes and ~1.4 for mitochondria (Figure S3E). ACMS at 25 Hz reversed this bias, resulting in a net retrograde preference toward the somatodendritic compartment (Figure S3E).

We next examined the influence of magnetic field orientation relative to the axonal plane. Repeating the frequency series using parallel field orientation (Figure 1A–E) revealed markedly reduced stimulation of mean speed, track displacement, and moving fraction compared to perpendicular orientation (Figure 2C and Figure S4A–D, Movie S2). In control MNs, parallel orientation produced no appreciable effect at any frequency, whereas mutant MNs showed only a weak residual optimum at 25 Hz. Reversal of transport direction also occurred under parallel orientation but was substantially attenuated (Figure S4E). Given the superior efficiency of perpendicular orientation, the parallel configuration was rejected from subsequent experiments.

To assess the persistence of ACMS effects at optimal frequency (25 Hz), perpendicular orientation and 10 mT, we analyzed axonal organelle motility over a 14-day chase period following the final ACMS session at 45 DIV (Figure 1J). Hyper-physiological organelle velocities were already evident one day after treatment and remained stable for approximately 8 days in both genotypes, with lysosomal speeds of ~1.6 µm/s and mitochondrial speeds of ~1.1 µm/s (Figure 2D and Figure S5A,B, Movie S3). Thereafter, motility parameters declined rapidly between days 9 and 11, returning to untreated baseline levels. Track displacement, moving fraction, and net directional bias followed similar kinetics (Figure S5C–E).

Field-strength titration experiments were performed at optimal frequency (25 Hz) and perpendicular orientation, varying magnetic flux density from 0 to 14 mT in 1 mT increments. No changes in motility parameters were detected between 0 and 5 mT at 48 DIV (Figure 1J), whereas a sudden transition to the hyper-physiological motility state occurred at ≥7 mT for both lysosomes and mitochondria, with no further increases up to 14 mT (Figure 2E and Figure S6A–E, Movie S4). This behavior indicated a critical threshold rather than a gradual dose–response relationship, thereby pointing to a switch from either the physiological motility state (Ctrl) or the pathologically low state (ALS-FUS) to the harmonized hyper-physiological state common to both lines.

Given the established causal link between defective DDR and impaired axonal trafficking in ALS-FUS MNs [15], we investigated whether ACMS also affected DDR. Using laser-induced DNA damage followed by live imaging of FUS-GFP recruitment, we confirmed normal recruitment kinetics in untreated control MNs and a complete absence of recruitment in the untreated FUS-GFP P525L mutant (Figure 3A,B, Movie S5). ACMS (Figure 1J) at perpendicular field orientation (Figure 1A–C) induced a frequency-dependent partial restoration of FUS recruitment in mutant MNs, with a clear maximum at 25 Hz (Figure 3B,C, Movie S5), whereas control cells remained unchanged across all frequencies.

Our previous BAC HeLa cell model stably expressing FUS-GFP P525L exhibited a similar failure to recruit FUS to laser-induced DDS [47]. Remarkably, ACMS led to a similar DDR restoration in this model (Figure 3A–C), albeit with a shifted frequency optimum at 50 Hz (Figure 3D–F, Movie S6). Since we have previously linked the deficient DDR in the FUS P525L mutant to aberrant nucleo-cytosolic shuttling of FUS along with its cytosolic aggregation [15,47], we investigated the impact of ACMS on this disease hallmark as well. To this end, we took advantage of the BAC HeLa cell model, as its large, flat cytosol surrounding the nucleus greatly facilitates the quantification of such aggregates, whereas tight neuronal perikarya can pose technical problems. We revealed that ACMS also reduced cytosolic FUS aggregation in a frequency-dependent manner, restoring aggregate levels to those of WT control cells at the optimal frequency (Figure 3G,H) [47].

Finally, to test whether ACMS exerts direct ad hoc effects on the axonal transport machinery, we performed live imaging while applying ACMS on the microscope’s stage acutely during recording (25 Hz, 10 mT, perpendicular orientation) at 48 DIV (Figure 1J) on MNs so far untreated (Figure S7A,B). Specifically, we recorded axonal organelle motility over our established standard observation period of 2 min [15,46] while the solenoid was still switched off to obtain a reference movie of untreated MNs (Figure S7C, in gray). Then, we switched on the solenoid (Figure S7C, in red, Movie S7) and recorded movies over 2 min periods each over a time course of 480 min (i.e., 8 h) at time points as indicated in Figure S7D–G during ACMS. No immediate changes in mean speed, track displacement, moving fraction, or transport direction were detected over the entire 8 h stimulation period (Figure S7C–G, Movie S7). These findings exclude direct instantaneous magnetic field effects and support a model in which repeated ACMS sessions induce slower, sustained systemic gene expression changes underlying both enhanced axonal organelle motility and improved DDR.

### 3.1. A Harmonized, Novel Expression Profile Irrespective of Genotype as Common Cellular Responses to ACMS Across All Control and Mutant FUS Lines

**Figure 4:**
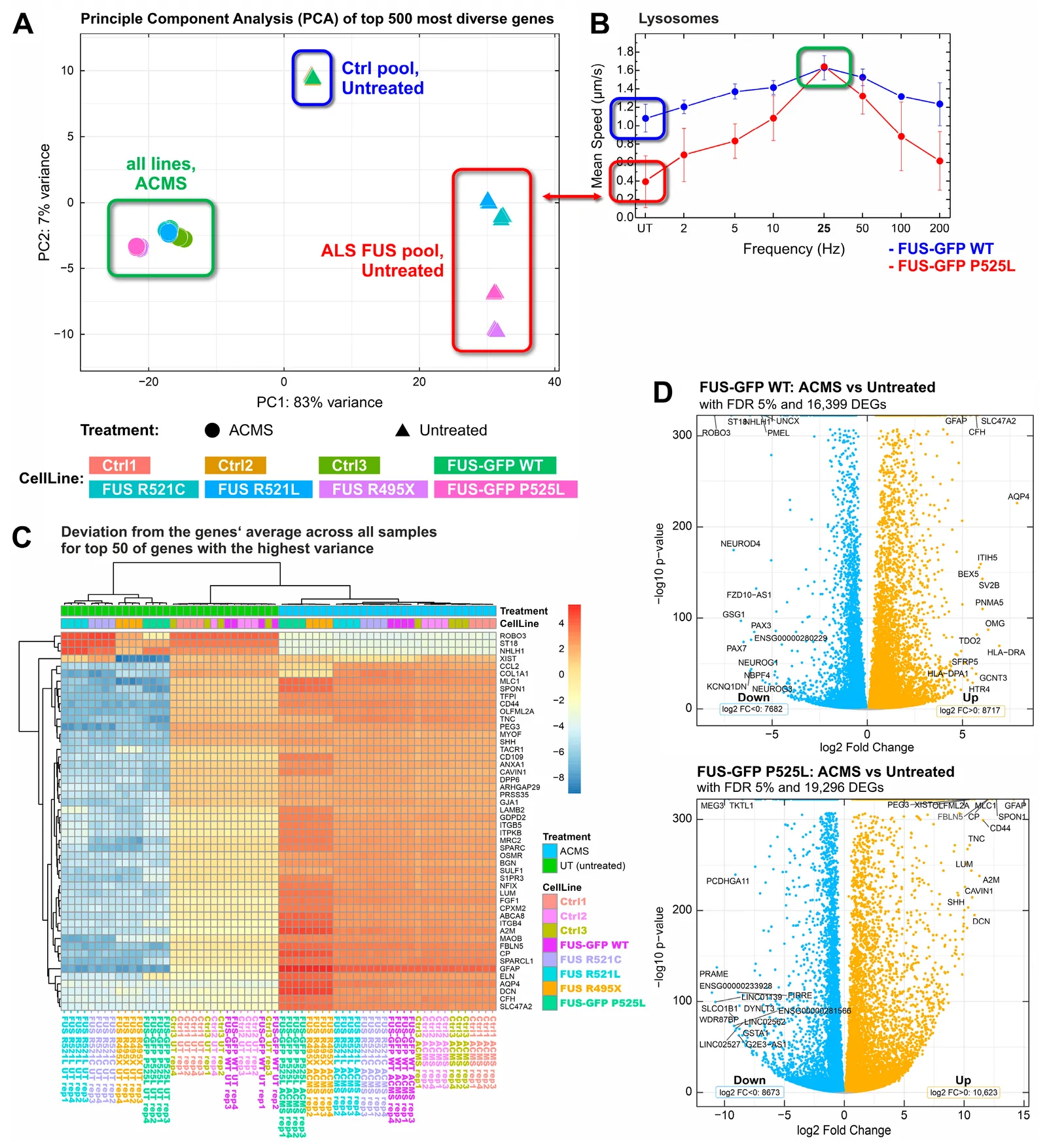
Transcriptomic data analysis. (**A**) Principal component analysis (PCA) of samples highlighting similarities and differences in gene expression between the various mutant FUS samples (isogenic and non-isogenic) compared to WT Ctrl samples. In the two-dimensional PCA plot, each condition (ACMS and Untreated) is represented as a circle/triangle; the closer the circles/triangles in the spreadsheet, the higher the similarity within the clustered samples. Two types of samples (isogenic and non-isogenic) from 8 different cell lines (4× WT Ctrl and 4× mutant FUS, see Table 1) are shown in this figure; different colors indicate different lines (with 4 biological replicates per line), while different symbols (circles/triangle) indicate different condition attributes (ACMS: circles; Untreated: triangles). Note the occurrence of only three discrete transcriptomic states: (i) all untreated WT Ctrl samples clustered closely together (encircled in blue), whereas (ii) all untreated mutant FUS samples appear in a distinct cluster (encircled in red), thereby indicating a common consensus gene expression profile under physiological conditions that was clearly distinct from the common (albeit more dispersed) pathological profiles. Once ACMS-treated, (iii) all lines regardless of genotype switched to a novel, distinct transcriptomic state (cluster encircled in green). (**B**) Mean speed of axonal organelles as a function of ACMS frequency, taking lysosomes as a representative example from FUS-GFP WT (Ctrl, in blue) versus FUS-GFP P525L (ALS-FUS, in red) MNs (panel taken from Figure 2B). Note how the three discrete transcriptomic states encircled in panel (**C**) clearly corresponded to the three discrete phenotypic states revealed by organelle tracking: (i) ~0.4 µm/s in untreated (UT) ALS-FUS MNs (pathological state encircled in red), (ii) ~1.1 µm/s in UT Ctrl MNs (physiological state encircled in blue), (iii) ~1.6 µm/s in all ACMS samples treated at the optimal 25 Hz regardless of genotype (harmonized hyper-physiological state encircled in green). (**C**) Heatmap of the top 50 genes with the highest mean expression strength across all samples. The scale goes from blue to red, representing the highest expression. (**D**) Volcano plot displaying the distribution of DEGs of isogenic FUS-GFP WT (**top**) and mutant FUS-GFP P525L MNs (**bottom**), each ACMS versus untreated: *p* ≤ 0.05, FDR ≤ 0.05 and log2 (Fold Change) ≥ 0.1 or log2 (Fold Change) ≤ −0.1.

Next, to obtain a global view of the transcriptome changes of the mutant ALS-FUS MNs in response to ACMS sessions, the expression profiles of ACMS-treated versus untreated samples were compared to their respective healthy controls (hereafter Ctrl) by RNA-seq. (Figure 1J). Briefly, total RNA was isolated from the entire MFC (pooled distal and proximal sites, Figure 1I) at 48 DIV after the final ACMS session (Figure 1J) at the optimal 25 Hz, perpendicular field orientation (Figure 1A–C), and 10 mT to capture gene expression changes, including possible non-cell autonomous contributions in the somatodendritic compartment. ACMS and RNA-seq were performed on the isogenic pair (FUS-GFP WT and FUS-GFP P525L) as well as on further non-isogenic three cell lines containing WT FUS and three lines from other ALS patients harboring distinct FUS-NLS mutations to assess genotype specificity versus universal responses (eight lines in total, Table 1, Material and Methods). Sequencing reads were then processed to generate normalized counts, and the libraries were compared using principal component analysis (PCA) (Figure 4A).

Principal component 1 (PC1, *x*-axis) accounted for 83%, while PC2 (*y*-axis) accounted for 7% of the variance. All untreated Ctrl samples grouped closely together (encircled in blue) and were clearly separated from untreated ALS-FUS samples (encircled in red), indicating a distinct physiological versus pathological transcriptomic state (Figure 4A). Conversely, all ACMS-treated samples were located in a distinctively novel PCA group in which Ctrl and mutant samples were no longer distinguishable from each other (encircled in green, Figure 4A). This remarkable transcriptomic harmonization corresponded well to the phenotypic harmonization through ACMS in our analysis of axonal organelle motility (Figure 2B and Figure 4B). In essence, the occurrence of the discrete pathological motility state (Figure 4B, ALS-FUS untreated, encircled in red) distinct from the physiological state (Figure 4B, Ctrl, encircled in blue) and the hyper-physiological state (Figure 4B, irrespective of genotype, encircled in green) was feasibly attributable to the respective transcriptomic PCA group (Figure 4A), thereby pointing to altered gene expression as the underlying cause for ACMS-mediated sustained improvements in axonal organelle motility. Unsupervised hierarchical clustering (i.e., dendrograms) using both Pearson and Spearman’s correlation revealed a clear separation between sample groups (isogenic and non-isogenic), with the top 50 genes of highest variance effectively partitioning the ACMS-treated MNs from untreated controls (Figure 4C and Figure S8A–C). The high degree of concordance between these clustering methods underscores the robustness of the expression signature and confirms that the identified genes represent the dominant biological response to ACMS treatment. In essence, the various types of hierarchical clustering (Figure 4C and Figure S8A–C) were in line with the separation of entire expression profiles across all samples into distinct groups in the PCA plot (Figure 4A).

To identify the underlying biological forces driving the remarkable convergence of ACMS-treated samples along the primary axis of variance, we examined the top feature weights (gene loadings) governing PC1 (Figure 4A). We extracted the top 500 variable genes showing the highest absolute loadings on PC1. To preserve biological directionality, we separated these 500 genes into their positive and negative loading directions and subjected the subsets (62 positive-loading and 438 negative-loading genes, Table S3) to pathway enrichment analysis using the EnrichR software against the Gene Ontology (GO)/pathway databases.

The 438 negative-loading genes, which contributed predominantly to the positioning and convergence of ACMS-treated samples along PC1, showed strong enrichment for extracellular matrix (ECM) organization and related pathways (Reactome, Padj = 1.04 × 10^−22^, Table S3, Figure S9). This signature comprised 46 prominent ECM-related structural and signaling components, including collagens (e.g., COL1A1, COL3A1, COL4A1), matrix receptors and adhesion molecules (e.g., ITGB3, ITGB4, ITGA2, CD44, VCAM1), basement-membrane components (e.g., LAMA4, LAMB2), proteoglycans (e.g., DCN, BGN, LUM), and remodeling-associated factors (e.g., SPARC, SERPINE1, TNC). In contrast, the 62 positive-loading genes were predominantly associated with developmental and neurogenic programs, including neural plate border specification (e.g., TFAP2A, PAX7, PAX3; Reactome, Padj = 2.69 × 10^−3^) and other pathways related to neurogenesis and transcriptional regulation (Table S3, Figure S9). A subset of 14 core genes was particularly prominent in these enriched developmental pathways (e.g., TFAP2A, ROBO3, PMEL; Reactome, Padj = 4.51 × 10^−3^).

Together, these analyses identify broad activation of ECM-related transcriptional programs, accompanied by fewer changes in a smaller set of developmental and neurogenic genes, as major contributors to the ACMS-associated shift along PC1. Importantly, PC1 represents a single shared mathematical vector derived from the entire dataset. Both control and mutant FUS lines shifted along this same axis following ACMS treatment, indicating that the transcriptional features contributing to PC1-associated convergence were shared across genotypes rather than being confined to a genotype-specific response. Thus, ACMS appears to engage a common transcriptional program superimposed on the distinct baseline states of control and mutant FUS MNs.

**Figure 5:**
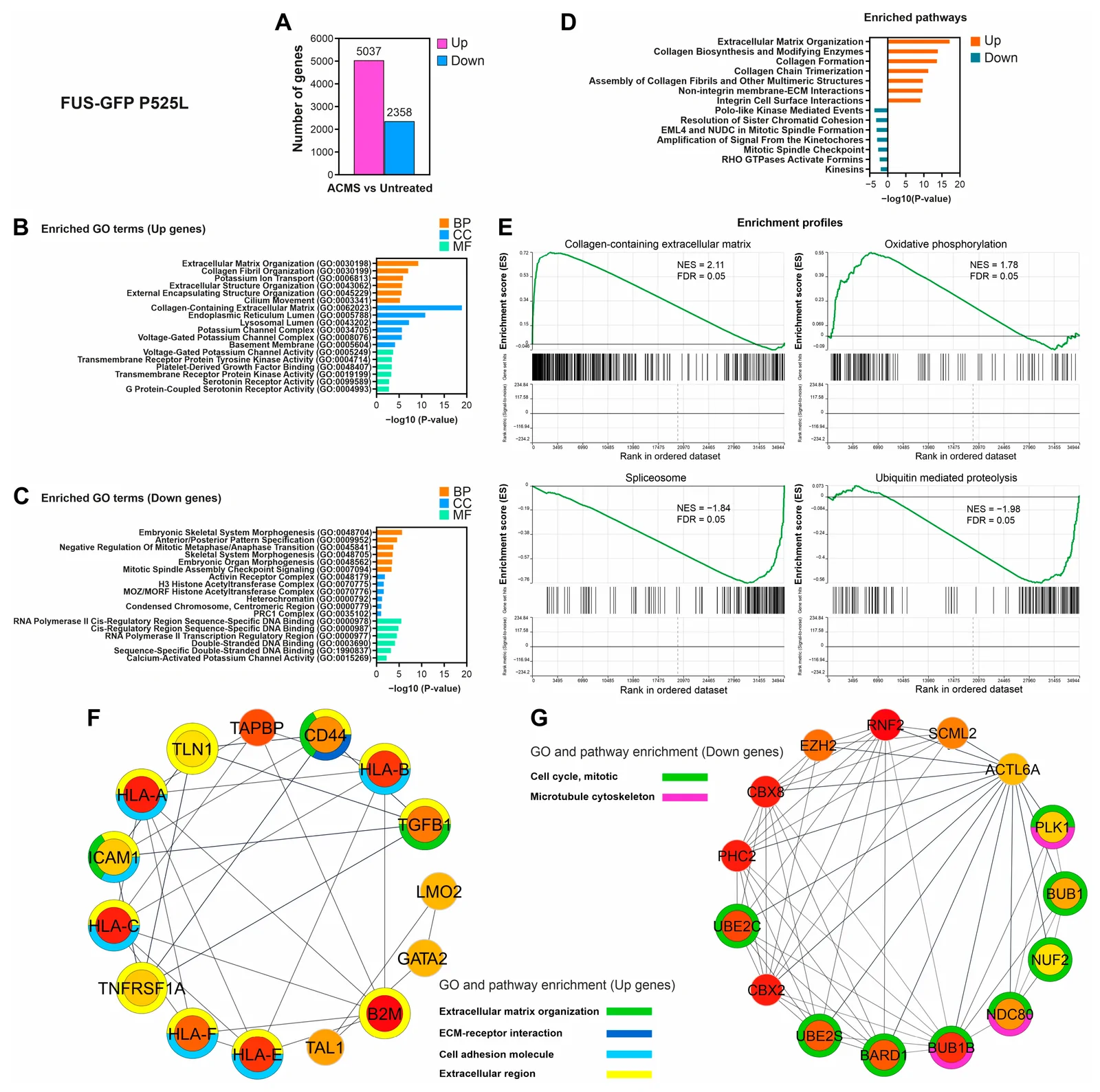
Functional enrichment analysis and PPI analysis of DEGs in isogenic FUS-GFP P525L mutant MNs. (**A**) Statistics of the results of differentially expressed genes (DEGs). (**B**–**D**) Functional analysis of enriched genes identified a variety of (**B**) upregulated and (**C**) downregulated GO terms and (**D**) enriched pathways (Reactome and KEGG) associated with FUS-GFP P525L MNs (*p* ≤ 0.05). Gene ontology (GO) term enrichment analysis of differentially expressed genes ranked the most enriched gene group changes in FUS-GFP P525L MNs (ACMS versus untreated). The enrichment analysis was performed by EnrichR, and significant terms/pathways were listed with an adjusted *p*-value ≤ 0.05. The GO terms were divided into three categories, including biological process (BP, orange), molecular function (MF, green), and cellular component (CC, blue), respectively. (**E**) Gene set enrichment analyses (GSEAs) were performed using normalized counts from the DESeq2 output ranked list in FUS-GFP P525L mutant datasets (ACMS versus untreated); *p* ≤ 0.05 and FDR ≤ 0.05 were considered statistically significant. The top two most significantly enriched gene sets are shown as positively (**top** row) and negatively (**bottom** row) correlated with the gene counts based on normalized enrichment score (NES) values. The *y*-axis represents enrichment scores (green curve) across the entire gene set for each individual gene member on the *x*-axis (vertical black bars) of that gene set. Top 15 (**F**) upregulated and (**G**) downregulated hub genes as deduced from the corresponding protein–protein interaction (PPI) networks (by STRING analysis) shown in Figure S17. Hub genes were identified by the maximal clique centrality (MCC) algorithm using Cytoscape (cytoHubba plugin). The darker the central color of each hub circle, the higher the gene rank, with a color progression from yellow to orange to red indicating increasing rank. In contrast, the peripheral color segments represent the GO terms and pathway enrichments associated with the respective hub genes, as indicated by the adjacent color legends. Some hub genes did not yield any significant enrichment results and are therefore devoid of peripheral color coding (e.g., TAL1 and EZH2).

**Figure 6:**
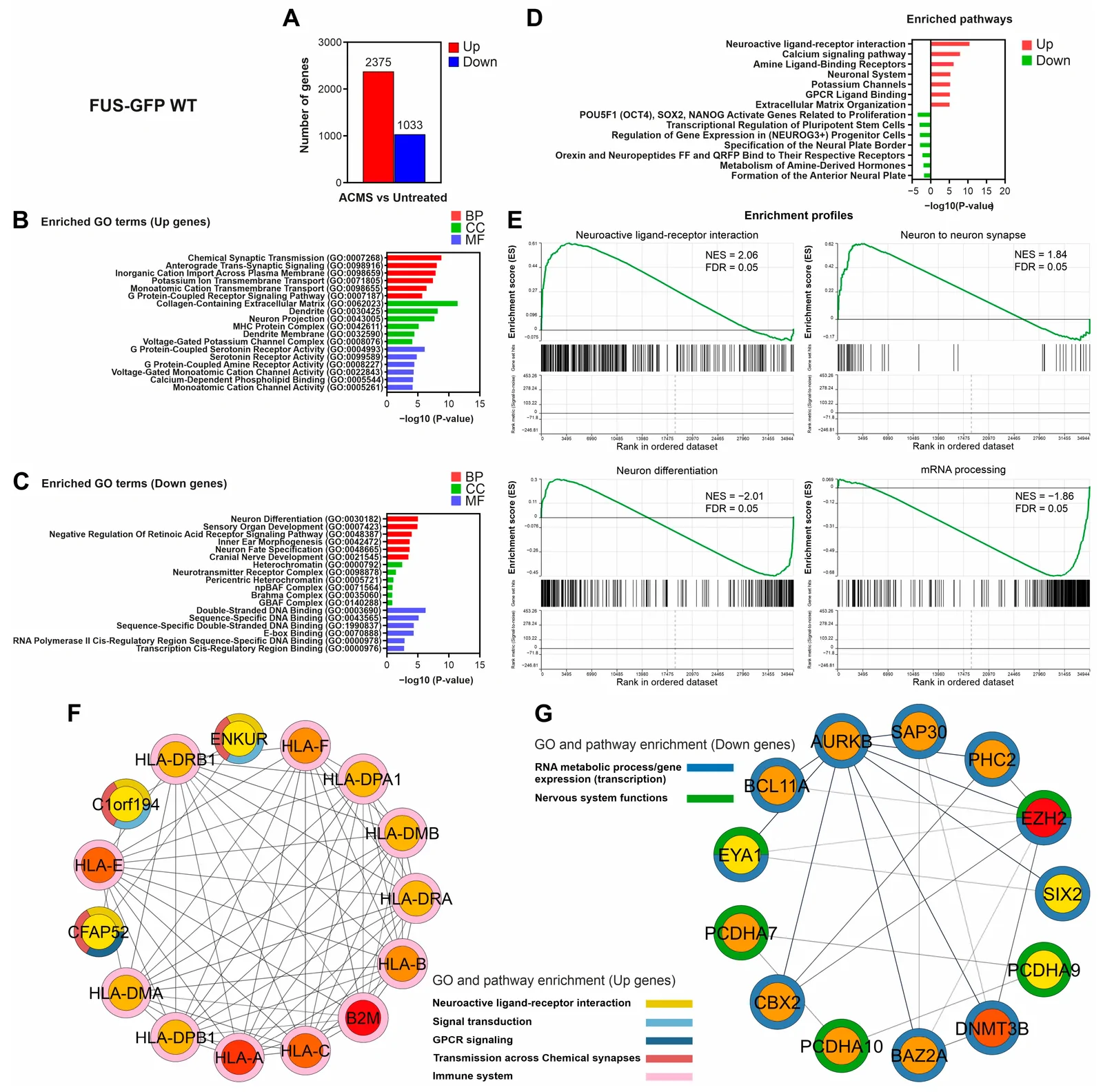
Functional enrichment analysis and PPI analysis of DEGs in isogenic FUS-GFP WT MNs. (**A**) Statistics of the results of DEGs. (**B**–**D**) Functional analysis of enriched genes identified a variety of (**B**) upregulated and (**C**) downregulated GO terms and (**D**) enriched pathways (Reactome and KEGG) associated with FUS-GFP WT MNs (*p* ≤ 0.05). Gene ontology (GO) term enrichment analysis of differentially expressed genes ranked the most enriched gene group changes in FUS-GFP WT MNs (ACMS versus untreated). The enrichment analysis was performed by EnrichR, and significant terms/pathways were listed with an adjusted *p*-value ≤ 0.05. The GO terms were divided into three categories, including biological process (BP, red), molecular function (MF, blue), and cellular component (CC, green), respectively. (**E**) GSEAs were performed using normalized counts from the DESeq2 output ranked list in FUS-GFP WT datasets (ACMS versus untreated); *p* ≤ 0.05 and FDR ≤ 0.05 were considered statistically significant. The top two most significantly enriched gene sets are shown that positively (**top** row) and negatively (**bottom** row) correlate with the gene counts based on NES values. The *y*-axis represents enrichment scores (green curve) across the entire gene set for each individual gene member on the *x*-axis (vertical black bars) of that gene set. Top 15 (**F**) upregulated and (**G**) downregulated hub genes as deduced from the corresponding protein–protein interaction (PPI) networks (by STRING analysis) shown in Figure S18. Hub genes were identified by the maximal clique centrality (MCC) algorithm using Cytoscape (cytoHubba plugin). The darker the central color of each hub circle, the higher the gene rank, with a color progression from yellow to orange to red indicating increasing rank. In contrast, the peripheral color segments represent the GO terms and pathway enrichments associated with the respective hub genes, as indicated by the adjacent color legends. Likewise, the number of DEGs identified in the untreated isogenic pair of mutant FUS-GFP P525L versus FUS-GFP WT MNs is shown in Figure S10. In the non-isogenic line pools of mutant ALS-FUS and WT Ctrl MNs (each ACMS versus untreated), using Venn diagrams, we overlapped DEGs in the three non-isogenic mutant ALS-FUS lines (Table 1) and identified 2544 commonly upregulated and 399 commonly downregulated DEGs for further analysis (Figure S11A,B). As for the three non-isogenic WT Ctrl lines, we overlapped 1646 commonly upregulated and 539 commonly downregulated DEGs for further analysis (Figure S12A,B). Gene expression levels (normalized short read counts) of top DEGs in the volcano plots (Figure 4D and Figure S8E–K) are presented as bar graphs for the isogenic pair (Figure S13A–C) as well as the non-isogenic Ctrl and mutant FUS line pools (Figure S13D,E).

Next, we expanded our analysis beyond the principal component space that was limited to the top 500 variable genes. Volcano plots showed the full distribution of gene expression across all FUS-GFP WT samples (ACMS versus untreated, Figure 4D top), likewise across all mutant FUS-GFP P525L samples (ACMS versus untreated, Figure 4D bottom), as well as between untreated mutant and control samples (FUS-GFP P525L versus FUS-GFP WT, Figure S8E). Differential expression analysis was performed in FUS-GFP P525L and FUS-GFP WT control samples to filter all obtained differentially expressed genes (DEGs) (Figure 4D and Figure S8E–K) based on the threshold of adjusted *p* ≤ 0.05, log2FC (Fold Change) ≥ 1 or ≤ −1 and a false discovery rate (FDR) of 5%. Among them, 5037 genes were upregulated, and 2358 genes were downregulated in mutant FUS-GFP P525L MNs (ACMS versus untreated, Figure 5A), whereas 2375 genes were upregulated and 1033 genes were downregulated in FUS-GFP WT MNs (ACMS versus untreated, Figure 6A).

As a targeted, hypothesis-driven complement to our global DEG analyses, we searched the complete DEG list (Table S1) for established and plausible candidate effectors that could potentially mediate the observed rescue of organelle motility and DDR as downstream components of the underlying causal pathway. We focused particularly on motor proteins, microtubule-associated proteins, mitochondrial maintenance, DDR, signal transduction, and transcriptional regulation. This complementary approach was motivated by the possibility that individual downstream effectors, despite potentially contributing substantially to the observed phenotypes, may be insufficient to yield significant enrichment in conventional GO or pathway analyses. We therefore expected functionally relevant candidate effectors to be shared across all eight lines (control and mutant; Table 1), consistent with common transcriptional drivers underlying the convergence observed in the principal component space (Figure 4A and Figure S9, Table S3). In addition, we considered key players showing ACMS-responsive changes specifically within either the control or mutant line pool (Table 1). Thus, we searched our full list of DEG read values (Table S1) for those candidates that were either commonly (i) up- or downregulated in all eight lines (Table 1) or (ii) commonly up- or downregulated only within the WT Ctrl pool (four lines, Table 1) or (iii) commonly up- or downregulated only within the mutant ALS-FUS pool (four lines, Table 1). Using stringent selection criteria of a low FDR cutoff (FDR ≤ 0.05), low adjusted *p*-value (Padj ≤ 0.05), and a log2FC threshold (log2FC ≥ 1 or ≤ −1), we curated a short list of common DEGs, separated by up- and downregulation (Table S4). Violin plots show the full distribution of expression levels (normalized read counts) for a specific gene across all sample replicates and treatment groups (untreated versus ACMS) rather than just a mean (Figure S14). These plots visually confirm that the statistical significance reported in Table S4 corresponds to a clear, consistent shift in gene expression between the untreated and ACMS-treated samples. We found that specific genes common to all eight lines (Ctrl and mutant, Table 1) centered on a coordinated response to ACMS treatment (Table S4, Figure S14) involving microtubule dynamics (e.g., upregulated *KIFC2*, *KIF13B*, *MAP7*, *MAPT/Tau*, and *EML6;* downregulated *KIF19*), metabolic/mitochondrial regulation (upregulated *PPARGC1A*, *PPARGC1B*), signal transduction/transcriptional activation (upregulated *STAT1*, *STAT3*, *STAT5A*) cellular stress and DNA damage response (upregulated *PARP9*, *PARP12*, *PARP14*, downregulated *PARP11*), as well as synaptic/membrane scaffolding (downregulated *UNC13B*). In addition, we found genes specifically responding to ACMS only within the mutant FUS pool (four lines, Table 1), involving upregulated *KIF24*, *EML1*, *UNC13C*, *DNMBP* (Table S4), thereby hinting at individual candidates in addition to shared transcriptomic features predominantly driving the convergence of profiles in the PCA plot (Figure 4A).

### 3.2. Extracellular Matrix (ECM) Components as Key Players in ACMS-Mediated Phenotypes

To characterize the biological processes and signaling pathways associated with the identified DEGs, we performed GO and pathway enrichment analyses (Reactome and KEGG; Figure 5B–D). In isogenic FUS-GFP P525L mutant MNs (ACMS versus untreated), upregulated DEGs were significantly enriched in ECM and collagen organization, with collagen-containing ECM prominent among cellular components (including *COL1A1*, *COL2A1*, *COL9A3*, *COL4A1*, *COL4A2*, *COL4A5*, *COL4A6*, *COL6A1*, and *COL6A2*; Table S5). Enriched molecular functions also included synapse-related terms (Figure 5B). Downregulated DEGs were associated with skeletal system morphogenesis, histone complexes, and RNA polymerase II activity (Figure 5C). Pathway analysis similarly highlighted ECM organization and collagen formation among upregulated genes, whereas downregulated pathways were associated with cell cycle events (Figure 5D). These findings implicate ECM remodeling in the ACMS-associated transcriptomic response, although its functional significance requires further investigation. Complete GO and pathway enrichment results are provided in Table S5.

Gene set enrichment analysis (GSEA) [54] corroborated these findings, identifying enrichment of collagen-containing ECM (normalized enrichment score [NES] = 2.11, FDR = 0.05) and oxidative phosphorylation (NES = 1.78, FDR = 0.05) among positively enriched gene sets. Conversely, spliceosome (NES = −1.84, FDR = 0.05) and ubiquitin-mediated proteolysis (NES = −1.98, FDR = 0.05) were negatively enriched (Figure 5E and Figure S15A,B, Table S6). Heatmaps of ‘leading-edge genes’ with the highest Z-scores further illustrated the differential expression of ECM-associated genes following ACMS treatment (Figure S16). Protein–protein interaction (PPI) analysis [58] identified interconnected upregulated hub genes associated predominantly with ECM organization and cell adhesion, whereas downregulated hubs were linked to cell cycle regulation and the microtubule cytoskeleton (Figure 5F,G, Figures S16 and S17A,B; Table S7). Together, these analyses reveal coordinated transcriptomic changes involving ECM organization, cellular adhesion, energy metabolism, and RNA processing in ACMS-treated FUS-GFP P525L MNs.

In isogenic FUS-GFP WT MNs, ACMS-associated upregulated DEGs were enriched in synaptic and neuronal functions, with cellular component terms including ECM, dendrites, and neuron projections, and molecular functions including voltage-gated channel and serotonin receptor activity (Figure 6B, Table S5). Downregulated DEGs were associated with neuronal development and fate specification, chromatin complexes, and DNA binding (Figure 6C). Pathway enrichment similarly highlighted neuronal functions among upregulated genes and transcriptional regulation among downregulated genes (Figure 6D). GSEA identified enrichment of neuronal synapse and neuroactive ligand–receptor interaction pathways, whereas negatively enriched gene sets were primarily associated with mRNA-related functions (Figure 6E and Figure S15C,D; Table S6). Heatmap clustering further indicated coordinated changes in gene sets related to oxidative phosphorylation, microtubule-based transport, ECM–receptor interactions, MAPK signaling, and nucleo-cytoplasmic transport (Figure S16). Consistent with these results, PPI analysis identified upregulated hub genes associated with neuroactive ligand–receptor interactions, signal transduction, and chemical synaptic transmission, while downregulated hubs were linked to mRNA metabolism and nervous system maintenance (Figure 6F,G and Figure S18A,B; Table S7). Thus, the WT response was characterized predominantly by neuronal and synaptic signatures, alongside changes in RNA- and ECM-related processes.

To distinguish ACMS-associated changes from baseline genotype differences, we also compared untreated isogenic FUS-GFP P525L and WT MNs (Figure S10). Upregulated DEGs in the mutant were enriched in muscle structure development, organ morphogenesis, calcium channel complexes, and transmembrane transporter activity (Figure S10B). Downregulated DEGs were associated with ECM-related processes and the external encapsulating structure, with enrichment of collagen-containing ECM, endoplasmic reticulum, tyrosine kinase activity, and growth factor binding (Figure S10C). Correspondingly, MAPK signaling and receptor-related pathways were enriched among upregulated genes, whereas ECM organization, ECM proteoglycans, and collagen formation predominated among downregulated pathways (Figure S10D). GSEA further indicated enrichment of DNA repair, cell cycle, and nucleo-cytoplasmic transport signatures, alongside negative enrichment of oxidative phosphorylation and ECM organization (Figures S10E and S19; Table S6). Heatmaps and PPI networks, including the inferred hub genes, were consistent with these enrichment patterns (Figures S10F,G, S16 and S20; Table S7).

### 3.3. ECM Components as Key Players of ACMS-Mediated Phenotypes in Non-Isogenic MNs as Well

GO enrichment analysis of pooled non-isogenic mutant ALS-FUS MN lines (ACMS versus untreated; Table 1; Figure S11A,B) revealed a prominent ECM-associated signature. Upregulated DEGs were enriched in ECM organization, collagen organization, and extracellular structure organization, with representative genes including *FMOD*, *S100A4*, *S100A6*, and *COL1A1* (Table S5). These genes have also been reported as dysregulated in ALS and other motor neuron diseases [59,60]. Collagen-containing ECM was the most prominent cellular component term, while enriched molecular functions included G-protein-coupled receptor (GPCR) activity and growth factor binding (Figure S11C). In contrast, downregulated DEGs were associated with transcriptional regulation, axon development, and nervous system development, with enrichment of the SWI/SNF complex, axon-related components, and DNA-binding functions (Figure S11D). Pathway analysis confirmed enrichment of ECM organization and collagen formation among upregulated genes, whereas downregulated genes were predominantly associated with gene-expression regulation (Figure S11E; Table S5).

GSEA likewise identified enrichment of ECM–receptor interactions and collagen-containing ECM, alongside negative enrichment of transcriptional and RNA-related processes (Figures S11F and S21A,B; Table S6). PPI analysis identified upregulated hub genes associated predominantly with ECM organization, ECM–receptor interactions, and cell adhesion molecules, whereas downregulated hubs were linked to nervous system development and RNA metabolism (Figures S11G,H and S22; Table S7). These expression patterns were, again, visualized in heatmaps (Figure S16). The convergence of GO, GSEA, and PPI results therefore identifies ECM remodeling as a prominent feature of the ACMS-associated response in non-isogenic FUS-mutant MNs, consistent with the findings in the isogenic FUS-GFP P525L model.

In pooled non-isogenic WT control MNs, ACMS-associated upregulated DEGs were enriched in ion transport and synaptic transmission, with collagen-containing ECM again represented among cellular component terms and transmembrane receptor activity among molecular functions (Figure S12C; Table S5). Downregulated DEGs were associated with nervous system development and transcriptional regulation, with microtubule cytoskeleton, spindle formation, and DNA-binding functions among the enriched terms (Figure S12D). Pathway analysis highlighted neuronal system functions and ECM organization among upregulated genes, whereas downregulated genes were associated with cell cycle regulation (Figure S12E). GSEA similarly identified enrichment of voltage-gated channel activity and chemical synaptic transmission, alongside negative enrichment of nervous system development and cell cycle-related gene sets (Figures S12F and S21C,D; Table S6). PPI analysis supported these findings, identifying upregulated hub genes associated with synaptic functions and downregulated hubs linked to nervous system development and cell cycle regulation (Figures S12G,H and S23; Table S7). Thus, the WT controls exhibited, similar to their isogenic counterpart, neuronal and synaptic transcriptional signatures alongside the common ECM-associated response to ACMS.

### 3.4. Relationship Between Global Differential Gene Expression and Principal Component Analysis

Together, the global DEG analyses provide a direct link between the transcriptional changes identified across the dataset and the principal component space, particularly through enrichment analysis of the gene loadings contributing most strongly to PC1 (Figure 4A and Figure S9, Table S3). Importantly, both analytical approaches independently converged on similar gene networks and biological pathways. Thus, the transcriptional programs associated with the ACMS-induced phenotypic changes in the global DEG analysis also emerged as major contributors to the PC1-associated convergence of transcriptomic profiles across genotypes. In particular, broad activation of ECM-associated pathways, accompanied by changes in transcriptional regulators and neurogenic programs, consistently emerged across both analyses (e.g., compare Figure S9 with Figure S21). This concordance supports the interpretation that ACMS induces a coherent, coordinated transcriptional program rather than isolated gene-level changes, thereby linking the observed phenotypic recovery to the broader transcriptomic state toward which both control and FUS-mutant MNs converge.

## 4. Discussion

Our optimized ACMS protocol induces global transcriptomic reprogramming associated with beneficial recovery processes in cultured MNs, warranting further investigation of its therapeutic potential in vivo and in clinical studies.

To our knowledge, no study has so far performed a comprehensive and defined scan of ACMS frequency, amplitude, spatial field orientation, and sustainability of impact in cultured neurons. Using axonal organelle motility as a primary functional readout, we identified a clear frequency optimum at 25 Hz (Figure 2A,B and Figure S3), suggesting a frequency-specific, resonance-like interaction indicative of a magneto-receptive mechanism of currently unknown molecular nature. In this regard, the moderate shift in the ACMS frequency optimum from 25 Hz in MNs to 50 Hz in HeLa cells is noteworthy, as it raises the possibility that different cell and tissue types may express distinct variants or isoforms of molecules that serve as field sensors. Identification and characterization of these postulated magnetosensors in follow-up studies will therefore be important for developing cell- and tissue-specific ACMS applications. In addition to frequency, spatial orientation of the magnetic field emerged as a critical determinant of efficacy. Field lines oriented perpendicular to the axonal plane were markedly more effective in enhancing organelle motility than parallel configurations (Figure 1A–E, Figure 2C and Figure S4). This finding suggests that the highly ordered, compartmentalized architecture of axons in MFCs may constrain or orient a putative magnetoreceptor relative to the field vector, raising the possibility that distal axons represent the predominant upstream site of field sensing. If transferable to the in vivo setting, this observation has direct implications for clinical device design: optimal stimulation may require positioning limbs between paired Helmholtz coils to achieve a perpendicular field orientation relative to motor axon bundles, whereas exposure within the bore of a single long solenoid, where the field is largely parallel to the axons, may be suboptimal. Moreover, our field-amplitude titration suggested a switch-like rather than dose-dependent biological response (Figure 2E and Figure S6). Although the 7 mT threshold is readily achievable in vitro, generating comparable fields at cortical, spinal, or peripheral MNs in vivo is more challenging because magnetic flux density B decreases rapidly with distance r from the solenoid (B[r] ∝ 1/r^3^) [61]. Thus, typical domestic devices are unlikely to deliver fields of this magnitude at the required tissue depth. The field strengths used here are technically achievable with appropriately scaled electromagnetic systems, but translation to whole-tissue or human applications will require dedicated engineering solutions addressing coil geometry, power delivery, heat dissipation, field homogeneity, and tissue penetration. Clinical systems such as MRI scanners demonstrate that these challenges can, in principle, be addressed through appropriate scaling, control, and cooling infrastructure. Importantly, however, such engineering challenges should be distinguished from a fundamental limitation of the therapeutic concept itself.

Previous work demonstrated that ALS-FUS MNs exhibit not only impaired distal axonal organelle trafficking but also a defective DDR [15,47]. Mutations in the FUS-NLS perturb nucleo-cytoplasmic shuttling and recruitment of FUS to nuclear DDSs, impairing activation of classical and alternative non-homologous end joining repair pathways [15]. Importantly, these two phenotypes are functionally linked: deficient DDR precedes and causally contributes to impaired axonal trafficking, as pharmacological inhibition of FUS recruitment to nuclear DDSs in control cells recapitulates the trafficking defect, whereas rescue of FUS recruitment to nuclear DDSs in mutant cells restores axonal motility [15,46]. These findings led to the concept of a nucleo-axonal crosstalk linking nuclear genome maintenance to distal axonal function [15,46,62,63].

In the present study, ACMS restored FUS recruitment to laser-induced DDSs in mutant MNs with the same frequency optimum of 25 Hz observed for axonal trafficking (Figure 3A–C vs. Figure 2A,B), accompanied by disappearance of cytosolic FUS aggregates indicative of improved nucleo-cytoplasmic shuttling (Figure 3G,H). These concordant effects across two mechanistically linked readouts support the notion that ACMS acts upstream of both, further reinforcing their functional coupling through nucleo-axonal crosstalk.

To gain insight into the mode of ACMS action, we compared two experimental paradigms: (i) our established protocol of four consecutive ACMS sessions followed by phenotypic assessment (Figure 1J), and (ii) acute magnetic field exposure during live imaging of previously untreated cells (Figure S7). The absence of immediate effects in the acute exposure paradigm argues against direct physical interactions with motor proteins or microtubules. In contrast, the persistence of hyper-physiological axonal motility for up to eight days after the final ACMS session (Figure 2D and Figure S5) strongly suggests an indirect mechanism mediated by lasting transcriptional or epigenetic changes. Such persistence is clinically attractive, as it implies that chronic continuous stimulation may not be required. However, the induction of a hyper-physiological state—characterized by increased velocity, straighter trajectories, reversal toward retrograde transport, and mobilization of previously stationary organelles (Figure S3B–E)—also raises caution regarding potential long-term overstimulation. We observed no overt signs of cellular stress such as axonal swellings or degeneration during the post-treatment chase after repeated ACMS sessions (Figure 1J) either in movies (e.g., Figure S5) or fixed-cell images (e.g., Figure S24). Apoptosis was even downregulated in some lines (Figures S15A and S21C). The benign outcome of our ACMS protocol may be related to the resting intervals between the consecutive ACMS sessions (Figure 1J). Nevertheless, translation of the 25 Hz frequency optimized here in vitro requires careful assessment of tolerable field exposure, duration, and repetition rate. We plan to address these parameters in vivo using genetic ALS mouse models subjected to long-term ACMS. An interesting possibility is to test frequencies that were less effective in vitro (e.g., 10 or 50 Hz; Figure S3) but restored deficient organelle motility more closely to wild-type levels without exceeding hypermobility, while still mediating moderate stimulation of DDR (Figure 3B,C). Establishing such parameters will be crucial for balancing biological efficacy and safety in future clinical applications.

From a technical perspective, the close clustering of individual lines and replicate samples in the PCA plot (Figure 4A) indicated high experimental consistency and supported the spatial and temporal homogeneity of temperature and magnetic field generated by the water-cooled solenoids during ACMS. Our sample holders precisely centered the MFCs along the longitudinal axis within the central bore, where deviations from parallel field lines toward the edges (Figure 1A,B) were negligible. Moreover, ad hoc activation of the solenoid enclosing the cells directly during live imaging (Figure S7, Movie S7) produced neither visible blur or jitter nor any detectable change in organelle motility throughout the recordings. Together, these observations argue against vibrational or thermal artifacts as contributors to the biological effects of ACMS. Consistently, frequencies far from the optimum (2 and 200 Hz; Figure 2B and Figure S3) and a parallel rather than perpendicular field orientation (Figure 2C and Figure S4) failed to reproduce the effects observed at 25 Hz.

Our global RNA-seq analysis revealed three distinct and internally consistent transcriptomic states by PCA: untreated controls, untreated mutants, and a single unified state encompassing all eight ACMS-treated lines (Table 1), regardless of genotype (Figure 4A). Remarkably, these three transcriptional states corresponded closely to the three observed functional states of axonal organelle motility (Figure 4B). This finding suggests that ACMS drives MNs into a discrete, system-wide transcriptional program associated with a hyper-physiological functional state rather than merely correcting mutant-specific defects. This view is further supported by identical gene sets driving the predominant convergence of all samples along the PC1-axis in the principal component space across all WT and mutant genotypes (Figure 4A and Figure S9, Table S3). Notably, the same hyper-physiological state was observed across all wild-type control lines, suggesting that the beneficial effects of ACMS may extend beyond FUS-NLS mutations to other ALS genotypes and potentially to broader neurodegenerative applications. Deficient axonal organelle motility and increased DNA damage accumulation have also been implicated in several other neurodegenerative diseases [64,65,66,67,68]. Follow-up studies using additional genetic and sporadic disease models will therefore be required to explore this broader therapeutic potential while also assessing potential adverse effects. Moreover, the abrupt loss of the hyper-physiological state after eight days (Figure 2D and Figure S5) and the sharp field-strength threshold (Figure 2E and Figure S6), reminiscent of global cellular stress responses such as heat-shock signaling [65,66], support a switch-like nature of the cellular response to ACMS [69,70].

### 4.1. Limitations of the Present Study and Outlook

Regarding the pathways underlying ACMS-mediated alterations, our RNA-seq analysis identified a remarkably large number of DEGs, with approximately 7400 DEGs in FUS-GFP P525L MNs alone (Figure 5A). This extensive transcriptomic remodeling generates a complex multitude of candidate pathways across GO, KEGG, GSEA, and other enrichment analyses, thereby hampering the identification and validation of key players from the global dataset alone. We therefore distinguish clearly between conclusions supported by direct experimental evidence and hypotheses derived from DEG enrichment analyses.

On the former, our study provides a proof of concept by demonstrating favorable phenotypic improvements associated with a tightly defined, novel and unified transcriptomic state induced and sustained by appropriately tuned ACMS parameters, rather than by direct acute interactions with downstream effectors. On the latter, we consider a systematic, spatially resolved strategy more informative than selecting a limited number of candidates primarily on the basis of feasibility from the vast global dataset. We therefore plan to refine our compartmentalized MFC cultures to separately analyze the transcriptomes of distal axons and the somatodendritic compartment. In parallel, we are developing custom hardware for spatially restricted ACMS using sharply focused magnetic fields [71]. This approach will allow us to test our conceptual model that distal axons constitute the primary site of the putative magnetosensitive mechanism. The combined spatial resolution of transcriptomics and ACMS will provide an important basis for candidate selection by distinguishing transcriptomic changes associated specifically with the distal axonal compartment, where we hypothesize that the primary ACMS-induced modulation of organelle trafficking occurs [72,73]. This should substantially narrow the pool of biologically relevant candidates and enable more focused and mechanistically informative validation. We therefore view these spatially resolved studies not as a postponement of validation, but as a necessary refinement to make subsequent validation more hypothesis-driven and informative. Once prioritized candidates have been identified, follow-up studies will combine orthogonal and protein-level validation, including Western blotting and immunostaining, with functional RNAi or genetic knockout approaches.

### 4.2. Toward a Conceptual Model

To provide a further framework for the forthcoming validation experiments, we sought to derive a conceptual model from the present global DEG analyses. GSEA consistently highlighted upregulation of ECM components, collagen-related pathways, and associated receptors across all lines (Figure 5B,D,E, Figure 6B,D, Figures S11C,E,F, S12C,E, S15A,C,D, S16C and S21A,C). These changes, together with enriched protein–protein interaction hubs in mutant MNs (Figure 5F), suggest activation of regenerative adhesion and differentiation programs that may counteract neurodegenerative processes. This interpretation aligns with the downregulation of ECM components observed in untreated mutant MNs (Figure S10C,D,E,G) [41]. Transcriptomic studies in SOD1-G93A and C9ORF72 mouse models have likewise implicated ECM components in disease onset and progression, potentially across different genetic causes [59,60,74]. Recent studies in patients with sporadic ALS have further identified synaptic function, immune responses, and ECM/cytoskeleton pathways among the major dysregulated processes, highlighting ECM-related genes as potential ALS biomarkers [75,76,77]. The restoration of ECM-related gene expression in our FUS-mutant MNs (Figure S10C,D,E,G) may therefore reflect reactivation of regenerative neuronal programs, reminiscent of responses reported for magnetic stimulation in bone and wound healing [23,27,28] and mesenchymal stem cell differentiation [30]. This raises the possibility of shared magnetosensitive mechanisms promoting differentiation-related gene expression across various tissue types. However, this interpretation remains speculative, albeit plausible, as it cannot be reliably established from the present data.

Several findings require particular clarification in future studies. Most notably, robust induction of GFAP across all lines (Figure S13A,B,D,E) occurred together with significant downregulation of the transcription factor NEUROD4 (Figure 4D, Figures S8F–K and S13B,E), yet their relationship to restored axonal organelle motility and DDR remains unclear. Because the absence of glial contamination is generally considered an indicator of successful neuronal differentiation in iPSC-derived neurons, GFAP induction at later stages, when the neurons were already mature and postmitotic, was unexpected. At first glance, this could suggest partial reversal of neuronal differentiation or residual NPCs undergoing glial differentiation. However, such interpretations are difficult to reconcile with the observed hyper-physiological organelle motility in the distal axonal compartment, which is not accessible to dendrites or glial protrusions [15]. Importantly, GFAP-positive signals were absent from the distal axonal compartment (Figure S24), irrespective of genotype (FUS-GFP WT versus P525L) or treatment (untreated versus ACMS). In the somatodendritic compartment, however, ACMS induced GFAP-positive punctate signals that appeared localized to the perinuclear cytoplasm. These signals did not resemble the characteristic GFAP filaments observed in the U87 MG positive control (Figure S24). Their identity, subcellular localization, and biological significance therefore remain unclear and require further investigation. The overall pattern of neurofilament staining (SMI32) was not detectably altered by ACMS in either the distal axonal or proximal somatodendritic compartment, arguing against a major change in neuronal differentiation status following ACMS.

As for other pathways, numerous neurotransmitter receptors, ion channels, and downstream kinase pathways (TNF, Toll-like, MAPK, JAK–STAT, NF-κB, PI3K–AKT) were upregulated (Figures S15 and S21), suggesting broad activation of receptor-mediated signaling. Downregulated DEGs were enriched for transcriptional regulation, chromatin remodeling, and RNA processing (Figure 5C, Figure 6C, Figures S11D and S12D), coinciding with uniform upregulation of STAT transcription factors—particularly STAT3 (Figure S14, Table S4)—consistent with the consolidation into the novel, unified transcriptional state (Figure 4A). Consistent with restored axonal transport, genes associated with oxidative phosphorylation, mitochondrial function (Figures S15A,C, S16A and S21A), and microtubule-based transport (Table S4, Figures S14, S16B and S21A,C) were upregulated. This included *PPARGC1A* and *B*, transcriptional coactivators involved in mitochondrial biogenesis (Figure S14, Table S4), as well as genes associated with microtubule stabilization, including *MAP7* and *MAPT* (*Tau*), and several kinesins (Figure S14, Table S4). Notably, uniform upregulation of KIFC2 [78] together with downregulation of KIF19 (Figure S14, Table S4) [79,80] across all eight lines (Table 1) provides a potential molecular correlate of the observed shift in net transport direction (Figure S3E).

Regarding DDR, FUS requires PARP-dependent post-translational modification, and inhibition of PARP1/2 in control cells mimicked the impaired DDR of the FUS-NLS mutant, whereas inhibition of the PARP antagonist poly(ADP-ribose) glycohydrolase (PARG) restored DDR in the mutant and concomitantly alleviated impaired nucleo-cytoplasmic shuttling and cytoplasmic aggregation [15]. Thus, the restoration of DDR by ACMS may be related to the coordinated transcriptional modulation of PARP family members observed across all eight lines (Table 1): PARP9, PARP12, and PARP14 were upregulated, whereas PARP11 was downregulated (Figure S14, Table S4). This pattern could potentially rebalance PARylation dynamics relevant to FUS recruitment to DDSs, although this interpretation requires direct experimental validation.

Finally, ACMS modulated several genes previously implicated in ALS therapeutic strategies, including mild downregulation of *ROCK1* (Table S4) across all eight lines (Table 1), of interest in light of recent clinical testing of the ROCK inhibitor Fasudil [81], and differential regulation of UNC13 isoforms. UNC13C was strongly upregulated in the mutant FUS line pool, whereas UNC13B was downregulated across all eight lines (Table 1; Table S4). Therapeutic strategies targeting reduced functional UNC13A levels are being investigated in certain ALS variants [82], and our findings raise the possibility of functional redundancy or compensatory interactions among UNC13 isoforms.

### 4.3. Summary

Taken together, our DEG analyses suggest a stepwise conceptual model in which ACMS is sensed—potentially at compartmentalized, aligned axons in MFCs—by cryptochromes or other transmembrane receptors [20,25]. The alternating magnetic field may interact in an orientation-dependent manner with radical pairs or dipoles associated with these proteins, thereby modulating their voltage-gating or ligand-binding properties [20]. Resulting ion fluxes and G-protein-coupled signaling could initiate cascades that propagate to the soma and induce coherent transcriptional reprogramming, ultimately modulating downstream effectors involved in cytoskeletal stabilization, motor protein function, ECM-mediated regeneration, nucleo-cytoplasmic shuttling, and DDR. This coordinated response could account for the observed hyper-physiological organelle motility and restoration of DDR. However, this model remains speculative, albeit plausible, as it cannot be reliably established from the present data. Nevertheless, it provides a conceptual framework for the hypothesis-driven refinement of forthcoming validation experiments.

Our ACMS protocol consistently restored axonal organelle motility and DDR, with downstream effectors that appear unexpectedly well predicted by the transcriptomic analysis. This may point to a pre-existing endogenous transcriptional program balancing neurodegenerative and regenerative processes, which ACMS could shift toward repair. Potential adverse or off-target effects, however, remain to be investigated.

## 5. Conclusions

Optimized ACMS at 25 Hz imposes a robust, genotype-independent hyper-physiological state upon human MNs in vitro, forcibly driving enhanced, redirected axonal organelle motility and reversing deficient DDR in ALS-FUS mutants. This phenotypic shift is associated with a novel, unified transcriptomic signature in both mutant and control lines. Ultimately, ACMS mediates a coordinated program, addressing the critical failure points of axonal trafficking and genome maintenance in neurodegeneration. Our findings propose ACMS as a novel, biophysical intervention with potential far beyond ALS, given the central role of axonal transport and genome maintenance in neurodegeneration.

## Figures and Tables

**Table 1:** Cell line and human donor characteristics.

Genotype	Cell Line	Sex	Age at Biopsy	Age at Disease Onset	Disease Duration (Months)	Clinical Phenotype	Mutation	Primarily Characterized in
WT	Ctrl1	male	48	-	-	-	-	[42]
WT	Ctrl2	female	43	-	-	-	-	[43]
WT	Ctrl3	female	48	-	-	-	-	[43]
IGC	FUS-eGFP^het^ WT alias FUS-GFP WT	isogenic to FUS R521C and FUS-GFP P525L	N/A	N/A	N/A	N/A	-	[15]
MT	FUS R521C^het^	female	58	57	7	Spinal	R521C	[15]
MT	FUS R521L^het^	female	65	61	60	Spinal	R521L	[15]
MT	FUS R495X^het^	male	29	no data	no data	Spinal	R495QfsX527	[15]
MT	FUS-eGFP^het^ P525L alias FUS-GFP P525L	isogenic to FUS R521C and FUS-WT GFP	N/A	N/A	N/A	N/A	P525L	[15]
WT	HeLa BAC FUS-eGFP WT	female	N/A	N/A	N/A	N/A	-	[44,45]
MT	HeLa BAC FUS-eGFP P525L	female	N/A	N/A	N/A	N/A	P525L	[44,45]

## Data Availability

RNA-seq data are publicly deposited at NCBI/GEO under the accession number GSE331298.

## Supplementary Materials

The following supporting information can be downloaded at: https://www.mdpi.com/article/10.3390/cells15212003/s1, Figure S1: Illustration of various waveforms and pulses for ACMS and PEMF and their respective harmonic underlying frequency spectra; Figure S2: Impact of ACMS on the speed distribution of the entire axonal organelle population at different frequencies; Figure S3: Rescue of axonal organelle motility depends on the ACMS frequency with an optimum at 25 Hz; Figure S4: Rescue of axonal organelle motility is more efficient at perpendicular orientation of the magnetic field lines with respect to the axonal plane; Figure S5: Rescue of axonal organelle motility is sustained for 8 days beyond the final ACMS session; Figure S6: Rescue of axonal organelle motility requires a field strength of ACMS above a critical threshold of 7 mT; Figure S7: ACMS-mediated sustained rescue of axonal organelle motility is due to slow, systemic underlying alterations in the cell rather than ad hoc interactions at moving organelles; Figure S8: Analysis of all ALS-FUS and Ctrl MN samples on normalized expression values of expressed genes; Figure S9: Upregulation of ECM pathways are the main drivers toward the novel, unified transcriptomic profile across all wild-type Ctrl and mutant FUS lines (Table 1) upon ACMS; Figure S10: Functional enrichment analysis and PPI analysis of DEGs in the untreated isogenic pair of FUS-GFP P525L versus FUS-GFP WT MNs; Figure S11: Functional enrichment analysis and PPI analysis of DEGs in the three lines of non-isogenic mutant FUS MNs; Figure S12: Functional enrichment analysis and PPI analysis of DEGs in the three lines of non-isogenic WT Ctrl MNs; Figure S13: Top DEGs in all cell lines; Figure S14: Scatter plot analysis of selected candidates from Table S4 common across all datasets; Figure S15: Gene set enrichment analysis (GSEA) ACMS versus untreated in the isogenic pair of FUS-GFP P525L and FUS-GFP WT; Figure S16: GSEA leading edge gene analysis; Figure S17: Refers to Figure 5F,G; Figure S18: Refers to Figure 6F,G; Figure S19: Gene set enrichment analysis (GSEA) in the untreated isogenic pair of FUS-GFP P525L versus FUS-GFP WT; Figure S20: Refers to Figure S10F,G; Figure S21: Gene set enrichment analysis (GSEA) ACMS versus untreated in the non-isogenic pools of mutant FUS and WT Ctrl lines; Figure S22: Refers to Figure S11G,H; Figure S23: Refers to Figure S12G,H; Figure S24: Induction of GFAP gene expression through ACMS in iPSC-derived spinal MNs leads apparently to no formation of cytoskeletal GFAP-positive filaments and to no cell fate change; Table S1: full DEG results from all eight lines (iPSC-derived MNs, Table 1), each ACMS versus untreated, as well as isogenic FUS-GFP P525L versus FUS-GFP WT, both untreated; Table S2: unfiltered ranked DEG lists of all eight lines (iPSC-derived MNs, Table 1), each ACMS versus untreated, as well as isogenic FUS-GFP P525L versus FUS-GFP WT, both untreated; Table S3: GO and Reactome term analyses of top 500 absolute gene loadings on PC1 of the PCA plot in Figure 4A, separated by positive and negative loadings; Table S4: short list of key DEGs common in all lines, separated by up- and downregulated candidates; Table S5: GO and Pathway term analyses results of the DEGs from isogenic FUS-GFP P525L and FUS-GFP WT (Table 1) MNs (each ACMS versus untreated, Figure 5B–D and Figure 6B–D) and FUS-GFP P525L versus FUS-GFP WT (both untreated, Figure S10B–D) as well as of the common DEGs (Venn diagrams, Figures S11A,B and S12A,B) from the non-isogenic pools of three mutant ALS-FUS (Table 1, Figure S11C–E) and three Ctrl lines (Table 1, Figure S12C–E), each ACMS versus untreated; Table S6: GSEA analysis results for normalized gene counts from isogenic FUS-GFP P525L and FUS-GFP WT (Table 1) MNs (each ACMS versus untreated, Figure 5E, Figure 6E and Figure S14) and FUS-GFP P525L versus FUS-GFP WT (both untreated, Figures S10E and S19) as well as from pooled datasets of the three non-isogenic mutant ALS-FUS (Table 1, Figures S11F and S21A,B) and three non-isogenic Ctrl lines (Table 1, Figures S12F and S21C,D), each ACMS versus untreated; Table S7: PPI analysis results of the DEGs from isogenic FUS-GFP P525L and FUS-GFP WT (Table 1) MNs (each ACMS versus untreated, Figures S17 and S18) and FUS-GFP P525L versus FUS-GFP WT (both untreated, Figure S20) as well as of the common DEGs (Venn diagrams, Figures S11A,B and S12A,B) from the non-isogenic pools of three mutant ALS-FUS (Table 1, Figure S22) and three Ctrl lines (Table 1, Figure S23), each ACMS versus untreated; Movie S1: Refers to the corresponding maximum intensity projections in Figure 2A and Figure S3A and documents the rescue of axonal organelle motility in mutant FUS MNs depending on ACMS frequency with an optimum at 25 Hz at otherwise 10 mT and perpendicular field orientation as illustrated in Figure 1A; Movie S2: Refers to the corresponding maximum intensity projections in Figure S4A and documents the more efficient rescue of axonal organelle motility in mutant FUS MNs at perpendicular as compared to parallel field orientation as illustrated in Figure 1A at otherwise optimal 25 Hz and 10 mT; Movie S3: Refers to the corresponding maximum intensity projections in Figure S5A and documents the sustained rescue of axonal organelle motility in mutant FUS MNs over at least eight days after the ACMS treatments at optimal 25 Hz and perpendicular field orientation at 10 mT as illustrated in Figure 1J; Movie S4: Refers to the corresponding maximum intensity projections in Figure S6A and documents the critical threshold of field strength around 7 mT in the ACMS treatments (Figure 1J) at otherwise optimal 25 Hz and perpendicular field orientation to achieve the sustained rescue of axonal organelle motility in mutant FUS MNs; Movie S5: Refers to the corresponding image gallery at 150 s in Figure 3A and documents the rescue of nuclear DDR after laser irradiation, i.e., the recruitment-dissociation cycle of FUS-GFP at laser cuts, in mutant FUS MNs (FUS-GFP P525L, Table 1) depending on ACMS frequency (Figure 1J) with an optimum at 25 Hz at otherwise 10 mT and perpendicular field orientation as illustrated in Figure 1A; Movie S6: Same as Movie S5 but for the ALS BAC HeLa cell model stably expressing FUS-GFP P525L (Table 1). Refers to the corresponding image gallery at 150 s in Figure 3D; Movie S7: Refers to Figure S7C and documents no ad hoc effect of ACMS during the movie recording after 10 min in mutant FUS MNs at optimal 25 Hz and 10 mT.

## Abbreviations

The following abbreviations are used in this manuscript:

A	adenine nucleotide
AC	alternating current
ACMS	AC magnetic stimulation
ADP	adenosine diphosphate
ALS	amyotrophic lateral sclerosis
a-NHEJ	alternative non-homologous end joining (in DNA double-strand break repair)
ATP	adenosine triphosphate
B	symbol for magnetic flux density
BAC	bacterial artificial chromosome
BGN	biglycan
bp	(nucleotide) base pairs
BP	biological process
C	cytosine nucleotide
C9ORF72	chromosome 9 open reading frame 72
CC	cellular component
CD44	cell-surface glycoprotein receptor, alias chondroitin sulfate proteoglycan 8
c-NHEJ	canonical non-homologous end joining (in DNA sdouble-strand break repair)
COL	collagen (several isoforms)
CTRL	control cells, typically wild type, gene-corrected or from healthy probands
DC	direct current
DCN	decorin
DDR	DNA damage response
DDS	DNA damage site (after Laser irradiation)
DEFRGs	differentially expressed and functionally related genes (in PPI analysis)
DEG	differentially expressed gene
DIV	days in vitro
DNA	deoxyribonucleic acid
DNMBP	dynamin binding protein
ECM	extracellular matrix
eGFP	enhanced green fluorescent protein
EML6	EMAP-like 6, echinoderm microtubule associated protein like 6
EMS	electromagnetic stimulation
ER	endoplasmic reticulum
fALS	familiar ALS
FC	fold change
FDR	false discovery rate
FFT	fast Fourier transform
FMOD	fibromodulin
fps	frames per second
FUS	fused in sarcoma
G	guanine nucleotide
GC	gene-corrected, i.e., a mutation reverted to wild type
GFAP	glial fibrillary acidic protein
GFP	alias of eGFP
GO	gene ontology
GPCR	G-protein–coupled receptor
GSEA	gene set enrichment analysis
Hz	Hertz
IGC	isogenic and gene-corrected
iPSC	inducible pluripotent stem cell
ITGA2	integrin subunit alpha 2
ITGB	integrin subunit beta (several isoforms)
JAK–STAT	janus kinases-signal transducer and activator of transcription
KEGG	Kyoto encyclopedia of genes and genomes
KIF	kinesin family (several isoforms)
LAMA	laminin subunit alpha (several isoforms)
LAMB	laminin subunit beta (several isoforms)
LUM	lumican
LUT	look-up table (for pseudocoloring of monochromatic images)
MAP	microtubule-associated protein (several isoforms)
MAPK	mitogen-activated protein kinase
MAPT	microtubule-associated protein tau
MF	molecular function
MFC	microfluidic chamber
MN	motor neuron
mRNA	messenger RNA
mT	millitesla
MT	mutant
N/A	not applicable
NA	numerical aperture
NEB	New England Biolabs^®^
NES	normalized enrichment score
NEUROD4	neurogenic differentiation 4
NF-H	neurofilament heavy chain
NF-κB	nuclear factor kappa-light-chain-enhancer of activated B cells
NGS	next-generation sequencing
NLS	nuclear location sequence
NPC	neuronal precursor/progenitor cell
OXPHOS	oxidative phosphorylation
P525L	point mutation in the carboxyterminal NLS of FUS protein with exchange of proline by leucine at position 525
PAR	poly(ADP-ribose)
PARG	poly(ADP-ribose) glycohydrolase, antagonist of PARP (several isoforms)
PARP	poly(ADP-ribose) polymerase (several isoforms)
PAX	paired box (transcription factor, several isoforms)
PC1	principal component 1
PC2	principal component 2
PCA	principal component analysis
PCR	polymerase chain reaction
PEMF	pulsed electromagnetic field
PI3K-AKT	phosphatidylinositol 3-kinase—protein kinase B (PKB), the latter is an alias of the name “AKT” which does not refer to its function. The “Ak” in Akt refers to the AKR mouse strain that develops spontaneous thymic lymphomas. The “t” stands for ‘thymoma’; the letter was added when a transforming retrovirus was isolated from the Ak mouse strain, which was termed “Akt-8”. The authors state, “Stock A Strain k AKR mouse originally inbred in the laboratory of Dr. C. P. Rhoads by K. B. Rhoads at the Rockefeller Institute.” When the oncogene encoded in this virus was discovered, it was termed v-Akt. Thus, the more recently identified human analogs were named accordingly.
PMEL	premelanosome protein
poly-A	poly-adenine nucleotides
poly-dT	poly-desoxythymidine nucleotides
PPARGC	peroxisome proliferator-activated receptor gamma, coactivator (several isoforms)
PPI	protein–protein interaction
r	symbol for distance (e.g., between solenoid and neurons)
R521L	point mutation in the carboxyterminal NLS of FUS protein with exchange of arginine by leucine at position 521
RIN	RNA integrity number
RNA	ribonucleic acid
RNAi	RNA interference
RNA-seq	RNA sequencing, see also NGS
ROBO3	roundabout guidance receptor 3
ROCK	Rho-associated coiled-coil containing protein kinase (several isoforms)
rTMS	repetitive TMS
S100A4	S100 calcium binding protein A4 (and other isoforms)
Seq	sequencing/sequence
SERPINE1	serpin family e member 1
SMI32	a non-phosphorylated epitope on NF-H, a common MN marker
SOD1	superoxide dismutase 1
SOD1-G93A	point mutation in the SOD protein with exchange of glycine by alanine at position 93
SPARC	secreted protein acidic and cysteine rich
STAT	signal transducer and activator of transcription (several isoforms)
STRING	Search Tool for the Retrieval of Interacting Genes/Proteins)
SWI/SNF	SWItch/sucrose non-fermentable, a subfamily of ATP-dependent chromatin remodeling complexes
T	thymidine nucleotide
TDP43	transactive response (TAR) deoxyribonucleic acid (DNA) binding protein 43 (TAR-DNA-binding protein-43)
TFAP2A	transcription factor activator protein (AP)-2 alpha
TMS	transcranial magnetic stimulation
TNC	tenascin C
TNF	tumor necrosis factor
Toll-like	Toll-like receptors (TLR), nicknamed after the German exclamation “Das ist ja toll!” when the first gene of this family was originally discovered in *Drosophila melanogaster* by German researcher Christiane Nüsslein-Volhard. The German term “toll” translates to “great”, “super”, “ace”, “wonderful”, etc.
TUBB3	βIII-tubulin
UNC	uncoordinated, referring to the uncoordinated movements in *Caenorhabditis elegans* caused by mutations in the UNC genes (several isoforms)
UT	untreated
VCAM1	vascular cell adhesion molecule 1
WT	wild type
XP	extremely pure
