# Supplementary material for: Electromagnetic Modulation of the ALS Transcriptome: Toward a Non-Drug Therapeutic Strategy
Source: Cells. 2026 Nov 3;15(21):2003. doi: 10.3390/cells15212003 (PMC13761629; doi:10.3390/cells15212003)
Supplement: Supplementary file 1 [file cells-15-02003-s001.zip › Supplement_revision1-cells4487635.pdf]

Supplemental materials of

Electromagnetic modulation of the ALS transcriptome: towards a non-drug therapeutic strategy

Arun Pal, Banaja P. Dash, Marc Uhlarz, Jonas Gronemann, Julia Japtok, Hannes Glaß, Jens Pietzsch, Joachim Wosnitza, Katrin Sameith, Andreas Dahl, Andreas Hermann, Richard H. W. Funk and Thomas Herrmannsdörfer

Table of contents

This supplemental section comprises

1. Figures S1-24 with captions
2. Tables S1-7 with captions
3. Movies S1-7 with captions

1. Figures S1-24

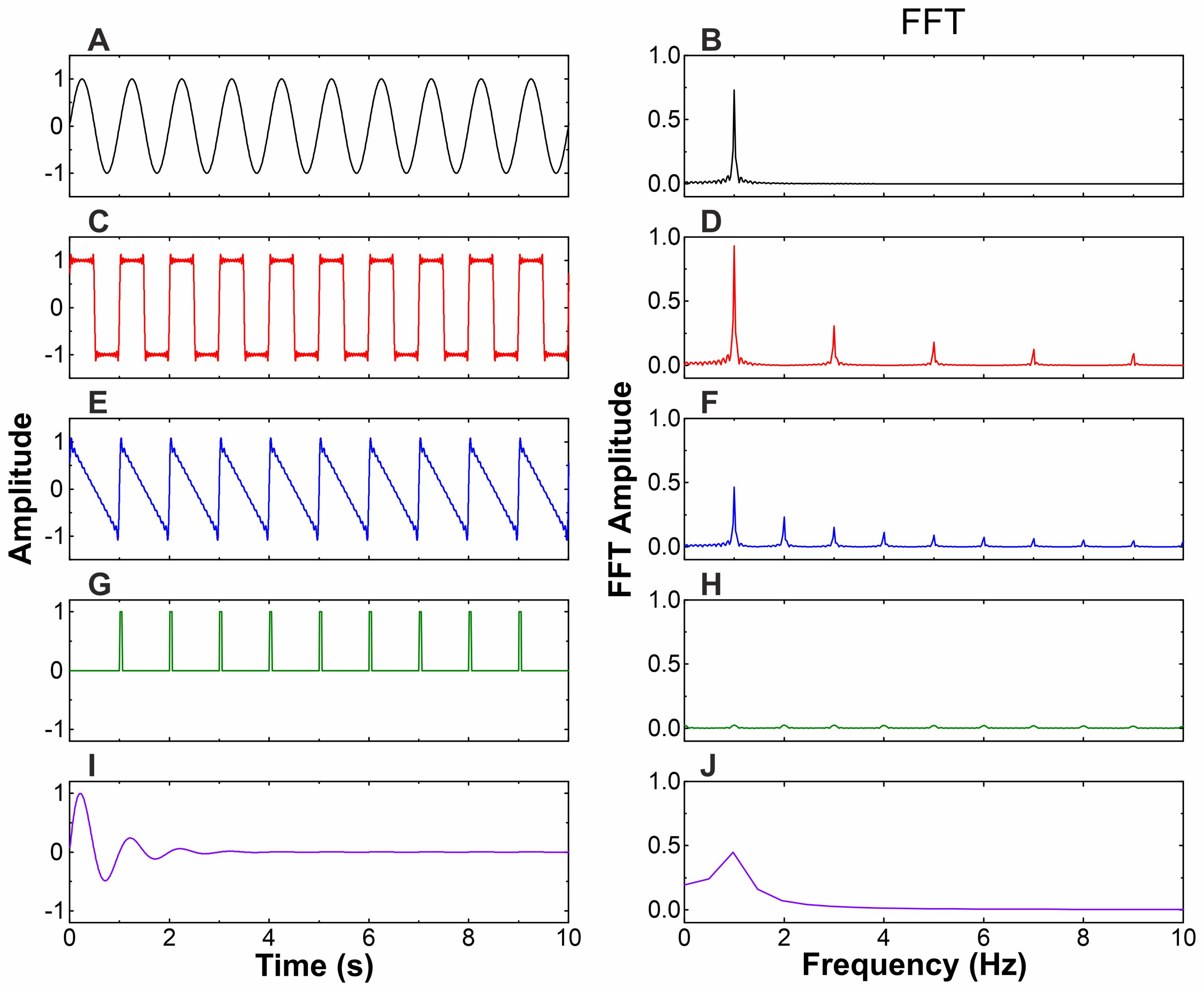

FFT
A
B
C
D
E
F
G
H
I
J
Amplitude
FFT Amplitude
Time (s)
Frequency (Hz)

Figure S1: Illustration of various waveforms and pulses for ACMS and PEMF and their respective harmonic underlying frequency spectra. On the left are shown the primary waves oscillating continuously at a hypothetical frequency of 1 Hz (**A, C, E**), a train of pulses at a hypothetical repetition rate of 1 Hz (**G**) and a single discharge of a capacitor resulting in a broader pulse characterized by an initial current peak typically followed by an overshoot with subsequent damped oscillation, i.e., an underdamped transient response (**I**). Each of these primary waves (**A, C, E**) or pulses (**G, I**) were decomposed to their respective constituent harmonic frequency spectrum by Fast Fourier Transformation (FFT) on the right (**B, D, F, H, J**). Continuously oscillating waves (**A, C, E**) are defined by amplitude and the frequency at which, e.g., the electrical current changes its direction, whereas pulses (**G, H**) require the further specification of pulse length and intermitting interval time for a comprehensive description. (**A**) Sine waves are homogenous in a sense that they are composed of only one frequency, i.e., the basal primary frequency, as indicated by a single, discrete peak at 1 Hz in the corresponding FFT spectrum (**B**). The area integral underneath the peak presents a measure for the energy transmitted and the occurrence of quasi only one peak indicates that homogenous sine waves transmit their entire energy by only one frequency. (**C**) Square waves are composed by an infinite, odd harmonic series of frequencies starting with the basal peak at 1 Hz followed by further peaks at 3, 5, 7, 9, etc. Hz with relative amplitudes at about 1, 1/3, 1/5, 1/7, 1/9, etc. (**D**). The sum of all peak integrals equates to the total energy transmitted by the primary wave (**C**). Thus, if there was only one efficient frequency within the FFT spectrum driving therapeutically relevant biological effects, only a fraction of the entire primary wave energy would be available to drive that effect, whereas all the remaining energy contributions relating to the area integrals of the inefficient peaks would be wasted. Obviously, the energy waste would be particularly bad, if the efficient frequency was not the basal one (i.e., in this hypothetical example at 1 Hz) but instead one of the higher orders. In essence, square waves would be therapeutically less efficient as compared to sine waves at same frequency and amplitude. (**E**) Sawtooth waves are composed by an infinite, even harmonic series of frequencies starting with the basal peak at 1 Hz followed by further peaks at 2, 3, 4, 5, 6, 7, 8, 9, etc. Hz with relative amplitudes at about 1/2, 1/4, 1/6, 1/8 etc. (**F**). Thus, if there was only one efficient frequency within the FFT spectrum driving therapeutically relevant biological effects, sawtooth waves would be therapeutically less efficient as compared to sine waves at same frequency and amplitude for the same reasons already stated for square waves (**C, D**). (**G**) Repeated pulses are composed of a more complex spectrum of main and minor frequencies depending on the degree of asymmetric distortion and tailing of the pulses (**H**). If the pulses were of perfect Gaussian shape, their FFT would yield yet another Gaussian function. Conversely for real, i.e. non-Gaussian pulses, if there was only one efficient frequency within the FFT spectrum driving therapeutically relevant biological effects, pulses would be, on the one hand, therapeutically less efficient as compared to sine waves at same frequency and amplitude for the same reasons already

stated for square waves (**C, D**). On the other hand, it is technically much easier and cheaper to generate pulses of much higher amplitudes and resulting peak field strengths as compared to continuously oscillating alternating currents. (**I**) Conversely, a single pulse followed by an overshoot and underdamped transient oscillation resembles a homogenous sine wave with declining amplitude. Thus, the corresponding FFT in panel J reveals, again, a maximum at 1 Hz because the length of one oscillation period (e.g., intial + first overshoot peak) equates to one period of the homogenous sine wave shown in panel A (i.e., 1 s), while the deviation from a perfectly damped response (**I**) causes the asymmetric broadening around the FFT peak at 1 Hz (**J**). Magnetic field pulses, such as those shown in panel I, can be generated using capacitive pulse current sources at very high amplitudes and repeated at intervals of just a few seconds, which makes this method highly efficient.

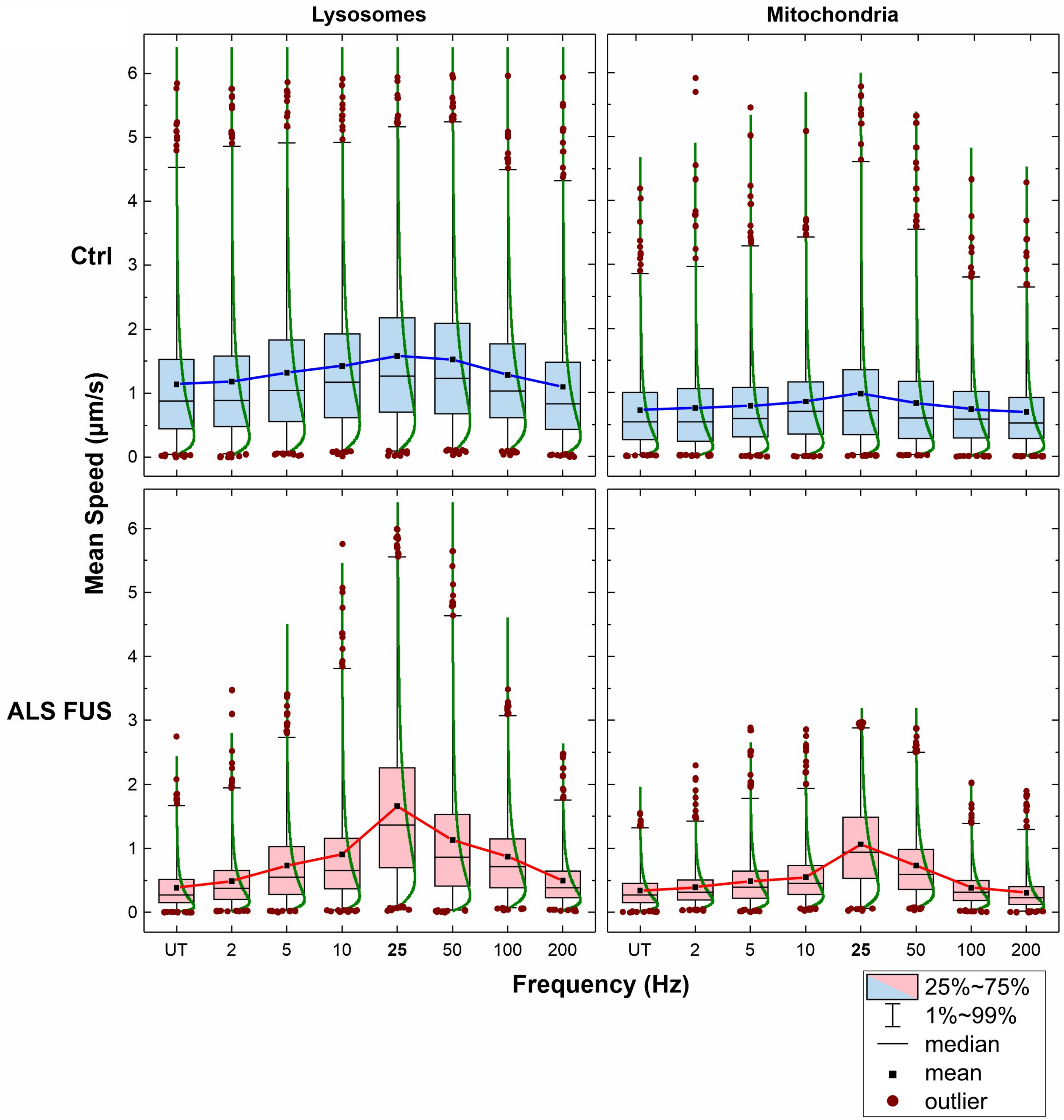

Lysosomes
Mitochondria
Ctrl
ALS FUS
Mean Speed (µm/s)
0
1
2
3
4
5
6
UT
2
5
10
25
50
100
200
Frequency (Hz)
25%~75%
1%~99%
median
mean
outlier

Figure S2: Impact of ACMS on the speed distribution of the entire axonal organelle population at different frequencies. Bulk, quantitative organelle tracking analysis of the isogenic pair of FUS-GFP WT MNs (Ctrl) versus the mutant FUS-GFP P525L (ALS FUS) (Table 1) at the distal microchannel end in MFCs as shown in Figure 1I after the final ACMS session at 48 DIV as shown in Figure 1J. All four consecutive ACMS sessions were performed as shown in Figure 1J at 10 mT and the indicated AC frequency, respectively, and compared against untreated (UT) cells. Individual lysosomes (left) and mitochondria (right) in the entire pool of examined movies were analyzed with respect to their mean speed over the entire movie acquisition time (2 min). Speed distributions over the entire organelle population are presented as box plots as per organelle values (i.e. each data point presents one individual organelle). Box: 25-75% interquartile range, horizontal line: median, square: mean, whiskers: non-outlier range (99% of data), dots outside whiskers: outliers, Ctrl is shown in pale blue, diseased mutant ALS FUS in pale red. Green density regression curves on the right side of each box plot along with the means always located above the medians document the typical top-tailed distributions with the majority of organelles exhibiting low or no motility at all and only a minor fraction of high speed. The line connecting the means reveals the frequency optimum at 25 Hz (Ctrl: in blue; ALS FUS: in red) at which the entire organelle population was up-shifted in its speed distribution. All other organelle tracking results presented in this report are only showing the mean values of the entire organelle populations with their respective standard deviation as error bar for streamlined clarity instead of entire distributions as box plots.

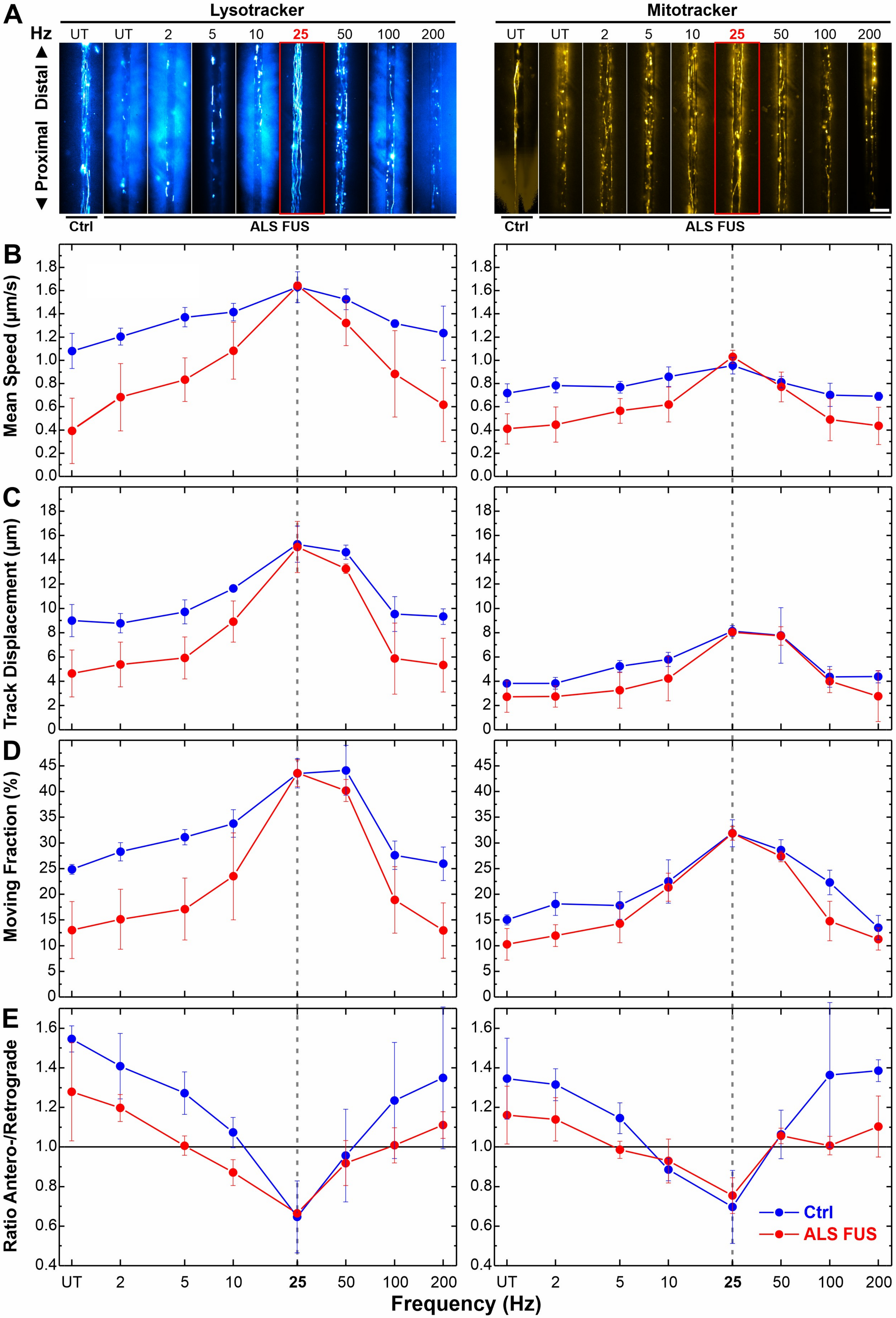

A
Lysotracker
Mitotracker
Hz
UT
2
5
10
25
50
100
200
Distal
Proximal
Ctrl
ALS FUS
B
Mean Speed (µm/s)
C
Track Displacement (µm)
D
Moving Fraction (%)
E
Ratio Antero-/Retrograde
Ctrl
ALS FUS
Frequency (Hz)

Figure S3: Rescue of axonal organelle motility depends on the ACMS frequency with an optimum at 25 Hz. Refers to Figure 2A, B. (**A**) Maximum intensity projections of movies acquired with lysotracker (left, in cyan) and mitotracker (right, in yellow) on the isogenic pair of FUS-GFP WT MNs (Ctrl) versus the mutant FUS-GFP P525L (ALS FUS) (Table 1) at the distal microchannel end in MFCs as shown in Figure 1I after the final ACMS session at 48 DIV as shown in Figure 1J. All ACMS sessions were performed as shown in Figure 1J at 10 mT and the indicated AC frequency, respectively, and compared against untreated (UT) cells. Scale bar = 10 µm; corresponds to Movie S1. Straight organelle movements appear as long trajectories whereas immobile organelles project as punctae. Note the drastically reduced organelle motility in the untreated ALS FUS mutant as compared to untreated Ctrl cells and the rescue through ACMS with a clear optimum at 25 Hz for either type of organelle (boxed in red). (**B-E**) Quantitative tracking analysis of organelle motility as shown in panel A. Individual organelles in the entire pool of examined movies were analyzed with respect to four key tracking parameters (mean speed, track displacement, moving fraction, and ratio antero-/retrograde motility, see below) as a function of ACMS frequency. Data points are the means of the entire analyzed organelle population resulting in samples sizes in the range of typically ten thousands across all pooled movies per condition, i.e. for a specific frequency and cell line, with error bars presenting the standard deviation. Note the clear optimum of ACMS for all tracking parameters (panel B-D) at 25 Hz that corresponded to an inversion of the net movement bias from anterograde to retrograde (panel E). Moreover, note that the stimulation of organelle motility in ALS FUS at 25 Hz was even beyond the natural (UT) Ctrl level and that Ctrl cells responded to that treatment in a similar way, i.e. they were showing the same stimulated motility level. (**B**) Mean speed (y-axis) of either lysosomes (left) or mitochondria (right) plotted over ACMS frequency (x-axis) in the 4 ACMS sessions (Figure 1J). (**C**) Ditto for track displacement. (**D**) Ditto for the moving fraction, i.e., the percentage of the entire organelle population that was defined as mobile fraction showing a track displacement above a threshold of 1.2 µm as opposed to the immobile fraction below that threshold. (**E**) Ditto for the ratio of anterograde over retrograde tracks. Due to the architecture of the MFCs' microgroove (Figure 1I), organelles can move only in one dimension inside the axonal bundles, namely either anterogradely, i.e., towards the distal axonal tips, or retrogradely, i.e., towards the proximal somata. Hence, each track within the mobile organelle fraction can be classified either as anterograde or retrograde. In case an organelle changed its direction during the movie acquisition, the overall net direction was taken for the classification.

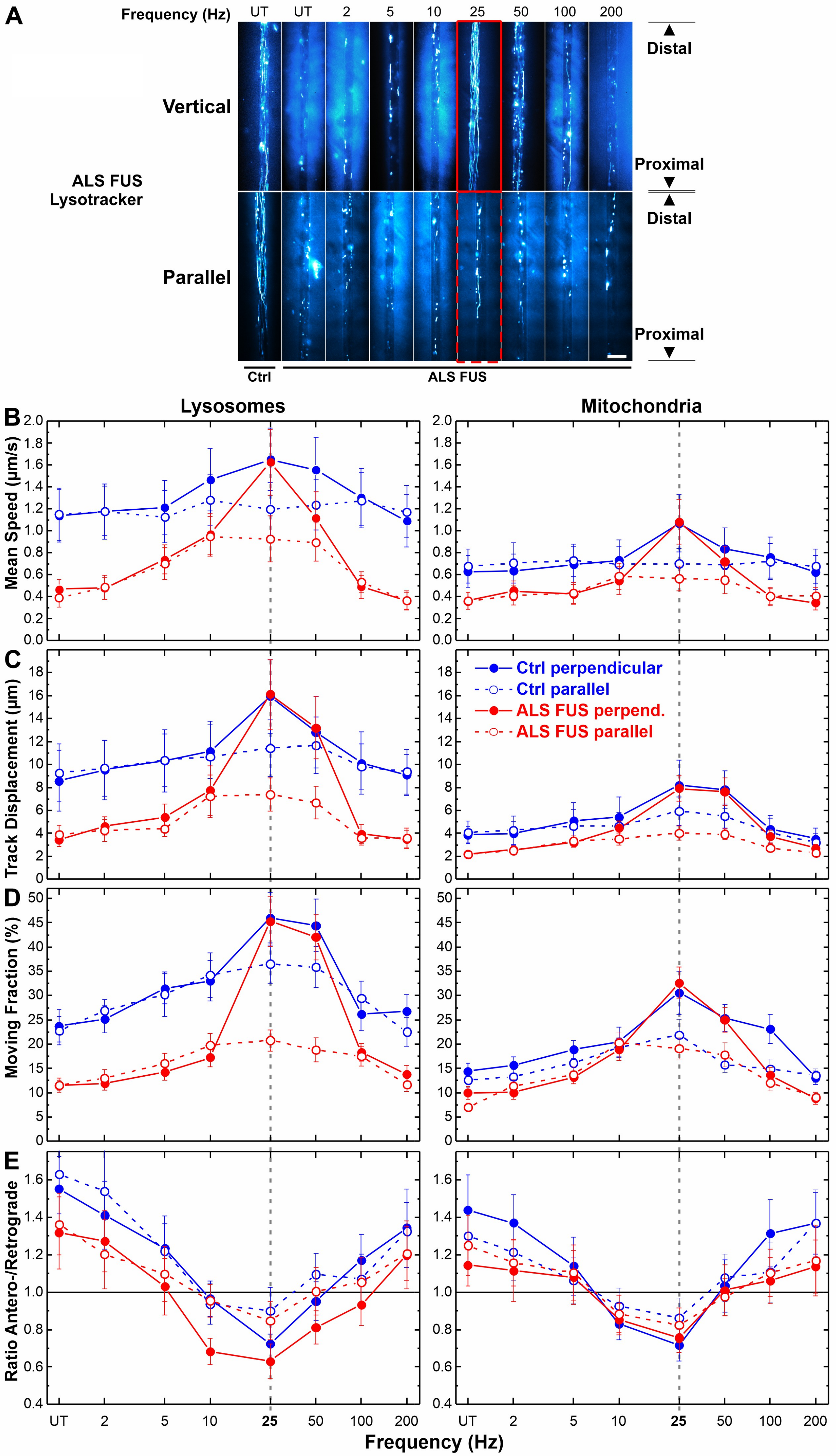

A
Frequency (Hz)
UT
UT
2
5
10
25
50
100
200
Vertical
Parallel
ALS FUS
Lysotracker
Distal
Proximal
Distal
Proximal
Ctrl
ALS FUS
B
C
D
E
Lysosomes
Mitochondria
Mean Speed (µm/s)
Track Displacement (µm)
Moving Fraction (%)
Ratio Antero-/Retrograde
Ctrl perpendicular
Ctrl parallel
ALS FUS perpend.
ALS FUS parallel
Frequency (Hz)

Figure S4: Rescue of axonal organelle motility is more efficient at perpendicular orientation of the magnetic field lines with respect to the axonal plane. Refers to Figure 2C. (**A**) Maximum intensity projections of organelle movies (representative examples acquired with lysotracker) on the isogenic pair of FUS-GFP WT MNs (Ctrl) versus the mutant FUS-GFP P525L (ALS FUS) (Table 1) at the distal microchannel end in MFCs as shown in Figure 1I after the final ACMS session at 48 DIV as shown in Figure 1J. All four consecutive ACMS sessions were performed as shown in Figure 1J at 10 mT and the indicated AC frequency, respectively, and compared against untreated (UT) cells. Top gallery: magnetic field lines were in perpendicular orientation with respect to the axonal plane as in Figure 2-4 and illustrated in Figure 1A-C; bottom gallery: magnetic field lines were in parallel orientation with respect to the axonal plane as illustrated in Figure 1A, D, E. Scale bar = 10 µm; corresponds to Movie S2. Straight organelle movements appear as long trajectories whereas immobile organelles project as punctae. Note the drastically reduced organelle motility in the untreated ALS FUS mutant as compared to untreated Ctrl cells and the rescue through ACMS with a clear optimum at 25 Hz (boxed in red) at perpendicular orientation (Figure 1A-C), whereas this stimulation effect was much less efficient at parallel orientation (Figure 1A, D, E). (**B-E**) Quantitative tracking analysis of organelle motility as shown in panel A. Individual organelles in the entire pool of examined movies were analyzed with respect to four key tracking parameters (mean speed, track displacement, moving fraction, and ratio antero-/retrograde motility, see below) as a function of ACMS frequency, either at perpendicular (Figure 1A-C) (straight curves) or parallel orientation (Figure 1A, D, E) (dashed curves). Data points are the means of the entire analyzed organelle population resulting in samples sizes in the range of typically ten thousands across all pooled movies per condition, i.e. for a specific frequency and cell line, with error bars presenting the standard deviation. Note the clear optimum of ACMS at perpendicular orientation for all tracking parameters (panel B-D) at 25 Hz that corresponded to an inversion of the net movement bias from anterograde to retrograde (panel E), whereas ACMS at parallel orientation was much less efficient. (**B**) Mean speed (y-axis) of either lysosomes (left) or mitochondria (right) plotted over ACMS frequency (x-axis) in the 4 ACMS sessions (Figure 1J) at perpendicular (straight curves) versus parallel (dashed curves) orientation. (**C**) Ditto for track displacement. (**D**) Ditto for the moving fraction, i.e., the percentage of the entire organelle population that was defined as mobile fraction showing a track displacement above a threshold of 1.2 µm as opposed to the immobile fraction below that threshold. (**E**) Ditto for the ratio of anterograde over retrograde tracks. Due to the architecture of the MFCs' microgroove (Figure 1I), organelles can move only in one dimension inside the axonal bundles, namely either anterogradely, i.e., towards the distal axonal tips, or retrogradely, i.e., towards the proximal somata. Hence, each track within the mobile organelle fraction can be classified either as anterograde or retrograde. In case an organelle changed its direction during the movie acquisition, the overall net direction was taken for the classification.

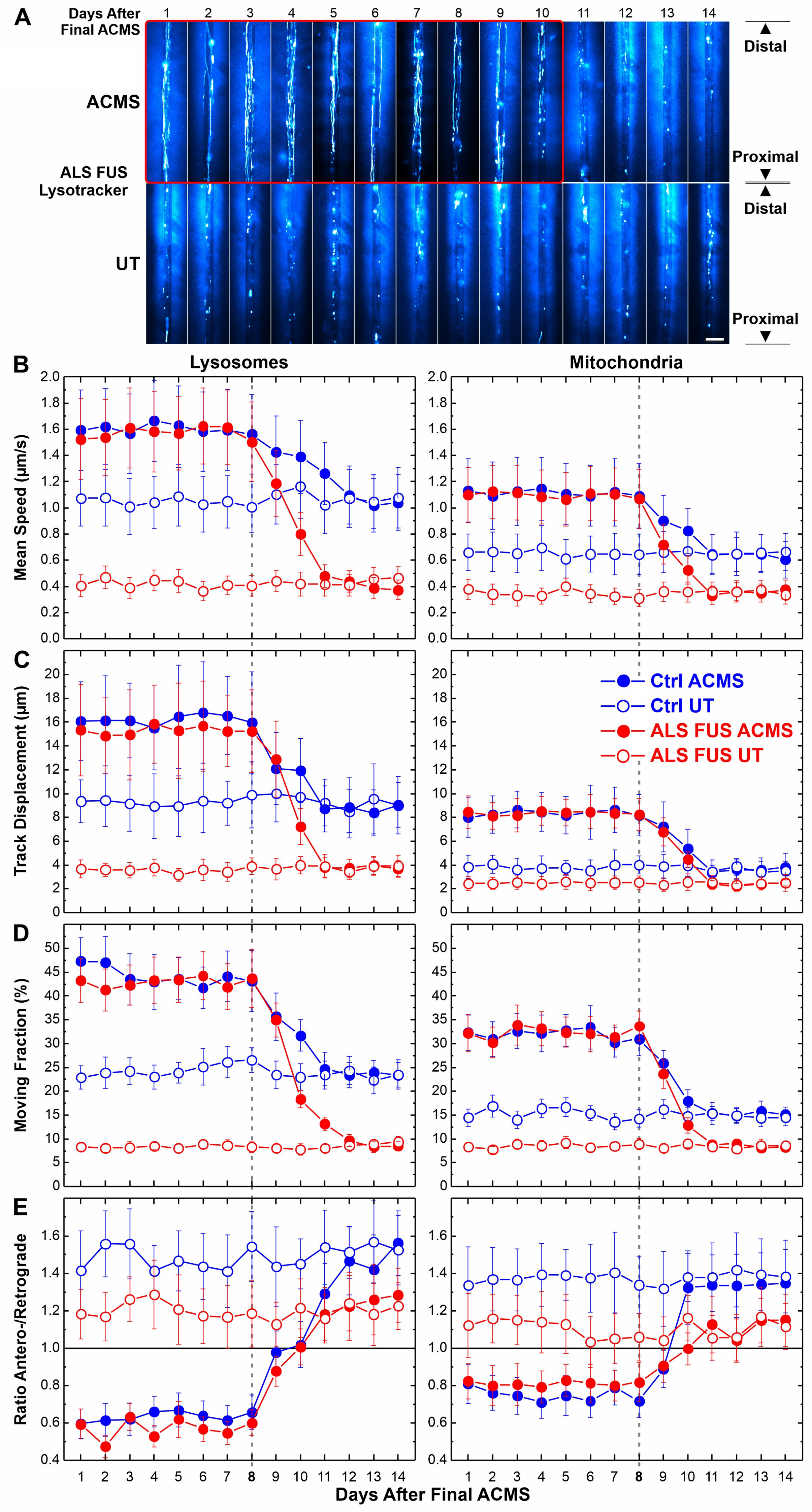

A
Days After Final ACMS
1
2
3
4
5
6
7
8
9
10
11
12
13
14
ACMS
ALS FUS Lysotracker
UT
Distal
Proximal
Distal
Proximal
B
Lysosomes
Mitochondria
Mean Speed (µm/s)
C
Track Displacement (µm)
Ctrl ACMS
Ctrl UT
ALS FUS ACMS
ALS FUS UT
D
Moving Fraction (%)
E
Ratio Antero-/Retrograde
Days After Final ACMS

Figure S5: Rescue of axonal organelle motility is sustained for 8 days beyond the final ACMS session. Refers to Figure 2D. (**A**) Maximum intensity projections of organelle movies (representative examples acquired with lysotracker) of mutant FUS-GFP P525L MNs (ALS FUS) (Table 1) at the distal microchannel end in MFCs as shown in Figure 1I over a chase time course 1-14 days after the final ACMS session at 45 DIV as shown in Figure 1J. All four consecutive ACMS sessions were performed as shown in Figure 1J at 10 mT and the optimal AC frequency of 25 Hz and compared against untreated (UT) cells. Scale bar = 10 μm; corresponds to Movie S3. Straight organelle movements appear as long trajectories whereas immobile organelles project as punctae. Note the drastically reduced organelle motility in the untreated ALS FUS mutant over the entire chase time course (bottom gallery) whereas ACMS-treated cells exhibited a grossly increased organelle motility from the first day after the final ACMS session (Figure 1J) onwards that lasted for approx. 10 days (highlighted in red box) to finally drop to the unstimulated state of the mutant hereafter (top gallery). (**B-E**) Quantitative tracking analysis of organelle motility as shown in panel A of the isogenic pair of FUS-GFP WT MNs (Ctrl) versus the mutant FUS-GFP P525L (ALS FUS) (Table 1). Individual organelles in the entire pool of examined movies were analyzed with respect to four key tracking parameters (mean speed, track displacement, moving fraction, and ratio antero-/retrograde motility, see below) as a function of chase time after the final ACMS session (Figure 1J). Data points are the means of the entire analyzed organelle population resulting in samples sizes in the range of typically ten thousands across all pooled movies per condition, i.e. for a specific chase time and cell line, with error bars presenting the standard deviation. Note the beneficial, stimulatory effect on the motility of both types of organelles (lysosomes and mitochondria) that was fully sustained beyond the final ACMS session (Figure 1J) for at least 8 days in all tracking parameters (panel B-C, compare closed versus open circles). Moreover, note that the stimulation of organelle motility in ALS FUS (panel B-C, red closed versus red open circles) was even beyond the natural (UT) Ctrl level (blue open circles) and that Ctrl cells responded to the ACMS treatment in a similar way, i.e., they were showing the same stimulated motility level (blue closed versus blue open circles). In case of the ratio antero-/retrograde tracks (panel E), the lasting stimulatory effect over 8 days was corresponding to an inversion of the net movement bias from antero- to retrograde. (**B**) Mean speed (y-axis) of either lysosomes (left) or mitochondria (right) plotted over chase time (days) after the final ACMS session (Figure 1J) (x-axis). (**C**) Ditto for track displacement. (**D**) Ditto for the moving fraction, i.e., the percentage of the entire organelle population that was defined as mobile fraction showing a track displacement above a threshold of 1.2 μm as opposed to the immobile fraction below that threshold. (**E**) Ditto for the ratio of anterograde over retrograde tracks. Due to the architecture of the MFCs' microgroove (Figure 1I), organelles can move only in one dimension inside the axonal bundles, namely either anterogradely, i.e., towards the distal axonal tips, or retrogradely, i.e., towards the proximal somata. Hence, each track within the mobile organelle fraction can be classified either as

anterograde or retrograde. In case an organelle changed its direction during the movie acquisition, the overall net direction was taken for the classification.

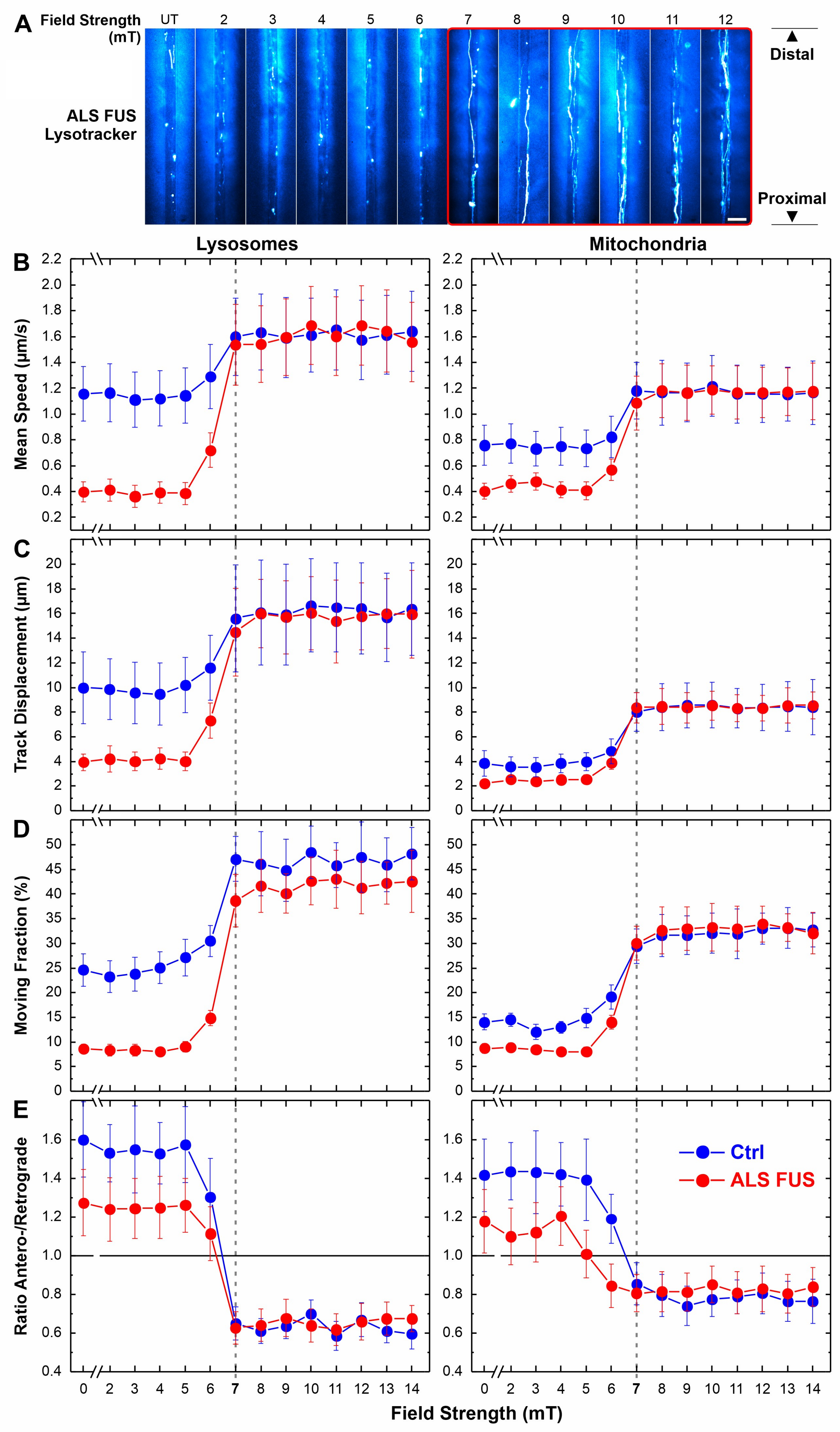
A
Field Strength (mT)
UT 2 3 4 5 6 7 8 9 10 11 12
ALS FUS Lysotracker
Distal
Proximal
Lysosomes
Mitochondria
B
Mean Speed (µm/s)
C
Track Displacement (µm)
D
Moving Fraction (%)
E
Ratio Antero-/Retrograde
Ctrl
ALS FUS
Field Strength (mT)

Figure S6: Rescue of axonal organelle motility requires a field strength of ACMS above a critical threshold of 7 mT. Refers to Figure 2E. (**A**) Maximum intensity projections of organelle movies (representative examples acquired with lysotracker) of mutant FUS-GFP P525L MNs (ALS FUS) (Table 1) at the distal microchannel end in MFCs as shown in Figure 1I after the final ACMS session at 48 DIV as shown in Figure 1J. All four consecutive ACMS sessions were performed as shown in Figure 1J at the optimal AC frequency of 25 Hz and different magnetic field strengths as indicated on top (mT) compared against untreated (UT) cells. Scale bar = 10 µm; corresponds to Movie S4. Straight organelle movements appear as long trajectories whereas immobile organelles project as punctae. Note the drastically reduced organelle motility in the ALS FUS mutant at field strengths below 7 mT that was indistinguishable from untreated cells, whereas ACMS performed at 7 mT and higher resulted in sudden stimulation of organelle motility with long, straight trajectories (highlighted in red box). (**B-E**) Quantitative tracking analysis of organelle motility as shown in panel A of the isogenic pair of FUS-GFP WT MNs (Ctrl) versus the mutant FUS-GFP P525L (ALS FUS) (Table 1). Individual organelles in the entire pool of examined movies were analyzed with respect to four key tracking parameters (mean speed, track displacement, moving fraction, and ratio antero-/retrograde motility, see below) as a function of magnetic field strength applied during the 4 ACMS sessions (Figure 1J). Data points are the means of the entire analyzed organelle population resulting in samples sizes in the range of typically ten thousands across all pooled movies per condition, i.e. for a specific field strength and cell line, with error bars presenting the standard deviation. Note the full stimulatory effect on the motility of both types of organelles (lysosomes and mitochondria) at 7 mT and higher in both lines (Ctrl & ALS FUS) in all tracking parameters (panel B-C), whereas below 7 mT organelle motility remained indistinguishable from untreated cells, i.e., motility was not gradually increasing over field strength but, instead, suddenly switching at the critical threshold of 7 mT from an unstimulated low to a stimulated higher level even beyond untreated Ctrl cells. In case of the ratio antero-/retrograde tracks (panel E), the switch from the low to high motility level was corresponding to an inversion of the net movement bias from antero- to retrograde at 7 mT and higher field strengths. (**B**) Mean speed (y-axis) of either lysosomes (left) or mitochondria (right) plotted over magnetic field strength (mT) in the 4 ACMS sessions (Figure 1J) (x-axis). (**C**) Ditto for track displacement. (**D**) Ditto for the moving fraction, i.e., the percentage of the entire organelle population that was defined as mobile fraction showing a track displacement above a threshold of 1.2 µm as opposed to the immobile fraction below that threshold. (**E**) Ditto for the ratio of anterograde over retrograde tracks. Due to the architecture of the MFCs' microgroove (Figure 1I), organelles can move only in one dimension inside the axonal bundles, namely either anterogradely, i.e., towards the distal axonal tips, or retrogradely, i.e., towards the proximal somata. Hence, each track within the mobile organelle fraction can be classified either as anterograde or retrograde. In case an

organelle changed its direction during the movie acquisition, the overall net direction was taken for the classification.

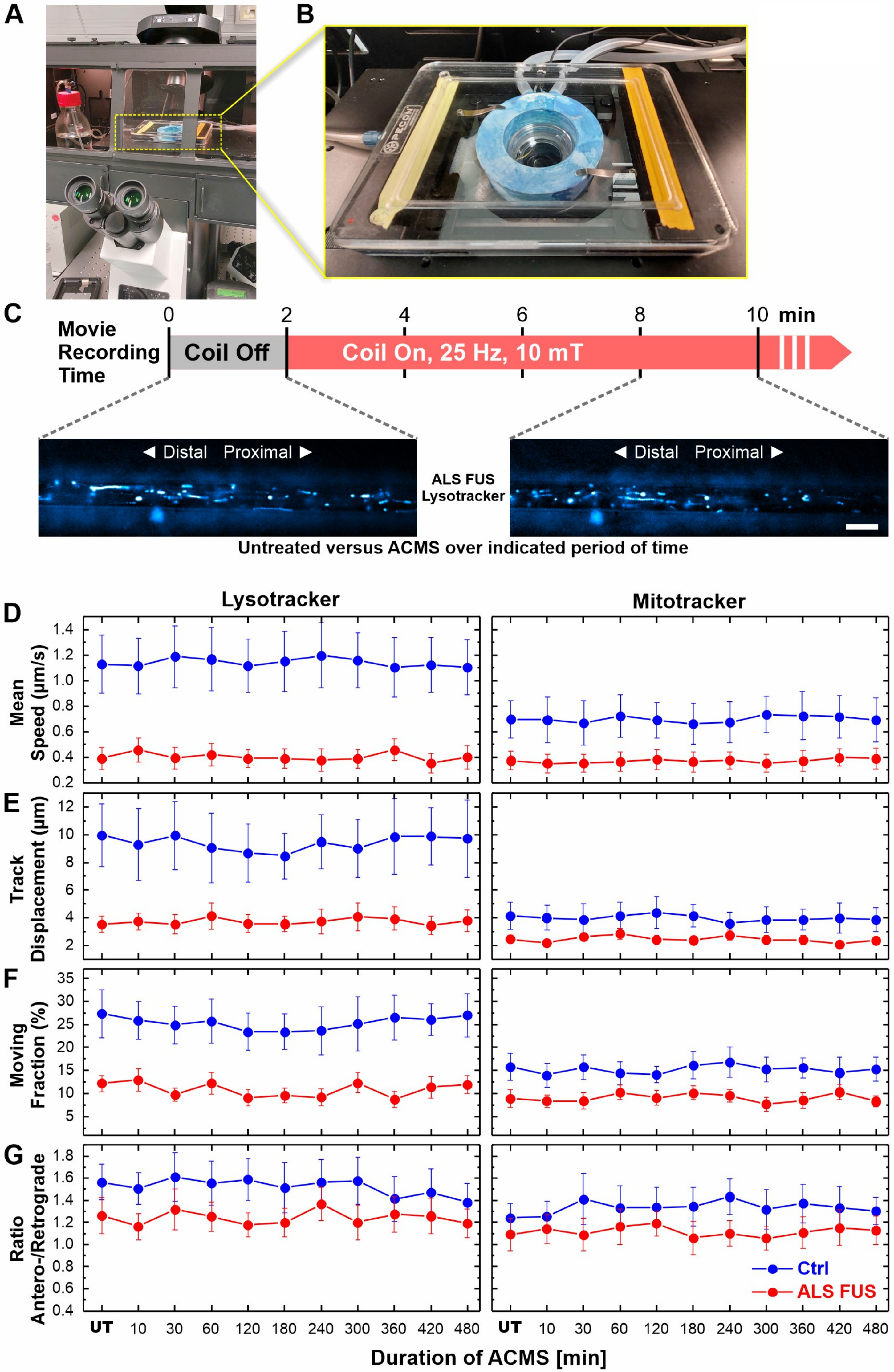

A
B
PECON
C
Movie Recording Time
0
2
4
6
8
10
min
Coil Off
Coil On, 25 Hz, 10 mT
◀ Distal Proximal ▶
ALS FUS Lysotracker
◀ Distal Proximal ▶
Untreated versus ACMS over indicated period of time
Lysotracker
Mitotracker
D
Mean Speed (µm/s)
E
Track Displacement (µm)
F
Moving Fraction (%)
G
Antero-/Retrograde Ratio
UT
10
30
60
120
180
240
300
360
420
480
Duration of ACMS [min]
Ctrl
ALS FUS

Figure S7: ACMS-mediated sustained rescue of axonal organelle motility is due to slow, systemic underlying alterations in the cell rather than ad hoc interactions at moving organelles. (**A**) Experimental set up for ad hoc ACMS at the live imaging microscope during movie acquisition with inserted solenoid on the sample stage. (**B**) Close up of the solenoid encapsulated in blue with connecting tubes for water cooling and black electrical wires. The solenoid was placed directly on the sample holder above the objective inside the CO_2 climate chamber and harbored one 3.5-cm-petri-dish with compartmentalized MNs in an MFC. (**C**) Time line cartoon of ad hoc ACMS during movie recording. MNs of the isogenic pair of FUS-GFP WT (Ctrl) versus the mutant FUS-GFP P525L (ALS FUS) (Table 1) in MFCs at 48 DIV as shown in Figure 1J but not having received any ACMS until this time point (i.e. untreated, UT) were placed on the stage as illustrated in panel B and the movie recording of organelle motility (lyso- and mitotracker) at the distal end of the MFCs' microchannels (Figure 1I) was started (0 min) with the solenoid being switch off (grey phase). After 2 min of movie recording, the solenoid was switched on (red phase) to generate an AC magnetic field of perpendicular orientation with respect to the axonal plane inside the central bore (similar to Figure 1A-C) at 25 Hz and 10 mT. Movie recordings over 2-min-periods, respectively, at time points shown in panel D-G ("Coil On", in red) were compared against the first untreated recording after 2 min ("Coil Off", in grey). Shown underneath the time line as representative example are the maximum intensity projections of the first movie (lysotracker) during ACMS taken after 10 min (right) versus the untreated reference movie (UT) after 2 min. Straight organelle movements appear as long trajectories whereas immobile organelles project as punctae. Note that ACMS applied ad hoc had apparently no effect on organelle motility. Refers to Movie S7. Scale bar = 10 µm. (**D-G**) Quantitative tracking analysis of organelle motility as shown in panel C. Individual organelles in the entire pool of examined movies were analyzed with respect to four key tracking parameters (mean speed, track displacement, moving fraction, and ratio antero-/retrograde motility, see below) at the indicated (x-axis) time points of elapsed ad hoc ACMS (10-480 min) and compared against the first time point when cells were still untreated (2 min, UT). Data points are the means of the entire analyzed organelle population resulting in samples sizes in the range of typically ten thousands across all pooled movies per condition, i.e. for a specific elapsed time of ACMS and cell line, with error bars presenting the standard deviation. Note that ad hoc ACMS did not alter any tracking parameter (**D-G**) even when extended to 480 min. (**D**) Mean speed (y-axis) of either lysosomes (left) or mitochondria (right) plotted over elapsed time of ad hoc ACMS. (**E**) Ditto for track displacement. (**F**) Ditto for the moving fraction, i.e., the percentage of the entire organelle population that was defined as mobile fraction showing a track displacement above a threshold of 1.2 µm as opposed to the immobile fraction below that threshold. (**G**) Ditto for the ratio of anterograde over retrograde tracks. Due to the architecture of the MFCs' microgroove (Figure 1I), organelles can move only in one dimension inside the axonal bundles, namely either anterogradely, i.e., towards the distal axonal tips, or retrogradely, i.e.,

towards the proximal somata. Hence, each track within the mobile organelle fraction can be classified either as anterograde or retrograde. In case an organelle changed its direction during the movie acquisition, the overall net direction was taken for the classification.

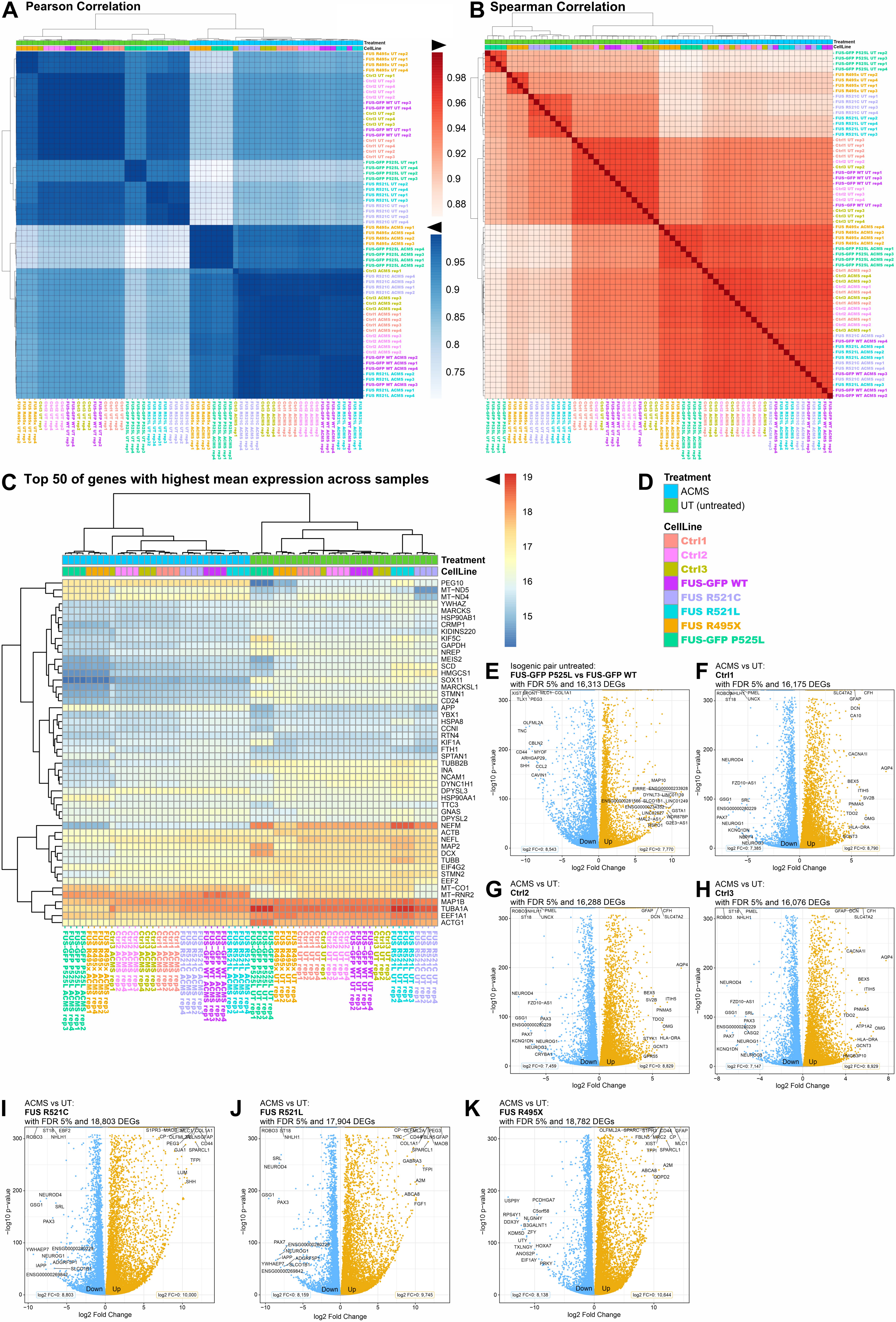

A Pearson Correlation
B Spearman Correlation
Treatment
CellLine
C Top 50 of genes with highest mean expression across samples
Treatment
CellLine
PEG10
MT–ND5
MT–ND4
YWHAZ
MARCKS
HSP90AB1
CRMP1
KIDINS220
KIF5C
GAPDH
NREP
MEIS2
SCD
HMGCS1
SOX11
MARCKSL1
STMN1
CD24
APP
YBX1
HSPA8
CCNI
RTN4
KIF1A
FTH1
SPTAN1
TUBB2B
INA
NCAM1
DYNC1H1
DPYSL3
HSP90AA1
TTC3
GNAS
DPYSL2
NEFM
ACTB
NEFL
MAP2
DCX
TUBB
EIF4G2
STMN2
EEF2
MT–CO1
MT–RNR2
MAP1B
TUBA1A
EEF1A1
ACTG1
D Treatment
ACMS
UT (untreated)
CellLine
Ctrl1
Ctrl2
Ctrl3
FUS-GFP WT
FUS R521C
FUS R521L
FUS R495X
FUS-GFP P525L
E Isogenic pair untreated:
FUS-GFP P525L vs FUS-GFP WT
with FDR 5% and 16,313 DEGs
F ACMS vs UT:
Ctrl1
with FDR 5% and 16,175 DEGs
G ACMS vs UT:
Ctrl2
with FDR 5% and 16,288 DEGs
H ACMS vs UT:
Ctrl3
with FDR 5% and 16,076 DEGs
I ACMS vs UT:
FUS R521C
with FDR 5% and 18,803 DEGs
J ACMS vs UT:
FUS R521L
with FDR 5% and 17,904 DEGs
K ACMS vs UT:
FUS R495X
with FDR 5% and 18,782 DEGs
−log10 p-value
log2 Fold Change
Down
Up

Figure S8: Analysis of all ALS FUS and Ctrl MN samples on normalized expression values of expressed genes. (**A, B**) Heatmaps showing the pair-wise (**A**) Pearson (distance based) (**B**) Spearman's rank (ranking based) correlation between all mutant ALS FUS and WT Ctrl (isogenic and non-isogenic, untreated and ACMS) samples. (**C**) Heatmap of top 50 genes with the highest mean expression strength across all mutant ALS FUS and WT Ctrl (isogenic and non-isogenic, untreated and ACMS) samples. The scale goes from blue to red representing the highest expression. (**D**) Common color code legend for panel A-C. (**E-K**) Volcano plots displaying the distribution of DEGs of the untreated (UT) isogenic pair (FUS-GFP P525L versus FUS-GFP WT) (**E**) and in the three non-isogenic WT Ctrl lines (**F-H**) as well as in the three non-isogenic mutant FUS lines (each ACMS versus UT; Table 1) (**I-K**): $p \leq 0.05$, $FDR \leq 0.05$ and $\log_2$ (Fold Change) ≥ 0.1 or $\log_2$ (Fold Change) ≤ -0.1.

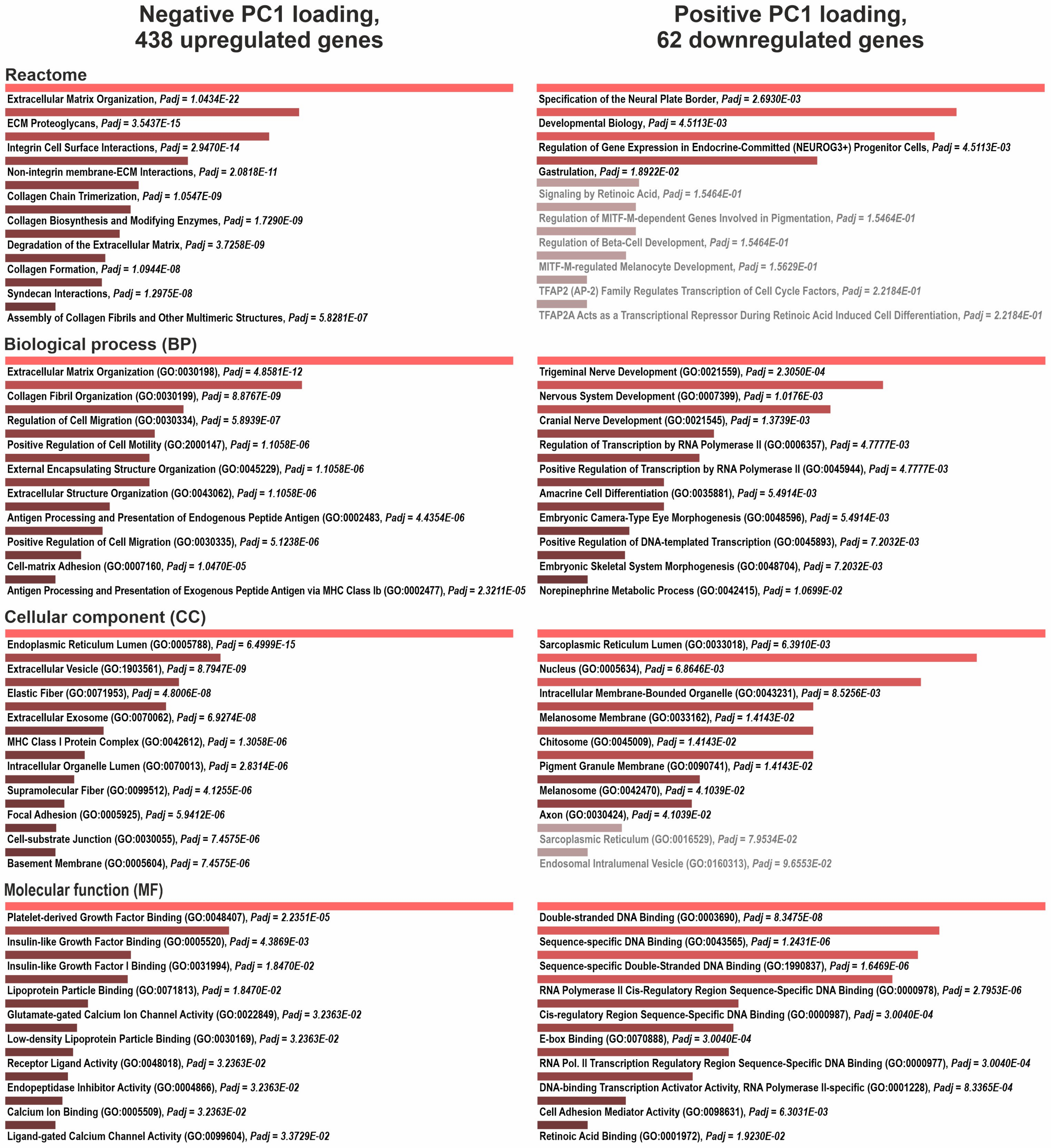
Negative PC1 loading,
438 upregulated genes
Positive PC1 loading,
62 downregulated genes
Reactome
Extracellular Matrix Organization, Padj = 1.0434E-22
ECM Proteoglycans, Padj = 3.5437E-15
Integrin Cell Surface Interactions, Padj = 2.9470E-14
Non-integrin membrane-ECM Interactions, Padj = 2.0818E-11
Collagen Chain Trimerization, Padj = 1.0547E-09
Collagen Biosynthesis and Modifying Enzymes, Padj = 1.7290E-09
Degradation of the Extracellular Matrix, Padj = 3.7258E-09
Collagen Formation, Padj = 1.0944E-08
Syndecan Interactions, Padj = 1.2975E-08
Assembly of Collagen Fibrils and Other Multimeric Structures, Padj = 5.8281E-07
Specification of the Neural Plate Border, Padj = 2.6930E-03
Developmental Biology, Padj = 4.5113E-03
Regulation of Gene Expression in Endocrine-Committed (NEUROG3+) Progenitor Cells, Padj = 4.5113E-03
Gastrulation, Padj = 1.8922E-02
Signaling by Retinoic Acid, Padj = 1.5464E-01
Regulation of MITF-M-dependent Genes Involved in Pigmentation, Padj = 1.5464E-01
Regulation of Beta-Cell Development, Padj = 1.5464E-01
MITF-M-regulated Melanocyte Development, Padj = 1.5629E-01
TFAP2 (AP-2) Family Regulates Transcription of Cell Cycle Factors, Padj = 2.2184E-01
TFAP2A Acts as a Transcriptional Repressor During Retinoic Acid Induced Cell Differentiation, Padj = 2.2184E-01
Biological process (BP)
Extracellular Matrix Organization (GO:0030198), Padj = 4.8581E-12
Collagen Fibril Organization (GO:0030199), Padj = 8.8767E-09
Regulation of Cell Migration (GO:0030334), Padj = 5.8939E-07
Positive Regulation of Cell Motility (GO:2000147), Padj = 1.1058E-06
External Encapsulating Structure Organization (GO:0045229), Padj = 1.1058E-06
Extracellular Structure Organization (GO:0043062), Padj = 1.1058E-06
Antigen Processing and Presentation of Endogenous Peptide Antigen (GO:0002483, Padj = 4.4354E-06
Positive Regulation of Cell Migration (GO:0030335), Padj = 5.1238E-06
Cell-matrix Adhesion (GO:0007160, Padj = 1.0470E-05
Antigen Processing and Presentation of Exogenous Peptide Antigen via MHC Class Ib (GO:0002477), Padj = 2.3211E-05
Trigeminal Nerve Development (GO:0021559), Padj = 2.3050E-04
Nervous System Development (GO:0007399), Padj = 1.0176E-03
Cranial Nerve Development (GO:0021545), Padj = 1.3739E-03
Regulation of Transcription by RNA Polymerase II (GO:0006357), Padj = 4.7777E-03
Positive Regulation of Transcription by RNA Polymerase II (GO:0045944), Padj = 4.7777E-03
Amacrine Cell Differentiation (GO:0035881), Padj = 5.4914E-03
Embryonic Camera-Type Eye Morphogenesis (GO:0048596), Padj = 5.4914E-03
Positive Regulation of DNA-templated Transcription (GO:0045893), Padj = 7.2032E-03
Embryonic Skeletal System Morphogenesis (GO:0048704), Padj = 7.2032E-03
Norepinephrine Metabolic Process (GO:0042415), Padj = 1.0699E-02
Cellular component (CC)
Endoplasmic Reticulum Lumen (GO:0005788), Padj = 6.4999E-15
Extracellular Vesicle (GO:1903561), Padj = 8.7947E-09
Elastic Fiber (GO:0071953), Padj = 4.8006E-08
Extracellular Exosome (GO:0070062), Padj = 6.9274E-08
MHC Class I Protein Complex (GO:0042612), Padj = 1.3058E-06
Intracellular Organelle Lumen (GO:0070013), Padj = 2.8314E-06
Supramolecular Fiber (GO:0099512), Padj = 4.1255E-06
Focal Adhesion (GO:0005925), Padj = 5.9412E-06
Cell-substrate Junction (GO:0030055), Padj = 7.4575E-06
Basement Membrane (GO:0005604), Padj = 7.4575E-06
Sarcoplasmic Reticulum Lumen (GO:0033018), Padj = 6.3910E-03
Nucleus (GO:0005634), Padj = 6.8646E-03
Intracellular Membrane-Bounded Organelle (GO:0043231), Padj = 8.5256E-03
Melanosome Membrane (GO:0033162), Padj = 1.4143E-02
Chitosome (GO:0045009), Padj = 1.4143E-02
Pigment Granule Membrane (GO:0090741), Padj = 1.4143E-02
Melanosome (GO:0042470), Padj = 4.1039E-02
Axon (GO:0030424), Padj = 4.1039E-02
Sarcoplasmic Reticulum (GO:0016529), Padj = 7.9534E-02
Endosomal Intralumenal Vesicle (GO:0160313), Padj = 9.6553E-02
Molecular function (MF)
Platelet-derived Growth Factor Binding (GO:0048407), Padj = 2.2351E-05
Insulin-like Growth Factor Binding (GO:0005520), Padj = 4.3869E-03
Insulin-like Growth Factor I Binding (GO:0031994), Padj = 1.8470E-02
Lipoprotein Particle Binding (GO:0071813), Padj = 1.8470E-02
Glutamate-gated Calcium Ion Channel Activity (GO:0022849), Padj = 3.2363E-02
Low-density Lipoprotein Particle Binding (GO:0030169), Padj = 3.2363E-02
Receptor Ligand Activity (GO:0048018), Padj = 3.2363E-02
Endopeptidase Inhibitor Activity (GO:0004866), Padj = 3.2363E-02
Calcium Ion Binding (GO:0005509), Padj = 3.2363E-02
Ligand-gated Calcium Channel Activity (GO:0099604), Padj = 3.3729E-02
Double-stranded DNA Binding (GO:0003690), Padj = 8.3475E-08
Sequence-specific DNA Binding (GO:0043565), Padj = 1.2431E-06
Sequence-specific Double-Stranded DNA Binding (GO:1990837), Padj = 1.6469E-06
RNA Polymerase II Cis-Regulatory Region Sequence-Specific DNA Binding (GO:0000978), Padj = 2.7953E-06
Cis-regulatory Region Sequence-Specific DNA Binding (GO:0000987), Padj = 3.0040E-04
E-box Binding (GO:0070888), Padj = 3.0040E-04
RNA Pol. II Transcription Regulatory Region Sequence-Specific DNA Binding (GO:0000977), Padj = 3.0040E-04
DNA-binding Transcription Activator Activity, RNA Polymerase II-specific (GO:0001228), Padj = 8.3365E-04
Cell Adhesion Mediator Activity (GO:0098631), Padj = 6.3031E-03
Retinoic Acid Binding (GO:0001972), Padj = 1.9230E-02

Figure S9: Upregulation of ECM pathways are the main drivers towards the novel, unified transcriptomic profile across all wild-type Ctrl and mutant FUS lines (Table 1) upon ACMS. Refers to the PCA plot in Figure 4A. GO and Reactome enrichment analyses of the top 500 variable genes (Table S2?) with the highest absolute loading weigths on principal component 1 (PC1, x-axis in PCA plot in Figure 4A), which is the axis accounting for 83 % of the overall variance of global transcriptomic profiles and primarily driving the sample clustering and the convergence of the ACMS-treated samples. To preserve biological directionality, these 500 genes were separated into their positive and negative loading directions and each analyzed independently for Reactome pathway enrichment and GO analyses in EnrichR for biological processes (BP), cellular component (CC), and molecular function (MF). Negative loadings corresponded to 438 upregulated genes (left bar graphs), positive loadings to just 62 downregulated genes (right bar graphs). PC1 negative loadings present the primary vector driving the spatial convergence of all ACMS-treated samples towards the novel PCA cluster (Figure 4A, encircled in green). Bar graphs are showing the top 10 gene sets for each category (Reactome, BP, CC, MF, Table S3?) ranked by adjusted p-value (Padj) for enrichment relative to the top gene set (100 %), absolute p-values are provided underneath each bar. Gene sets with adjusted p-values above 0.05 (>5E-02) were not considered significant and greyed out. Note the prominent enrichments in ECM-associated upregulated genes among the negative loadings (e.g., Reactome and BP on the left), whereas the few downregulated genes among the positive loadings were assigned to developmental programs and transcriptional regulation (e.g., Reactome and MF on the right). Because these feature loadings represent a uniform mathematical vector across all cell lines and experimental conditions, the spatial convergence of the ACMS-treated cohorts in the PCA plot (Figure 4A) indicates that ACMS overrides baseline genetic background variance by engaging the exact same gene networks in both the Ctrl and mutant FUS cell lines.

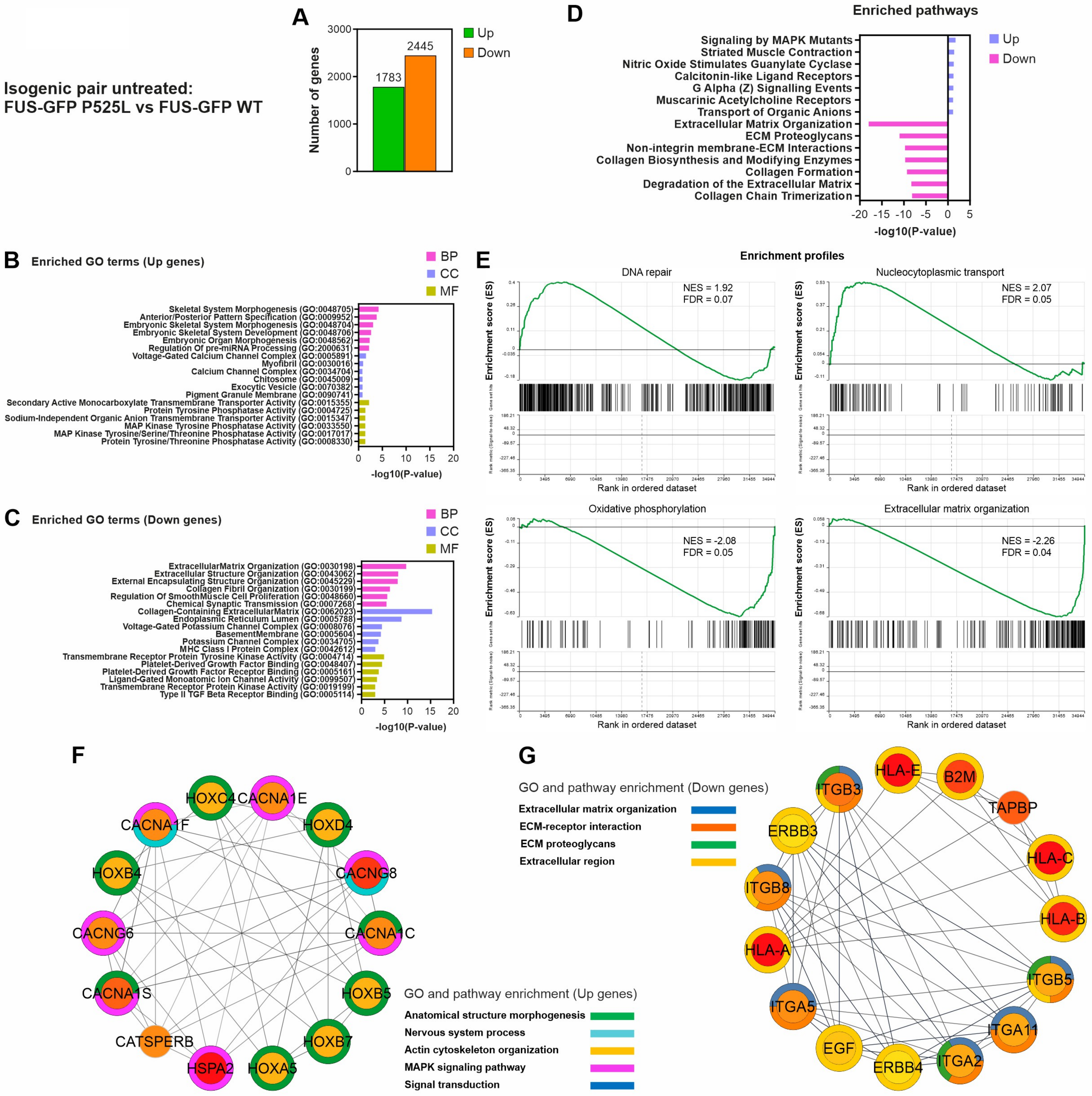
Isogenic pair untreated:
FUS-GFP P525L vs FUS-GFP WT
A
Number of genes
Up
Down
1783
2445
D
Enriched pathways
Signaling by MAPK Mutants
Striated Muscle Contraction
Nitric Oxide Stimulates Guanylate Cyclase
Calcitonin-like Ligand Receptors
G Alpha (Z) Signalling Events
Muscarinic Acetylcholine Receptors
Transport of Organic Anions
Extracellular Matrix Organization
ECM Proteoglycans
Non-integrin membrane-ECM Interactions
Collagen Biosynthesis and Modifying Enzymes
Collagen Formation
Degradation of the Extracellular Matrix
Collagen Chain Trimerization
-log10(P-value)
B
Enriched GO terms (Up genes)
BP
CC
MF
Skeletal System Morphogenesis (GO:0048705)
Anterior/Posterior Pattern Specification (GO:0009952)
Embryonic Skeletal System Morphogenesis (GO:0048704)
Embryonic Skeletal System Development (GO:0048706)
Embryonic Organ Morphogenesis (GO:0048562)
Regulation Of pre-miRNA Processing (GO:2000631)
Voltage-Gated Calcium Channel Complex (GO:0005891)
Myofibril (GO:0030016)
Calcium Channel Complex (GO:0034704)
Chitosome (GO:0045009)
Exocytic Vesicle (GO:0070382)
Pigment Granule Membrane (GO:0090741)
Secondary Active Monocarboxylate Transmembrane Transporter Activity (GO:0015355)
Protein Tyrosine Phosphatase Activity (GO:0004725)
Sodium-Independent Organic Anion Transmembrane Transporter Activity (GO:0015347)
MAP Kinase Tyrosine Phosphatase Activity (GO:0033550)
MAP Kinase Tyrosine/Serine/Threonine Phosphatase Activity (GO:0017017)
Protein Tyrosine/Threonine Phosphatase Activity (GO:0008330)
-log10(P-value)
C
Enriched GO terms (Down genes)
BP
CC
MF
ExtracellularMatrix Organization (GO:0030198)
Extracellular Structure Organization (GO:0043062)
External Encapsulating Structure Organization (GO:0045229)
Collagen Fibril Organization (GO:0030199)
Regulation Of SmoothMuscle Cell Proliferation (GO:0048660)
Chemical Synaptic Transmission (GO:0007268)
Collagen-Containing ExtracellularMatrix (GO:0062023)
Endoplasmic Reticulum Lumen (GO:0005788)
Voltage-Gated Potassium Channel Complex (GO:0008076)
BasementMembrane (GO:0005604)
Potassium Channel Complex (GO:0034705)
MHC Class I Protein Complex (GO:0042612)
Transmembrane Receptor Protein Tyrosine Kinase Activity (GO:0004714)
Platelet-Derived Growth Factor Binding (GO:0048407)
Platelet-Derived Growth Factor Receptor Binding (GO:0005161)
Ligand-Gated Monoatomic Ion Channel Activity (GO:0099507)
Transmembrane Receptor Protein Kinase Activity (GO:0019199)
Type II TGF Beta Receptor Binding (GO:0005114)
-log10(P-value)
E
Enrichment profiles
DNA repair
NES = 1.92
FDR = 0.07
Nucleocytoplasmic transport
NES = 2.07
FDR = 0.05
Oxidative phosphorylation
NES = -2.08
FDR = 0.05
Extracellular matrix organization
NES = -2.26
FDR = 0.04
Enrichment score (ES)
Rank in ordered dataset
F
HOXC4
CACNA1E
CACNA1F
HOXD4
HOXB4
CACNG8
CACNG6
CACNA1C
CACNA1S
HOXB5
CATSPERB
HOXB7
HSPA2
HOXA5
GO and pathway enrichment (Up genes)
Anatomical structure morphogenesis
Nervous system process
Actin cytoskeleton organization
MAPK signaling pathway
Signal transduction
G
GO and pathway enrichment (Down genes)
Extracellular matrix organization
ECM-receptor interaction
ECM proteoglycans
Extracellular region
HLA-E
B2M
ITGB3
TAPBP
ERBB3
HLA-C
ITGB8
HLA-B
HLA-A
ITGB5
ITGA5
ITGA11
EGF
ERBB4
ITGA2

Figure S10: Functional enrichment analysis and PPI analysis of DEGs in the untreated isogenic pair of FUS-GFP P525L versus FUS-GFP WT MNs. (**A**) Statistics of the results of differentially expressed genes (DEGs). (**B-D**) Functional analysis of enriched genes identified a variety of (**B**) upregulated and (**C**) downregulated GO terms and (**D**) enriched pathways (Reactome and KEGG) associated with untreated FUS-GFP P525L versus FUS-GFP WT MNs ($p \leq 0.05$). Gene ontology (GO) term enrichment analysis of differential expression genes ranked the most enriched gene group changes in untreated FUS-GFP P525L MNs (versus untreated FUS-GFP WT). The enrichment analysis was performed by EnrichR and significant terms/pathways were listed with a p-value ≤ 0.05. The GO terms were divided into three categories, including biological process (BP, pink), molecular function (MF, olive), and cellular component (CC, purple), respectively. (**E**) GSEAs were performed using normalized counts from the DESeq2 output ranked list in untreated FUS-GFP P525L mutant datasets (versus untreated FUS-GFP WT), $p \leq 0.05$, FDR ≤ 0.05 was considered statistically significant. The top two most significant enriched gene sets are shown that positively (top row) and negatively (bottom row) correlated with the gene counts based on NES values. The y-axis represents enrichment scores (green curve) across the entire gene set for each individual gene member on the x-axis (vertical black bars) of that gene set. Top 15 (**F**) upregulated and (**G**) downregulated hub genes as deduced from the corresponding PPI networks (by STRING analysis) shown in Figure S20. Hub genes were identified by maximal clique centrality (MCC) algorithm using Cytoscape (cytoHubba plugin), the deeper the color, the higher the gene rank.

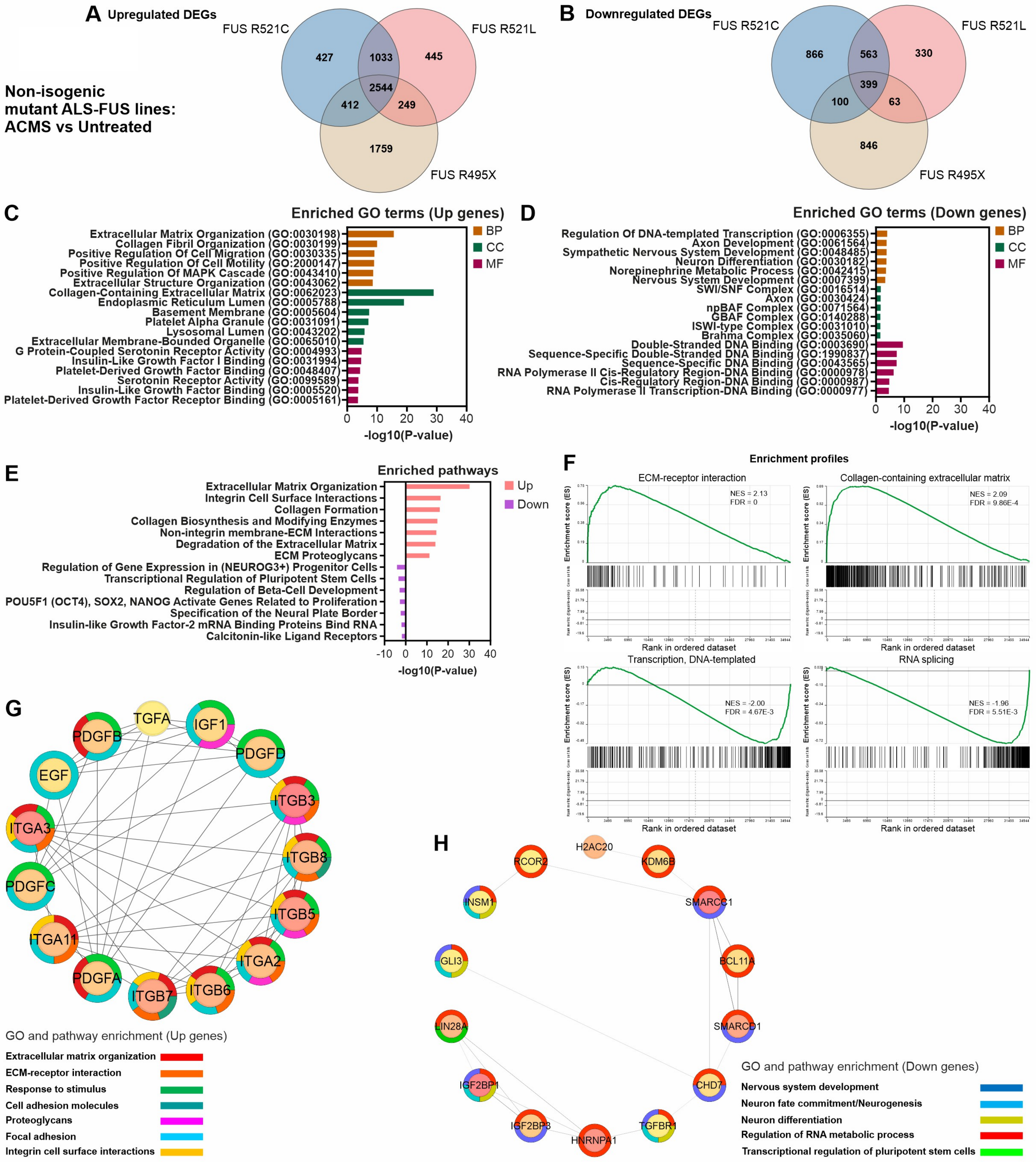
A Upregulated DEGs
FUS R521C
FUS R521L
FUS R495X
427
1033
445
2544
412
249
1759
Non-isogenic
mutant ALS-FUS lines:
ACMS vs Untreated
B Downregulated DEGs
FUS R521C
FUS R521L
FUS R495X
866
563
330
399
100
63
846
C
Enriched GO terms (Up genes)
Extracellular Matrix Organization (GO:0030198)
Collagen Fibril Organization (GO:0030199)
Positive Regulation Of Cell Migration (GO:0030335)
Positive Regulation Of Cell Motility (GO:2000147)
Positive Regulation Of MAPK Cascade (GO:0043410)
Extracellular Structure Organization (GO:0043062)
Collagen-Containing Extracellular Matrix (GO:0062023)
Endoplasmic Reticulum Lumen (GO:0005788)
Basement Membrane (GO:0005604)
Platelet Alpha Granule (GO:0031091)
Lysosomal Lumen (GO:0043202)
Extracellular Membrane-Bounded Organelle (GO:0065010)
G Protein-Coupled Serotonin Receptor Activity (GO:0004993)
Insulin-Like Growth Factor I Binding (GO:0031994)
Platelet-Derived Growth Factor Binding (GO:0048407)
Serotonin Receptor Activity (GO:0099589)
Insulin-Like Growth Factor Binding (GO:0005520)
Platelet-Derived Growth Factor Receptor Binding (GO:0005161)
BP
CC
MF
-log10(P-value)
D
Enriched GO terms (Down genes)
Regulation Of DNA-templated Transcription (GO:0006355)
Axon Development (GO:0061564)
Sympathetic Nervous System Development (GO:0048485)
Neuron Differentiation (GO:0030182)
Norepinephrine Metabolic Process (GO:0042415)
Nervous System Development (GO:0007399)
SWI/SNF Complex (GO:0016514)
Axon (GO:0030424)
npBAF Complex (GO:0071564)
GBAF Complex (GO:0140288)
ISWI-type Complex (GO:0031010)
Brahma Complex (GO:0035060)
Double-Stranded DNA Binding (GO:0003690)
Sequence-Specific Double-Stranded DNA Binding (GO:1990837)
Sequence-Specific DNA Binding (GO:0043565)
RNA Polymerase II Cis-Regulatory Region-DNA Binding (GO:0000978)
Cis-Regulatory Region-DNA Binding (GO:0000987)
RNA Polymerase II Transcription-DNA Binding (GO:0000977)
BP
CC
MF
-log10(P-value)
E
Enriched pathways
Extracellular Matrix Organization
Integrin Cell Surface Interactions
Collagen Formation
Collagen Biosynthesis and Modifying Enzymes
Non-integrin membrane-ECM Interactions
Degradation of the Extracellular Matrix
ECM Proteoglycans
Regulation of Gene Expression in (NEUROG3+) Progenitor Cells
Transcriptional Regulation of Pluripotent Stem Cells
Regulation of Beta-Cell Development
POU5F1 (OCT4), SOX2, NANOG Activate Genes Related to Proliferation
Specification of the Neural Plate Border
Insulin-like Growth Factor-2 mRNA Binding Proteins Bind RNA
Calcitonin-like Ligand Receptors
Up
Down
-log10(P-value)
F
Enrichment profiles
ECM-receptor interaction
NES = 2.13
FDR = 0
Collagen-containing extracellular matrix
NES = 2.09
FDR = 9.86E-4
Transcription, DNA-templated
NES = -2.00
FDR = 4.67E-3
RNA splicing
NES = -1.96
FDR = 5.51E-3
Enrichment score (ES)
Rank in ordered dataset
G
TGFA
IGF1
PDGFB
PDGFD
EGF
ITGB3
ITGA3
ITGB8
PDGFC
ITGB5
ITGA11
ITGA2
PDGFA
ITGB7
ITGB6
GO and pathway enrichment (Up genes)
Extracellular matrix organization
ECM-receptor interaction
Response to stimulus
Cell adhesion molecules
Proteoglycans
Focal adhesion
Integrin cell surface interactions
H
H2AC20
RCOR2
KDM6B
INSM1
SMARCC1
GLI3
BCL11A
LIN28A
SMARCD1
IGF2BP1
CHD7
IGF2BP3
TGFBR1
HNRNPA1
GO and pathway enrichment (Down genes)
Nervous system development
Neuron fate commitment/Neurogenesis
Neuron differentiation
Regulation of RNA metabolic process
Transcriptional regulation of pluripotent stem cells

Figure S11: Functional enrichment analysis and PPI analysis of DEGs in the three lines of non-isogenic mutant FUS MNs. (**A, B**) Venn diagram identifying the overlap of (**A**) 2544 commonly upregulated (**B**) 399 commonly downregulated genes between DEGs in mutant FUS datasets (ACMS versus untreated for each non-isogenic line, respectively; Table 1). (**C-E**) Functional analysis of common genes limited to the Venn diagram overlaps in panel A and B identified a variety of (**C**) upregulated and (**D**) downregulated GO terms and (**E**) enriched pathways (Reactome and KEGG) associated with mutant FUS MNs ($p \leq 0.05$). Gene ontology (GO) term enrichment analysis of DEGs ranked the most enriched common gene group changes in all three mutant FUS lines through ACMS. The enrichment analysis was performed by EnrichR and significant terms/pathways were listed with a p-value ≤ 0.05. The GO terms were divided into three categories, including biological process (BP, dark orange), molecular function (MF, dark green), and cellular component (CC, brown), respectively. (**F**) GSEAs were performed using normalized counts from the DESeq2 output ranked list in pooled non-isogenic mutant FUS datasets (each ACMS versus untreated), $p \leq 0.05$, FDR ≤ 0.05 was considered statistically significant. The top two most significant enriched gene sets are shown that positively (top row) and negatively (bottom row) correlated with the gene counts based on NES values. The y-axis represents enrichment scores (green curve) across the entire gene set for each individual gene member on the x-axis (vertical black bars) of that gene set. Top 15 (**G**) upregulated and (**H**) downregulated hub genes as deduced from the corresponding PPI networks (by STRING analysis) shown in Figure S22. Hub genes were identified by maximal clique centrality (MCC) algorithm using Cytoscape (cytoHubba plugin), the deeper the color, the higher the gene rank.

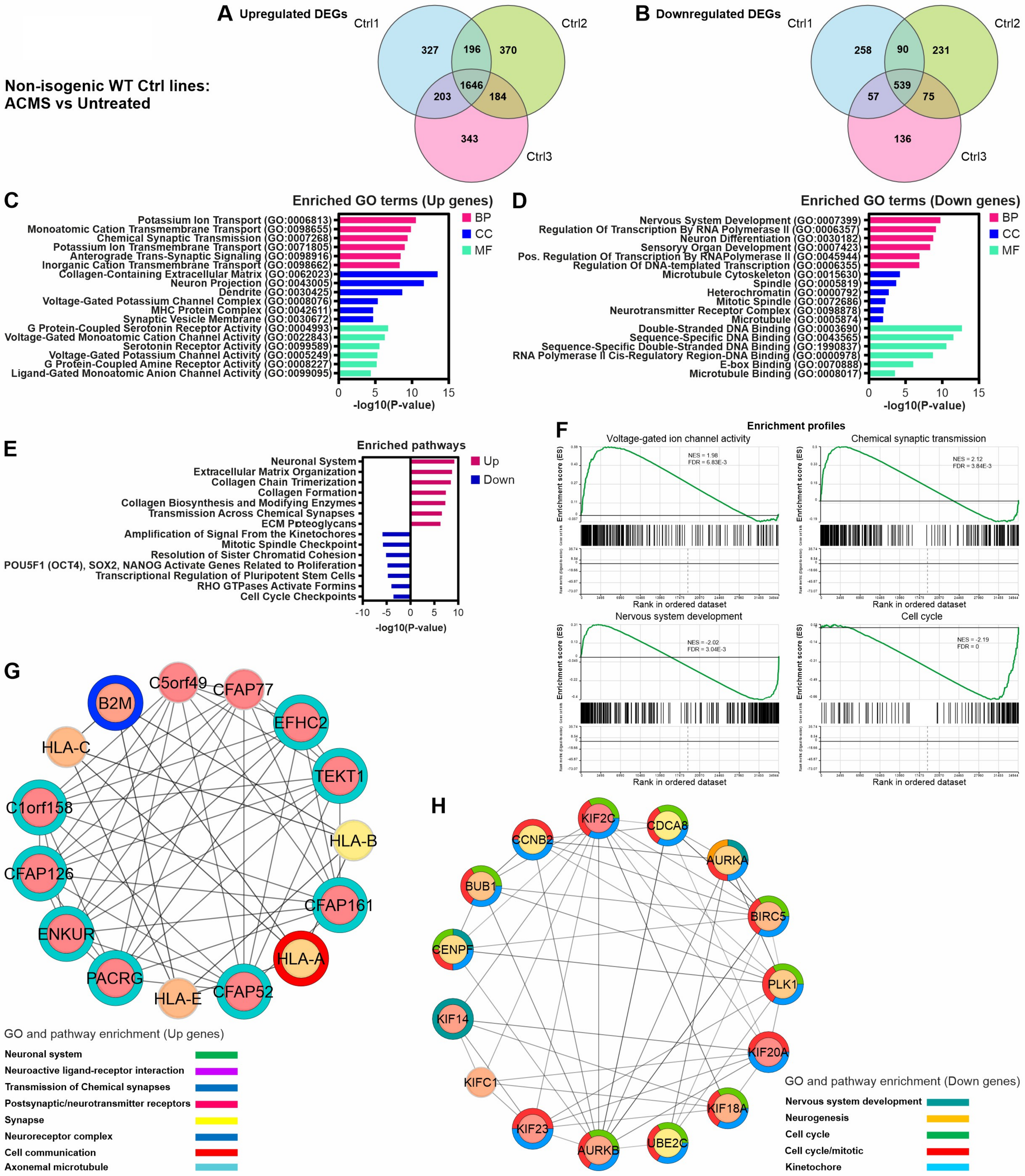

A Upregulated DEGs
Ctrl1
Ctrl2
Ctrl3
327
196
370
203
1646
184
343
Non-isogenic WT Ctrl lines:
ACMS vs Untreated
B Downregulated DEGs
258
90
231
57
539
75
136
C Enriched GO terms (Up genes)
Potassium Ion Transport (GO:0006813)
Monoatomic Cation Transmembrane Transport (GO:0098655)
Chemical Synaptic Transmission (GO:0007268)
Potassium Ion Transmembrane Transport (GO:0071805)
Anterograde Trans-Synaptic Signaling (GO:0098916)
Inorganic Cation Transmembrane Transport (GO:0098662)
Collagen-Containing Extracellular Matrix (GO:0062023)
Neuron Projection (GO:0043005)
Dendrite (GO:0030425)
Voltage-Gated Potassium Channel Complex (GO:0008076)
MHC Protein Complex (GO:0042611)
Synaptic Vesicle Membrane (GO:0030672)
G Protein-Coupled Serotonin Receptor Activity (GO:0004993)
Voltage-Gated Monoatomic Cation Channel Activity (GO:0022843)
Serotonin Receptor Activity (GO:0099589)
Voltage-Gated Potassium Channel Activity (GO:0005249)
G Protein-Coupled Amine Receptor Activity (GO:0008227)
Ligand-Gated Monoatomic Anion Channel Activity (GO:0099095)
BP
CC
MF
-log10(P-value)
D Enriched GO terms (Down genes)
Nervous System Development (GO:0007399)
Regulation Of Transcription By RNA Polymerase II (GO:0006357)
Neuron Differentiation (GO:0030182)
Sensoryy Organ Development (GO:0007423)
Pos. Regulation Of Transcription By RNAPolymerase II (GO:0045944)
Regulation Of DNA-templated Transcription (GO:0006355)
Microtubule Cytoskeleton (GO:0015630)
Spindle (GO:0005819)
Heterochromatin (GO:0000792)
Mitotic Spindle (GO:0072686)
Neurotransmitter Receptor Complex (GO:0098878)
Microtubule (GO:0005874)
Double-Stranded DNA Binding (GO:0003690)
Sequence-Specific DNA Binding (GO:0043565)
Sequence-Specific Double-Stranded DNA Binding (GO:1990837)
RNA Polymerase II Cis-Regulatory Region-DNA Binding (GO:0000978)
E-box Binding (GO:0070888)
Microtubule Binding (GO:0008017)
E Enriched pathways
Neuronal System
Extracellular Matrix Organization
Collagen Chain Trimerization
Collagen Formation
Collagen Biosynthesis and Modifying Enzymes
Transmission Across Chemical Synapses
ECM Pteoglycans
Amplification of Signal From the Kinetochores
Mitotic Spindle Checkpoint
Resolution of Sister Chromatid Cohesion
POU5F1 (OCT4), SOX2, NANOG Activate Genes Related to Proliferation
Transcriptional Regulation of Pluripotent Stem Cells
RHO GTPases Activate Formins
Cell Cycle Checkpoints
Up
Down
-log10(P-value)
F Enrichment profiles
Voltage-gated ion channel activity
NES = 1.98
FDR = 6.83E-3
Chemical synaptic transmission
NES = 2.12
FDR = 3.84E-3
Nervous system development
NES = -2.02
FDR = 3.04E-3
Cell cycle
NES = -2.19
FDR = 0
Enrichment score (ES)
Rank in ordered dataset
G
B2M
C5orf49
CFAP77
EFHC2
HLA-C
TEKT1
C1orf158
HLA-B
CFAP126
CFAP161
ENKUR
HLA-A
PACRG
HLA-E
CFAP52
GO and pathway enrichment (Up genes)
Neuronal system
Neuroactive ligand-receptor interaction
Transmission of Chemical synapses
Postsynaptic/neurotransmitter receptors
Synapse
Neuroreceptor complex
Cell communication
Axonemal microtubule
H
KIF2C
CDCA8
CCNB2
AURKA
BUB1
BIRC5
CENPF
PLK1
KIF14
KIF20A
KIFC1
KIF18A
KIF23
AURKB
UBE2C
GO and pathway enrichment (Down genes)
Nervous system development
Neurogenesis
Cell cycle
Cell cycle/mitotic
Kinetochore

Figure S12: Functional enrichment analysis and PPI analysis of DEGs in the three lines of non-isogenic WT Ctrl MNs. (**A, B**) Venn diagram identifying the overlap of (**A**) 1646 commonly upregulated (**B**) 539 commonly downregulated genes between DEGs in WT Ctrl datasets (ACMS versus untreated for each non-isogenic line, respectively; Table 1). (**C-E**) Functional analysis of common genes limited to the Venn diagram overlaps in panel A and B identified a variety of (**C**) upregulated and (**D**) downregulated GO terms and (**E**) enriched pathways (Reactome and KEGG) associated with WT Ctrl MNs ($p \leq 0.05$). Gene ontology (GO) term enrichment analysis of DEGs ranked the most enriched common gene group changes in all three WT Ctrl lines through ACMS. The enrichment analysis was performed by EnrichR and significant terms/pathways were listed with a p-value ≤ 0.05. The GO terms were divided into three categories, including biological process (BP, red), molecular function (MF, pale green), and cellular component (CC, blue), respectively. (**F**) GSEAs were performed using normalized counts from the DESeq2 output ranked list in pooled non-isogenic WT Ctrl datasets (each ACMS versus untreated), $p \leq 0.05$, FDR ≤ 0.05 was considered statistically significant. The top two most significant enriched gene sets are shown that positively (top row) and negatively (bottom row) correlated with the gene counts based on NES values. The y-axis represents enrichment scores (green curve) across the entire gene set for each individual gene member on the x-axis (vertical black bars) of that gene set. Top 15 (**G**) upregulated and (**H**) downregulated hub genes as deduced from the corresponding PPI networks (by STRING analysis) shown in Figure S23. Hub genes were identified by maximal clique centrality (MCC) algorithm using Cytoscape (cytoHubba plugin), the deeper the color, the higher the gene rank.

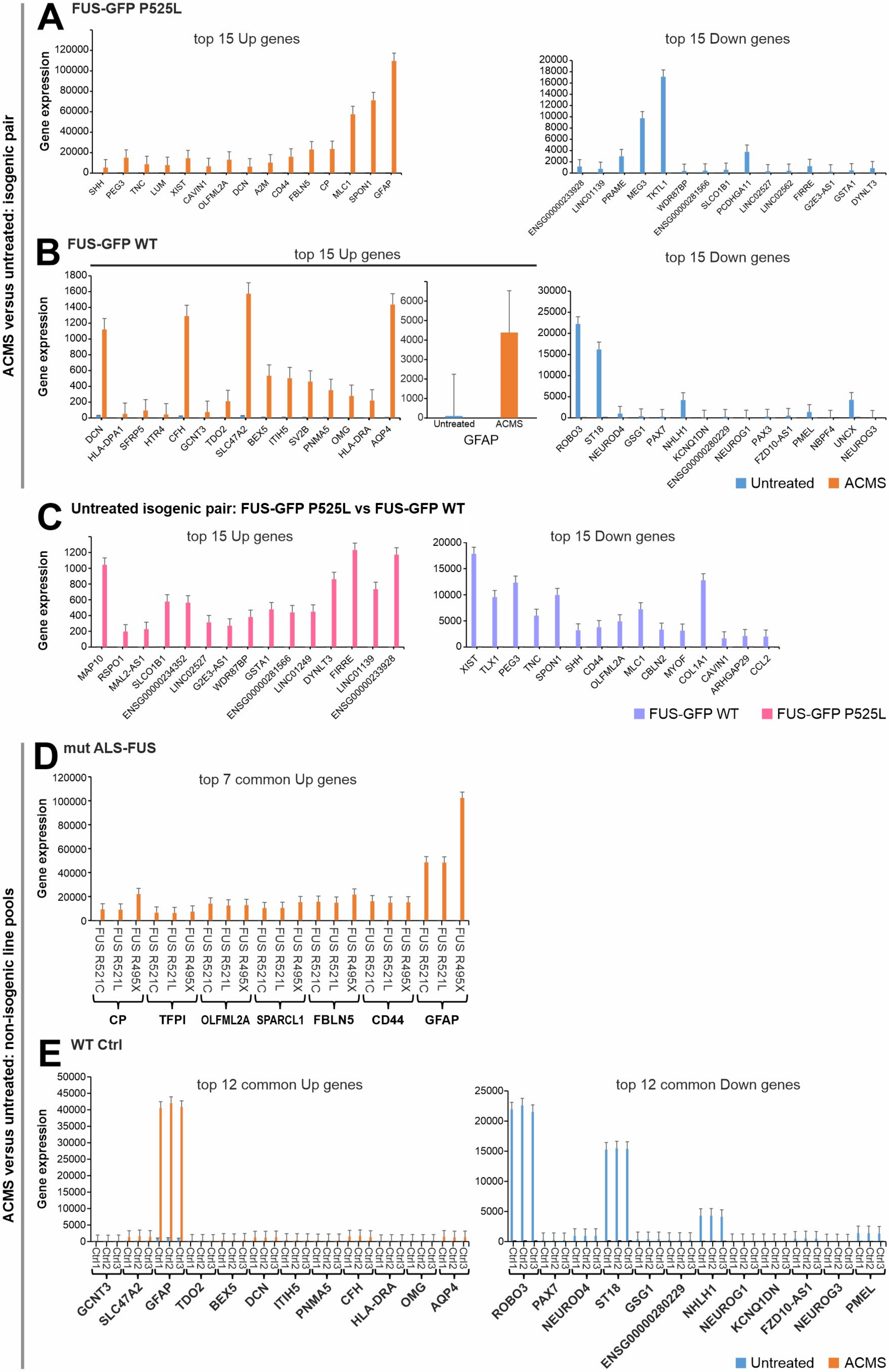

ACMS versus untreated: isogenic pair
A
FUS-GFP P525L
top 15 Up genes
Gene expression
SHH PEG3 TNC LUM XIST CAVIN1 OLFML2A DCN A2M CD44 FBLN5 CP MLC1 SPON1 GFAP
top 15 Down genes
ENSG00000233928 LINC01139 PRAME MEG3 TKTL1 WDR87BP ENSG00000281566 SLCO1B1 PCDHGA11 LINC02527 LINC02562 FIRRE G2E3-AS1 GSTA1 DYNLT3
B
FUS-GFP WT
top 15 Up genes
Gene expression
DCN HLA-DPA1 SFRP5 HTR4 CFH GCNT3 TDO2 SLC47A2 BEX5 ITIH5 SV2B PNMA5 OMG HLA-DRA AQP4
Untreated ACMS
GFAP
top 15 Down genes
ROBO3 ST18 NEUROD4 GSG1 PAX7 NHLH1 KCNQ1DN ENSG00000280229 NEUROG1 PAX3 FZD10-AS1 PMEL NBPF4 UNCX NEUROG3
Untreated ACMS
C
Untreated isogenic pair: FUS-GFP P525L vs FUS-GFP WT
top 15 Up genes
Gene expression
MAP10 RSPO1 MAL2-AS1 SLCO1B1 ENSG00000234352 LINC02527 G2E3-AS1 WDR87BP GSTA1 ENSG00000281566 LINC01249 DYNLT3 FIRRE LINC01139 ENSG00000233928
top 15 Down genes
XIST TLX1 PEG3 TNC SPON1 SHH CD44 OLFML2A MLC1 CBLN2 MYOF COL1A1 CAVIN1 ARHGAP29 CCL2
FUS-GFP WT FUS-GFP P525L
ACMS versus untreated: non-isogenic line pools
D
mut ALS-FUS
top 7 common Up genes
Gene expression
FUS R521C FUS R521L FUS R495X
CP TFPI OLFML2A SPARCL1 FBLN5 CD44 GFAP
E
WT Ctrl
top 12 common Up genes
Gene expression
Ctrl1 Ctrl2 Ctrl3
GCNT3 SLC47A2 GFAP TDO2 BEX5 DCN ITIH5 PNMA5 CFH HLA-DRA OMG AQP4
top 12 common Down genes
ROBO3 PAX7 NEUROD4 ST18 GSG1 ENSG00000280229 NHLH1 NEUROG1 KCNQ1DN FZD10-AS1 NEUROG3 PMEL
Untreated ACMS

Figure S13: Top DEGs in all cell lines. Refers to volcano plots in Figure 4D, S8E-K. Bar graphs showing expression levels of the top upregulated and downregulated DEGs as shown in the volcano plots by means of top fold changes (FC) and adjusted p-values ≤ 0.05 derived from the DESeq2 differential expression adjusted for multiple testing (see Material and Methods). Data are represented as mean of the normalized short read counts mapped against the respective gene locus ± SEM (error bars). (**A**) Top 15 upregulated (left) and downregulated (right) genes in FUS-GFP P525L MNs through ACMS (orange) compared to untreated (blue). Refers to Figure 4D bottom. (**B**) Top 15 upregulated (left) and downregulated (right) genes in FUS-GFP WT MNs through ACMS (orange) compared to untreated (blue). The strongly upregulated GFAP was re-scaled in a separated inlet. Refers to Figure 4D top. (**C**) Top 15 upregulated (left) and downregulated (right) genes in the isogenic pair of FUS-GFP P525L (red) versus FUS-GFP WT (purple) MNs (both untreated). Refers to Figure S8E. (**D**) Common top seven upregulated genes in the line pool of non-isogenic mutant FUS MNs (each line ACMS, orange, versus untreated, blue, respectively). Refers to Figure S8I-K. The term "common" refers to the overlap of all three lines in the Venn diagrams of DEGs in Figure S11A, B. Due to the relatively low number of common downregulated DEGs (399, Figure S11B), no top downregulated genes are shown here. (**E**) Common top 12 upregulated (left) and downregulated (right) genes in the line pool of non-isogenic WT Ctrl MNs (each line ACMS, orange, versus untreated, blue, respectively). Refers to Figure S8F-H. The term "common" refers to the overlap of all three lines in the Venn diagrams of DEGs in Figure S12A, B.

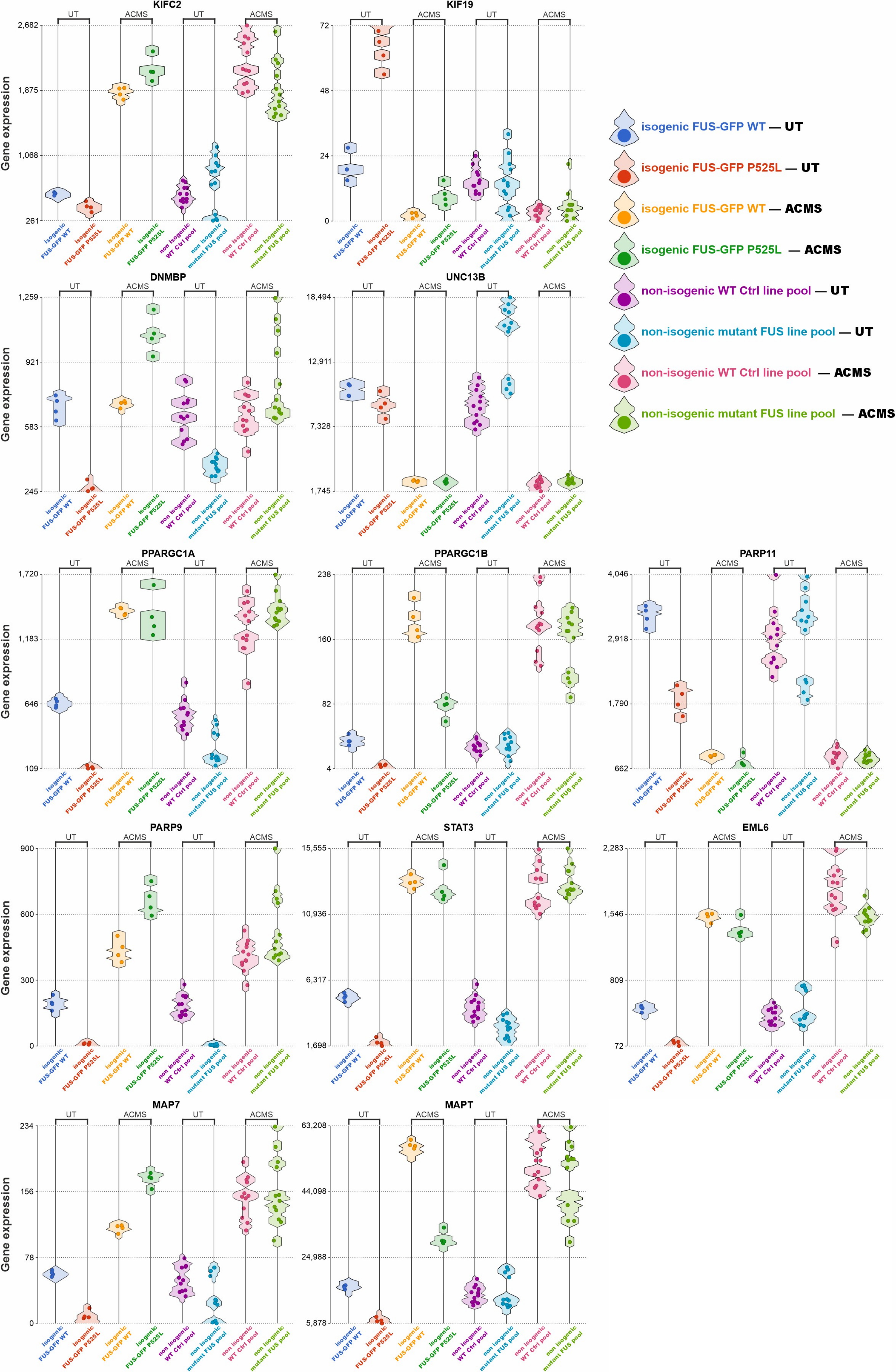
KIFC2
KIF19
DNMBP
UNC13B
PPARGC1A
PPARGC1B
PARP11
PARP9
STAT3
EML6
MAP7
MAPT
UT
ACMS
Gene expression
2,682
1,875
1,068
261
72
48
24
0
1,259
921
583
245
18,494
12,911
7,328
1,745
1,720
1,183
646
109
238
160
82
4
4,046
2,918
1,790
662
900
600
300
0
15,555
10,936
6,317
1,698
2,283
1,546
809
72
234
156
78
0
63,208
44,098
24,988
5,878
isogenic FUS-GFP WT
isogenic FUS-GFP P525L
non isogenic WT Ctrl pool
non isogenic mutant FUS pool
isogenic FUS-GFP WT — UT
isogenic FUS-GFP P525L — UT
isogenic FUS-GFP WT — ACMS
isogenic FUS-GFP P525L — ACMS
non-isogenic WT Ctrl line pool — UT
non-isogenic mutant FUS line pool — UT
non-isogenic WT Ctrl line pool — ACMS
non-isogenic mutant FUS line pool — ACMS

Figure S14: Scatter plot analysis of selected candidates from Table S4 common across all datasets. Violin plots illustrating commonly altered gene expression levels through ACMS in MNs across all mutant FUS and WT control lines (eight cell lines in total, Table 1). The x-axis shows the samples (isogenic and non-isogenic), and the Y-axis represents the expression level of genes (i.e., normalized short read counts mapped against the respective gene locus) depending on genotype and treatment (ACMS versus UT). Each dot within a violin contour represents one biological replica of the respective line. Thus, each violin of a single isogenic line contains four dots (Material and Methods) whereas violins for non-isogenic MNs contain 12 dots due to three lines pooled (Table 1). Selection criteria for common alteration in gene expression (ACMS versus untreated in respective line) of these top genes were as follows: common fold change (FC) ≥ 2 ($\log_2$ FC ≥ 1) for upregulated or FC ≤ 0.5 ($\log_2$ FC ≤ -1) for downregulated DEGs and p ≤ 0.01 in either (i) all eight lines (Table 1) or specifically in either (ii) all four lines of the mutant FUS pool or specifically (iii) in all four lines of the WT Ctrl pool (including the isogenic lines, Table 1). By these criteria, ACMS caused a significant upregulation of KIFC2, PPARGC1A, PPARGC1B, PARP9, STAT3, EML6, MAP7, and MAPT (Tau) in all eight lines, whereas KIF19, UNC13B, and PARP11 were significantly downregulated in all eight lines. Conversely, DNMBP was specifically upregulated in all four mutant FUS lines while remaining unaltered through ACMS in the entire WT Ctrl line pool.

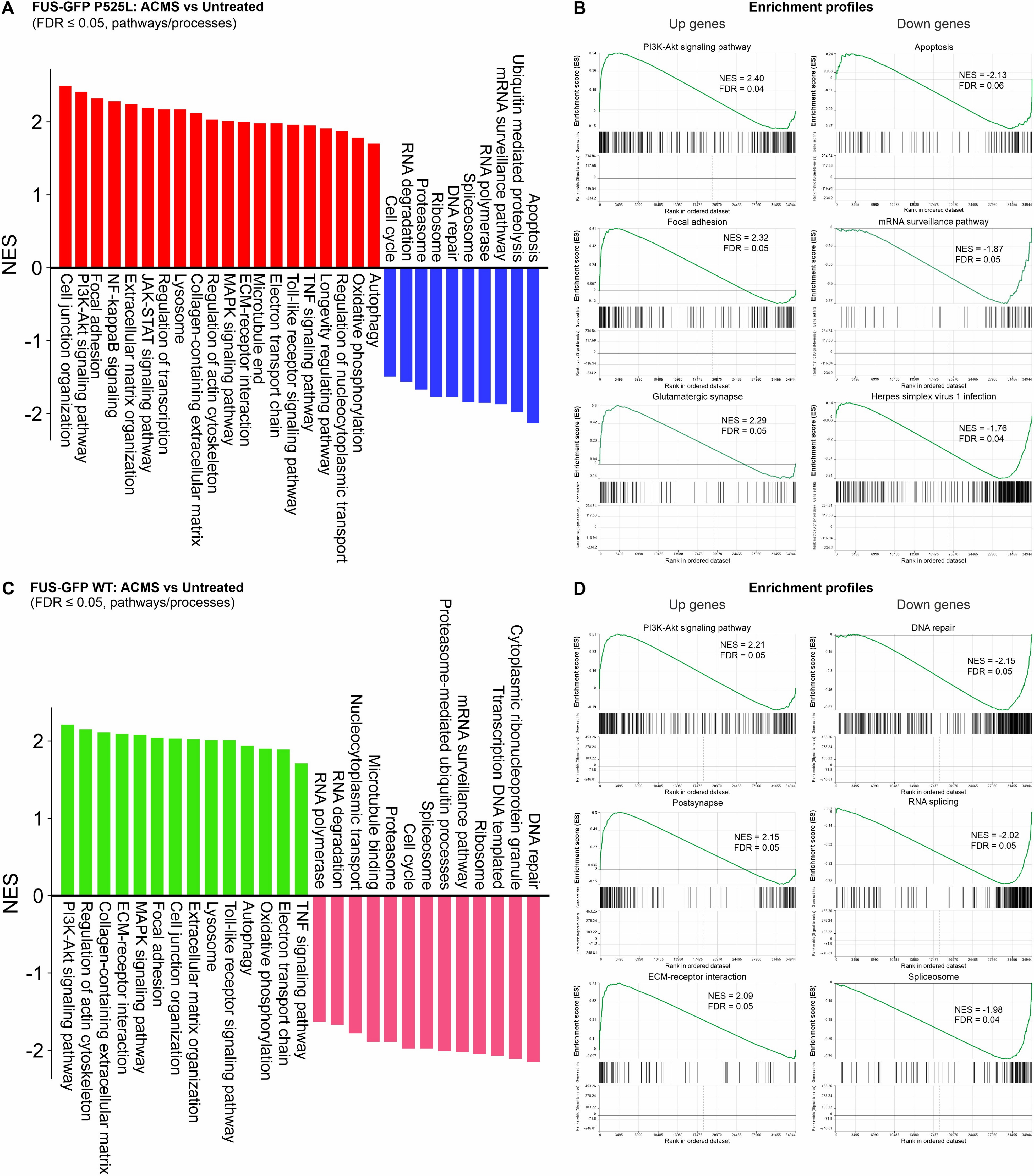
A
FUS-GFP P525L: ACMS vs Untreated
(FDR ≤ 0.05, pathways/processes)
NES
Cell junction organization
PI3K-Akt signaling pathway
Focal adhesion
NF-kappaB signaling
Extracellular matrix organization
JAK-STAT signaling pathway
Regulation of transcription
Lysosome
Collagen-containing extracellular matrix
Regulation of actin cytoskeleton
MAPK signaling pathway
ECM-receptor interaction
Microtubule end
Electron transport chain
Toll-like receptor signaling pathway
TNF signaling pathway
Longevity regulating pathway
Regulation of nucleocytoplasmic transport
Oxidative phosphorylation
Autophagy
Cell cycle
RNA degradation
Proteasome
Ribosome
DNA repair
Spliceosome
RNA polymerase
mRNA surveillance pathway
Ubiquitin mediated proteolysis
Apoptosis
B
Enrichment profiles
Up genes
Down genes
PI3K-Akt signaling pathway
NES = 2.40
FDR = 0.04
Apoptosis
NES = -2.13
FDR = 0.06
Focal adhesion
NES = 2.32
FDR = 0.05
mRNA surveillance pathway
NES = -1.87
FDR = 0.05
Glutamatergic synapse
NES = 2.29
FDR = 0.05
Herpes simplex virus 1 infection
NES = -1.76
FDR = 0.04
Enrichment score (ES)
Rank in ordered dataset
C
FUS-GFP WT: ACMS vs Untreated
(FDR ≤ 0.05, pathways/processes)
NES
PI3K-Akt signaling pathway
Regulation of actin cytoskeleton
Collagen-containing extracellular matrix
ECM-receptor interaction
MAPK signaling pathway
Focal adhesion
Cell junction organization
Extracellular matrix organization
Lysosome
Toll-like receptor signaling pathway
Autophagy
Oxidative phosphorylation
Electron transport chain
TNF signaling pathway
RNA polymerase
RNA degradation
Nucleocytoplasmic transport
Microtubule binding
Proteasome
Cell cycle
Spliceosome
Proteasome-mediated ubiquitin processes
mRNA surveillance pathway
Ribosome
Ttranscription DNA templated
Cytoplasmic ribonucleoprotein granule
DNA repair
D
Enrichment profiles
Up genes
Down genes
PI3K-Akt signaling pathway
NES = 2.21
FDR = 0.05
DNA repair
NES = -2.15
FDR = 0.05
Postsynapse
NES = 2.15
FDR = 0.05
RNA splicing
NES = -2.02
FDR = 0.05
ECM-receptor interaction
NES = 2.09
FDR = 0.05
Spliceosome
NES = -1.98
FDR = 0.04
Enrichment score (ES)
Rank in ordered dataset

Figure S15: Gene set enrichment analysis (GSEA) ACMS versus untreated in the isogenic pair of FUS-GFP P525L and FUS-GFP WT. (**A, C**) Bar plot depicting the NESs of the top most positively and negatively enriched gene sets, which present predominant pathways in the isogenic pair of (**A**) FUS-GFP P525L and (**C**) FUS-GFP WT MNs. (**B, D**) GSEAs were performed using normalized counts from the DESeq2 output ranked list in (**B**) FUS-GFP P525L or (**D**) FUS-GFP WT datasets (ACMS versus untreated for each line, respectively), $p \leq 0.05$, $FDR \leq 0.05$ was considered statistically significant. The top three most significantly enriched gene sets (based on NES values) are shown that either positively (left column) or negatively (right column) correlated with the gene counts. The y-axis represents enrichment scores (green curve) across the entire gene set for each individual gene member on the x-axis (vertical black bars) of that gene set.

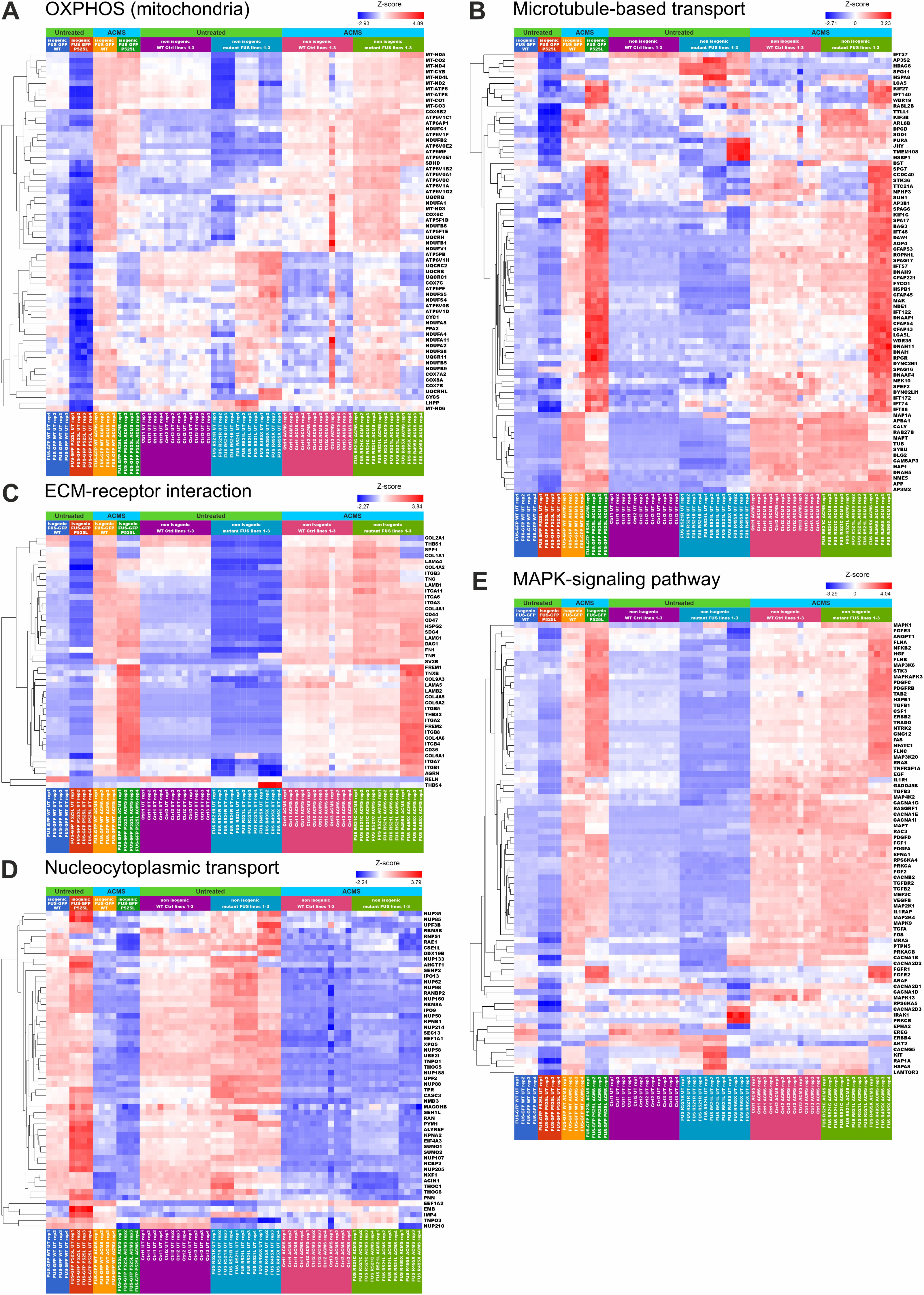
A
OXPHOS (mitochondria)
Z-score
-2.93
4.89
Untreated
ACMS
isogenic FUS-GFP WT
isogenic FUS-GFP P525L
non isogenic WT Ctrl lines 1-3
non isogenic mutant FUS lines 1-3
MT-ND5
MT-CO2
MT-ND4
MT-CYB
MT-ND4L
MT-ND2
MT-ATP6
MT-ATP8
MT-ND1
MT-CO1
MT-CO3
COX6B2
ATP6V1C1
ATP6AP1
NDUFC1
ATP6V1F
NDUFB2
ATP6V0E2
ATP5MF
ATP6V0E1
SDHD
ATP6V1B2
ATP6V0A1
ATP6V0C
ATP6V1A
ATP6V1G2
UQCRQ
NDUFA1
MT-ND3
COX6C
ATP5F1D
NDUFB6
ATP5F1E
UQCRH
NDUFB1
NDUFV1
ATP5PB
ATP6V1H
UQCRC2
UQCRB
UQCRC1
COX7C
ATP5PF
NDUFS5
NDUFS4
ATP6V0B
ATP6V1D
CYC1
NDUFA8
PPA2
NDUFA4
NDUFA11
NDUFA2
NDUFS8
UQCR11
NDUFB5
NDUFB9
COX7A2
COX8A
COX7B
UQCRHL
CYCS
LHPP
MT-ND6
B
Microtubule-based transport
Z-score
-2.71
0
3.23
C
ECM-receptor interaction
Z-score
-2.27
3.84
COL2A1
THBS1
SPP1
COL1A1
LAMA4
COL4A2
ITGB3
TNC
LAMB1
ITGA11
ITGA6
ITGA3
COL4A1
CD44
CD47
HSPG2
SDC4
LAMC1
DAG1
FN1
TNR
SV2B
FREM1
TNXB
COL9A3
LAMA5
LAMB2
COL4A5
COL6A2
ITGB5
THBS2
ITGA2
FREM2
ITGB8
COL4A6
ITGB4
CD36
COL6A1
ITGA7
ITGB1
AGRN
RELN
THBS4
D
Nucleocytoplasmic transport
Z-score
-2.24
3.79
E
MAPK-signaling pathway
Z-score
-3.29
0
4.04

Figure S16: GSEA leading edge gene analysis. Heat maps representing the relative expression change (ACMS versus untreated, based on Z-scores) of selected pathways (**A-E**) 'leading edge genes' (with top NES) which were obtained by GSEA analysis ($p \leq 0.05$, FDR ≤ 0.05) in all isogenic and non-isogenic samples (eight cell lines in total, four biological replicas per line, Table 1). Leading-edge analysis was performed on positively/negatively enriched gene sets that were significant in mutant FUS and WT Ctrl samples.

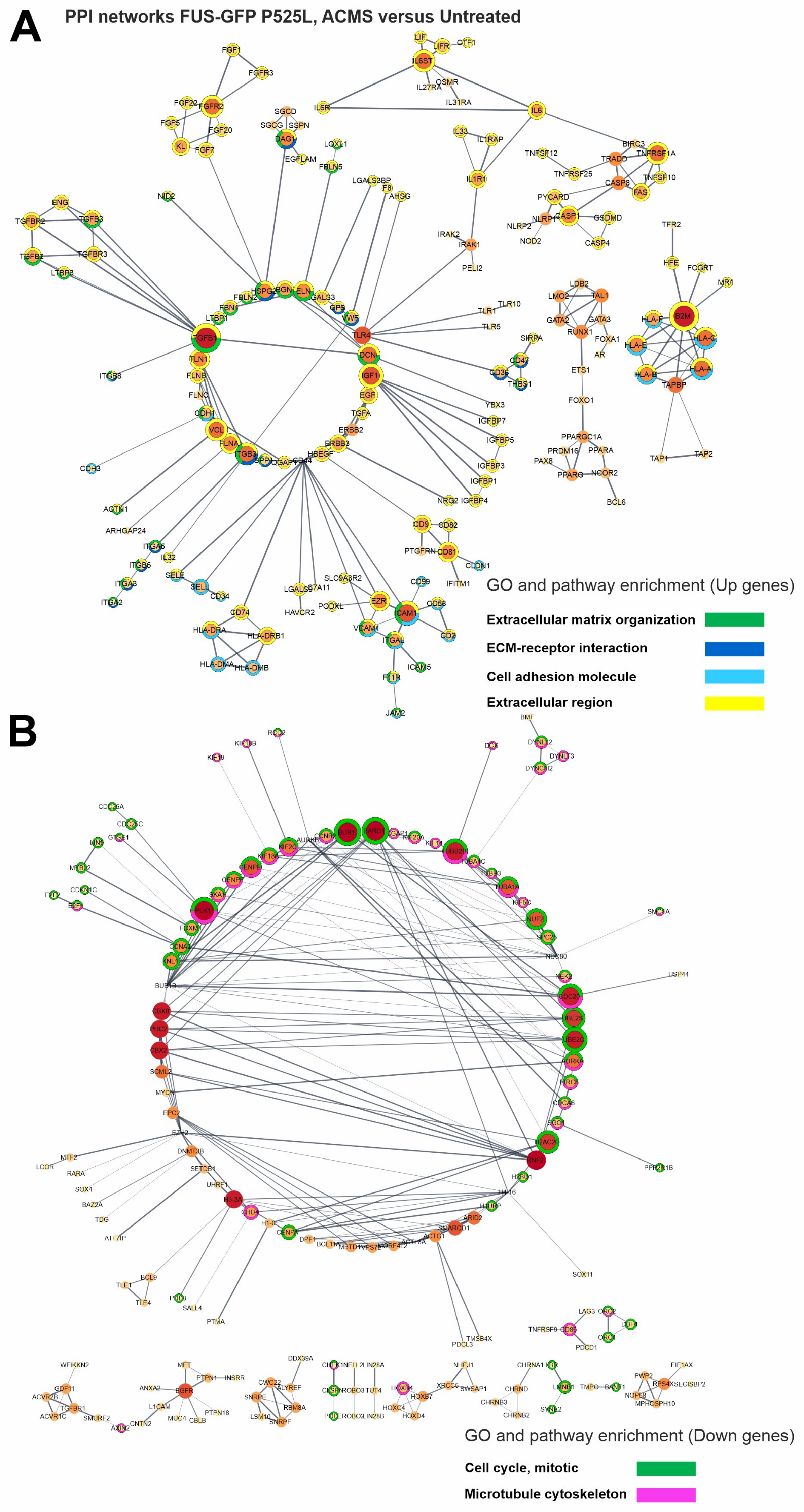

A
PPI networks FUS-GFP P525L, ACMS versus Untreated
GO and pathway enrichment (Up genes)
Extracellular matrix organization
ECM-receptor interaction
Cell adhesion molecule
Extracellular region
B
GO and pathway enrichment (Down genes)
Cell cycle, mitotic
Microtubule cytoskeleton

Figure S17: Refers to Figure 5F, G. STRING analysis of DEGs in isogenic FUS-GFP P525L MNs (ACMS versus untreated) involved in protein-protein interactions (PPI) between (**A**) upregulated and (**B**) downregulated specific genes showed distinct gene clusters. In the PPI network, each node represents a protein/DEG, and each edge represents an interaction. Node size indicates node degree value and edge color represents STRING enrichment score. Darker/thicker edges mean higher/stronger STRING score. The color of a node depends on its degree values (hubs); the darker the color, the higher the connectivity. Nodes depicted in darker color and with large shape represent their importance to the network. The rank and score of these key genes/clusters were identified with high degree of connectivity within the network (topological parameters). Corresponding top 15 up- and downregulated hub genes deduced from these PPI networks are shown in Figure 5F, G.

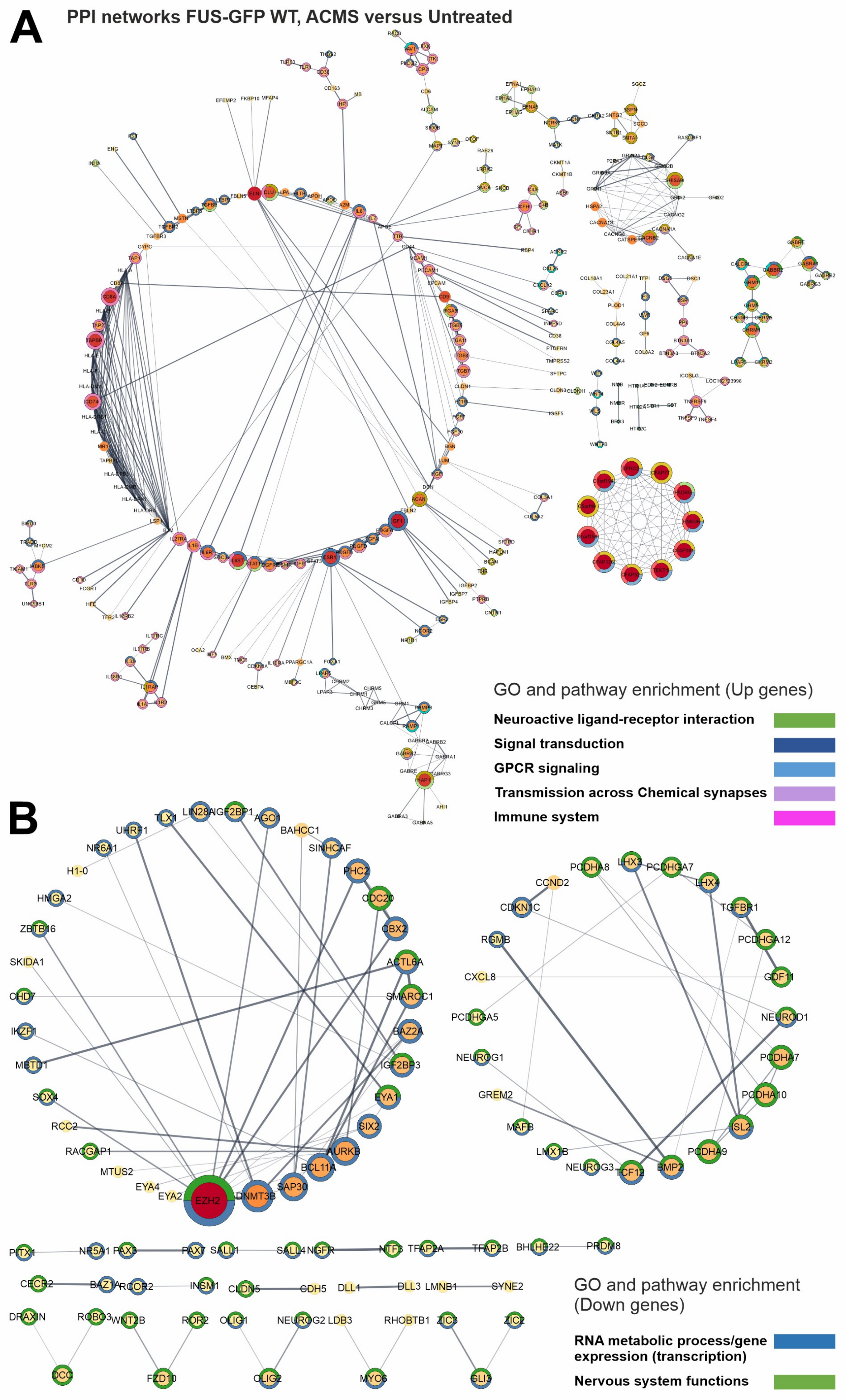
A
PPI networks FUS-GFP WT, ACMS versus Untreated
GO and pathway enrichment (Up genes)
Neuroactive ligand-receptor interaction
Signal transduction
GPCR signaling
Transmission across Chemical synapses
Immune system
B
LIN28A
IGF2BP1
TLX1
AGO1
UHRF1
BAHCC1
NR6A1
SINHCAF
H1-0
PHC2
HMGA2
CDC20
ZBTB16
CBX2
SKIDA1
ACTL6A
CHD7
SMARCC1
IKZF1
BAZ2A
MBTD1
IGF2BP3
SOX4
EYA1
RCC2
SIX2
RACGAP1
AURKB
MTUS2
BCL11A
EYA4
SAP30
EYA2
EZH2
DNMT3B
PCDHA8
LHX3
PCDHGA7
CCND2
LHX4
CDKN1C
TGFBR1
RGMB
PCDHGA12
CXCL8
GDF11
PCDHGA5
NEUROD1
NEUROG1
PCDHA7
GREM2
PCDHA10
MAFB
ISL2
LMX1B
PCDHA9
NEUROG3
TCF12
BMP2
PITX1
NR5A1
PAX3
PAX7
SALL1
SALL4
NGFR
NTF3
TFAP2A
TFAP2B
BHLHE22
PRDM8
CECR2
BAZ1A
RCOR2
INSM1
CLDN5
CDH5
DLL1
DLL3
LMNB1
SYNE2
DRAXIN
ROBO3
WNT2B
ROR2
OLIG1
NEUROG2
LDB3
RHOBTB1
ZIC3
ZIC2
DCC
FZD10
OLIG2
MYO6
GLI3
GO and pathway enrichment
(Down genes)
RNA metabolic process/gene expression (transcription)
Nervous system functions

Figure S18: Refers to Figure 6F, G. STRING analysis of DEGs in isogenic FUS-GFP WT MNs (ACMS versus untreated) involved in PPI between (**A**) upregulated and (**B**) downregulated specific genes showed distinct gene clusters. In the PPI network, each node represents a protein/DEG, and each edge represents an interaction. Node size indicates node degree value and edge color represents STRING enrichment score. Darker/thicker edges mean higher/stronger STRING score. The color of a node depends on its degree values (hubs); the darker the color, the higher the connectivity. Nodes depicted in darker color and with large shape represent their importance to the network. The rank and score of these key genes/clusters were identified with high degree of connectivity within the network (topological parameters). Corresponding top 15 up- and downregulated hub genes deduced from these PPI networks are shown in Figure 6F, G.

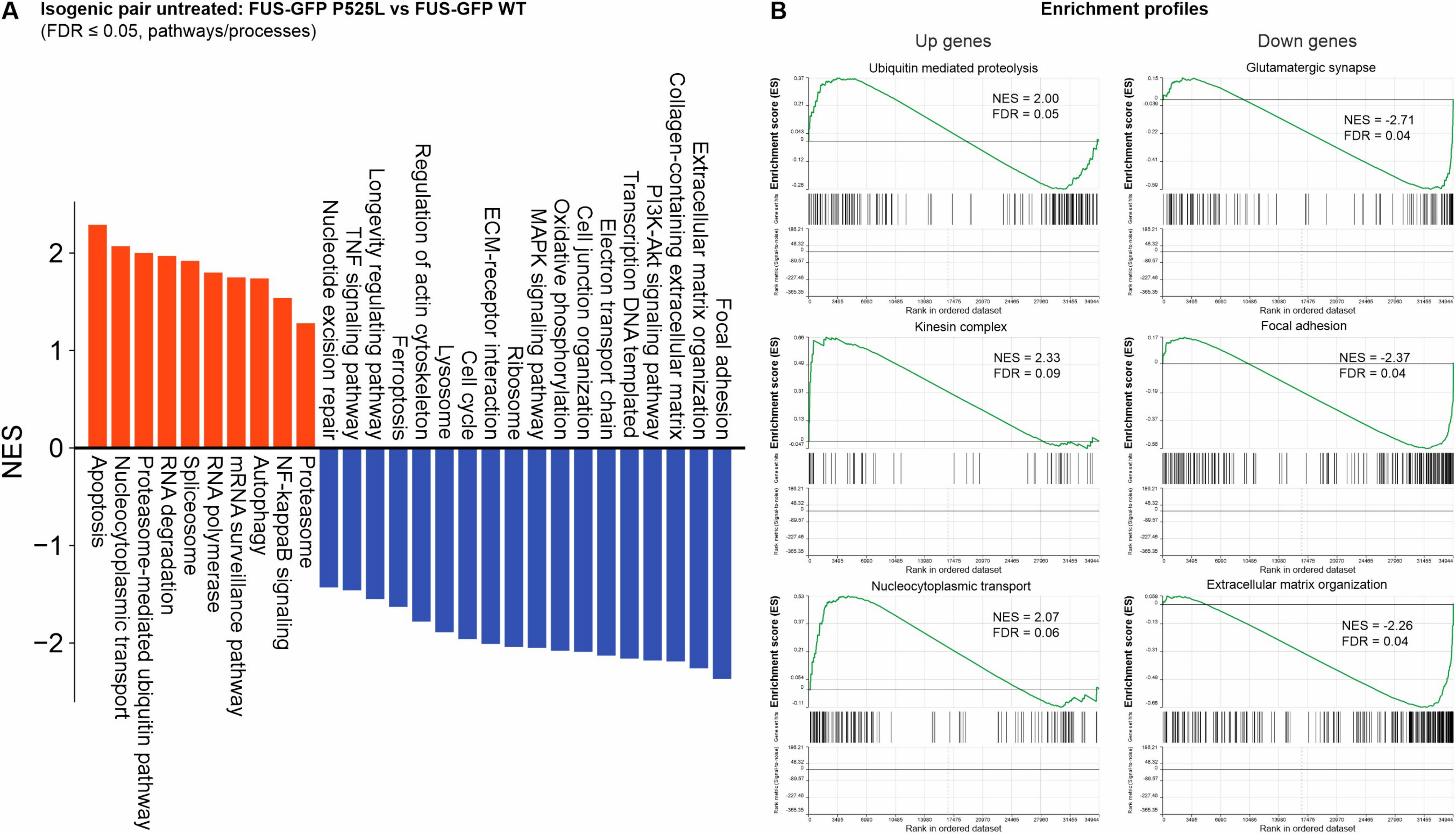
A
Isogenic pair untreated: FUS-GFP P525L vs FUS-GFP WT
(FDR ≤ 0.05, pathways/processes)
NES
Apoptosis
Nucleocytoplasmic transport
Proteasome-mediated ubiquitin pathway
RNA degradation
Spliceosome
RNA polymerase
mRNA surveillance pathway
Autophagy
NF-kappaB signaling
Proteasome
Nucleotide excision repair
TNF signaling pathway
Longevity regulating pathway
Ferroptosis
Regulation of actin cytoskeleton
Lysosome
Cell cycle
ECM-receptor interaction
Ribosome
MAPK signaling pathway
Oxidative phosphorylation
Cell junction organization
Electron transport chain
Transcription DNA templated
PI3K-Akt signaling pathway
Collagen-containing extracellular matrix
Extracellular matrix organization
Focal adhesion
B
Enrichment profiles
Up genes
Down genes
Ubiquitin mediated proteolysis
NES = 2.00
FDR = 0.05
Glutamatergic synapse
NES = -2.71
FDR = 0.04
Kinesin complex
NES = 2.33
FDR = 0.09
Focal adhesion
NES = -2.37
FDR = 0.04
Nucleocytoplasmic transport
NES = 2.07
FDR = 0.06
Extracellular matrix organization
NES = -2.26
FDR = 0.04
Enrichment score (ES)
Rank in ordered dataset

Figure S19: Gene set enrichment analysis (GSEA) in the untreated isogenic pair of FUS-GFP P525L versus FUS-GFP WT. (**A**) Bar plot depicting the NESs of the top most positively and negatively enriched gene sets, which present predominant pathways in the untreated isogenic pair of FUS-GFP P525L versus FUS-GFP WT MNs. (**B**) GSEAs were performed using normalized counts from the DESeq2 output ranked list in FUS-GFP P525L and FUS-GFP WT datasets (both untreated), $p \leq 0.05$, $FDR \leq 0.05$ was considered statistically significant. The top three most significantly enriched gene sets (based on NES values) are shown that either positively (left column) or negatively (right column) correlated with the gene counts. The y-axis represents enrichment scores (green curve) across the entire gene set for each individual gene member on the x-axis (vertical black bars) of that gene set.

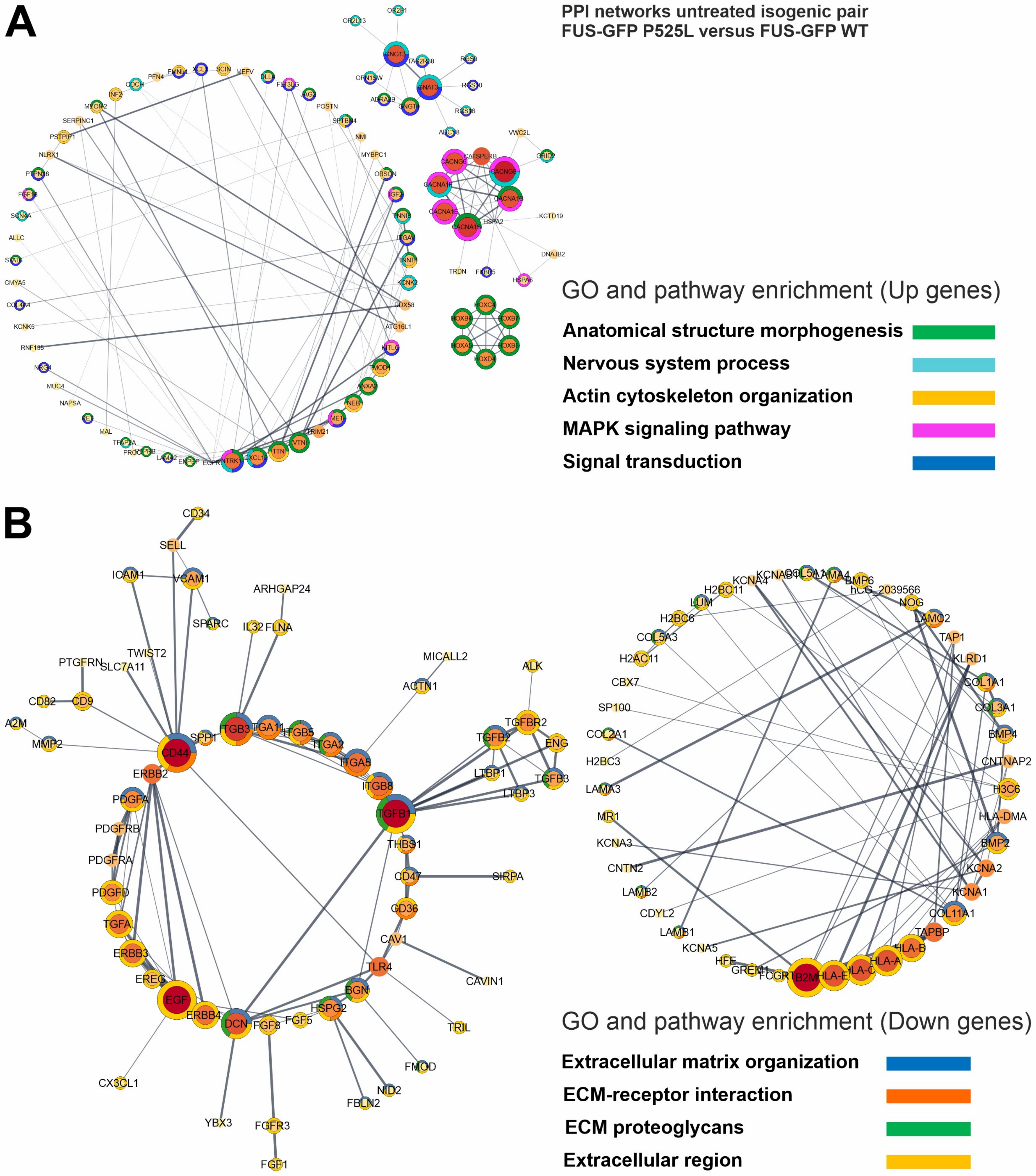
A
PPI networks untreated isogenic pair
FUS-GFP P525L versus FUS-GFP WT
GO and pathway enrichment (Up genes)
Anatomical structure morphogenesis
Nervous system process
Actin cytoskeleton organization
MAPK signaling pathway
Signal transduction
CACNG6
CATSPER8
CACNG8
CACNA1F
CACNA1G
CACNA1E
CACNA1S
HSPA2
KCTD19
DNAJB2
TRDN
HSPA6
VWC2L
GRID2
HOXC4
HOXB4
HOXB7
HOXA5
HOXB5
HOXD4
GNG13
GNAT3
ADRA2B
RGS9
RGS10
RGS16
SERPINC1
PSTPIP1
NLRX1
PTPN18
SCN4A
ALLC
STAT5
CMYA5
COL4A4
KCNK5
RNF185
NRG4
MUC4
NAPSA
RET
MAL
TFAP2A
LAMA2
ENPEP
EGFR
NTRK1
TTN
VTN
TRIM21
MET
NEB
ANXA2
TMOD1
KITLG
ATG16L1
DDX58
KCNK2
TNNT1
ITGAV
TNNI3
IGF2
OBSCN
MYBPC1
NMI
SPTBN4
POSTN
JAG2
FLT3LG
DLL4
MEFV
SCIN
XCL1
FMNL1
PFN4
COCH
INF2
MYOM2
B
CD34
SELL
ICAM1
VCAM1
SPARC
ARHGAP24
IL32
FLNA
TWIST2
PTGFRN
SLC7A11
CD82
CD9
A2M
MMP2
CD44
SPP1
ITGB3
ITGA11
ITGB5
ITGA2
ITGA5
ITGB8
MICALL2
ACTN1
ALK
TGFBR2
TGFB2
ENG
LTBP1
TGFB3
LTBP3
TGFB1
THBS1
CD47
SIRPA
CD36
CAV1
CAVIN1
TLR4
BGN
TRIL
HSPG2
FMOD
NID2
FBLN2
ERBB2
PDGFA
PDGFRB
PDGFRA
PDGFD
TGFA
ERBB3
EREG
EGF
ERBB4
DCN
FGF8
FGF5
CX3CL1
YBX3
FGFR3
FGF1
KCNA4
KCNAB1
COL5A1
LAMA4
BMP6
hCG_2039566
H2BC11
LUM
NOG
H2BC6
LAMC2
COL5A3
TAP1
H2AC11
KLRD1
CBX7
COL1A1
SP100
COL3A1
COL2A1
BMP4
H2BC3
CNTNAP2
LAMA3
H3C6
MR1
HLA-DMA
KCNA3
BMP2
CNTN2
KCNA2
LAMB2
KCNA1
CDYL2
COL11A1
LAMB1
TAPBP
KCNA5
HLA-B
HFE
HLA-A
GREM1
HLA-C
FCGRT
B2M
HLA-E
GO and pathway enrichment (Down genes)
Extracellular matrix organization
ECM-receptor interaction
ECM proteoglycans
Extracellular region

Figure S20: Refers to Figure S10F, G. STRING analysis of DEGs in the untreated isogenic pair of FUS-GFP P525L versus FUS-GFP WT MNs involved in PPI between (**A**) upregulated and (**B**) downregulated specific genes showed distinct gene clusters. In the PPI network, each node represents a protein/DEG, and each edge represents an interaction. Node size indicates node degree value and edge color represents STRING enrichment score. Darker/thicker edges mean higher/stronger STRING score. The color of a node depends on its degree values (hubs); the darker the color, the higher the connectivity. Nodes depicted in darker color and with large shape represent their importance to the network. The rank and score of these key genes/clusters were identified with high degree of connectivity within the network (topological parameters). Corresponding top 15 up- and downregulated hub genes deduced from these PPI networks are shown in Figure S10F, G.

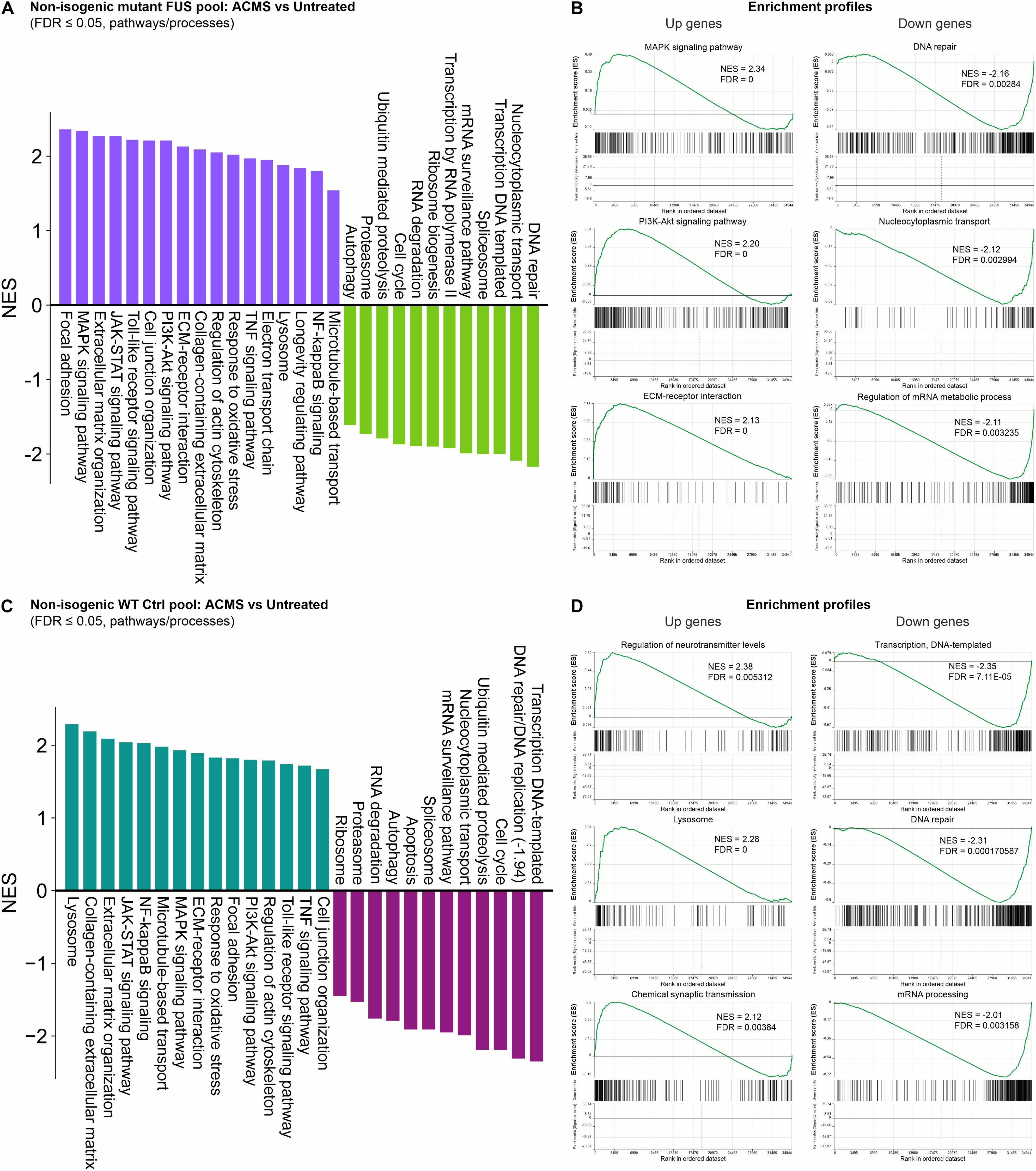

A
Non-isogenic mutant FUS pool: ACMS vs Untreated
(FDR ≤ 0.05, pathways/processes)
NES
2
1
0
-1
-2
Focal adhesion
MAPK signaling pathway
Extracellular matrix organization
JAK-STAT signaling pathway
Toll-like receptor signaling pathway
Cell junction organization
PI3K-Akt signaling pathway
ECM-receptor interaction
Collagen-containing extracellular matrix
Regulation of actin cytoskeleton
Response to oxidative stress
TNF signaling pathway
Electron transport chain
Lysosome
Longevity regulating pathway
NF-kappaB signaling
Microtubule-based transport
Autophagy
Proteasome
Ubiquitin mediated proteolysis
Cell cycle
RNA degradation
Ribosome biogenesis
Transcription by RNA polymerase II
mRNA surveillance pathway
Spliceosome
Transcription DNA templated
Nucleocytoplasmic transport
DNA repair
B
Enrichment profiles
Up genes
Down genes
MAPK signaling pathway
NES = 2.34
FDR = 0
DNA repair
NES = -2.16
FDR = 0.00284
PI3K-Akt signaling pathway
NES = 2.20
FDR = 0
Nucleocytoplasmic transport
NES = -2.12
FDR = 0.002994
ECM-receptor interaction
NES = 2.13
FDR = 0
Regulation of mRNA metabolic process
NES = -2.11
FDR = 0.003235
Enrichment score (ES)
Rank in ordered dataset
C
Non-isogenic WT Ctrl pool: ACMS vs Untreated
(FDR ≤ 0.05, pathways/processes)
NES
2
1
0
-1
-2
Lysosome
Collagen-containing extracellular matrix
Extracellular matrix organization
JAK-STAT signaling pathway
NF-kappaB signaling
Microtubule-based transport
MAPK signaling pathway
ECM-receptor interaction
Response to oxidative stress
Focal adhesion
PI3K-Akt signaling pathway
Regulation of actin cytoskeleton
Toll-like receptor signaling pathway
TNF signaling pathway
Cell junction organization
Ribosome
Proteasome
RNA degradation
Autophagy
Apoptosis
Spliceosome
mRNA surveillance pathway
Nucleocytoplasmic transport
Ubiquitin mediated proteolysis
Cell cycle
DNA repair/DNA replication (-1.94)
Transcription DNA-templated
D
Enrichment profiles
Up genes
Down genes
Regulation of neurotransmitter levels
NES = 2.38
FDR = 0.005312
Transcription, DNA-templated
NES = -2.35
FDR = 7.11E-05
Lysosome
NES = 2.28
FDR = 0
DNA repair
NES = -2.31
FDR = 0.000170587
Chemical synaptic transmission
NES = 2.12
FDR = 0.00384
mRNA processing
NES = -2.01
FDR = 0.003158
Enrichment score (ES)
Rank in ordered dataset

Figure S21: Gene set enrichment analysis (GSEA) ACMS versus untreated in the non-isogenic pools of mutant FUS and WT Ctrl lines. (**A, C**) Bar plot depicting the NESs of the top most positively and negatively enriched gene sets, which present predominant pathways in (**A**) the pool of non-isogenic mutant FUS lines or (**C**) of non-isogenic WT Ctrl lines (each ACMS versus untreated, respectively). (**B, D**) GSEAs were performed using normalized counts from the DESeq2 output ranked list in (**B**) the pool of non-isogenic mutant FUS lines or (**D**) of non-isogenic WT Ctrl lines (each ACMS versus untreated, respectively), $p \leq 0.05$, $FDR \leq 0.05$ was considered statistically significant. The top three most significantly enriched gene sets (based on NES values) are shown that either positively (left column) or negatively (right column) correlated with the gene counts. The y-axis represents enrichment scores (green curve) across the entire gene set for each individual gene member on the x-axis (vertical black bars) of that gene set.

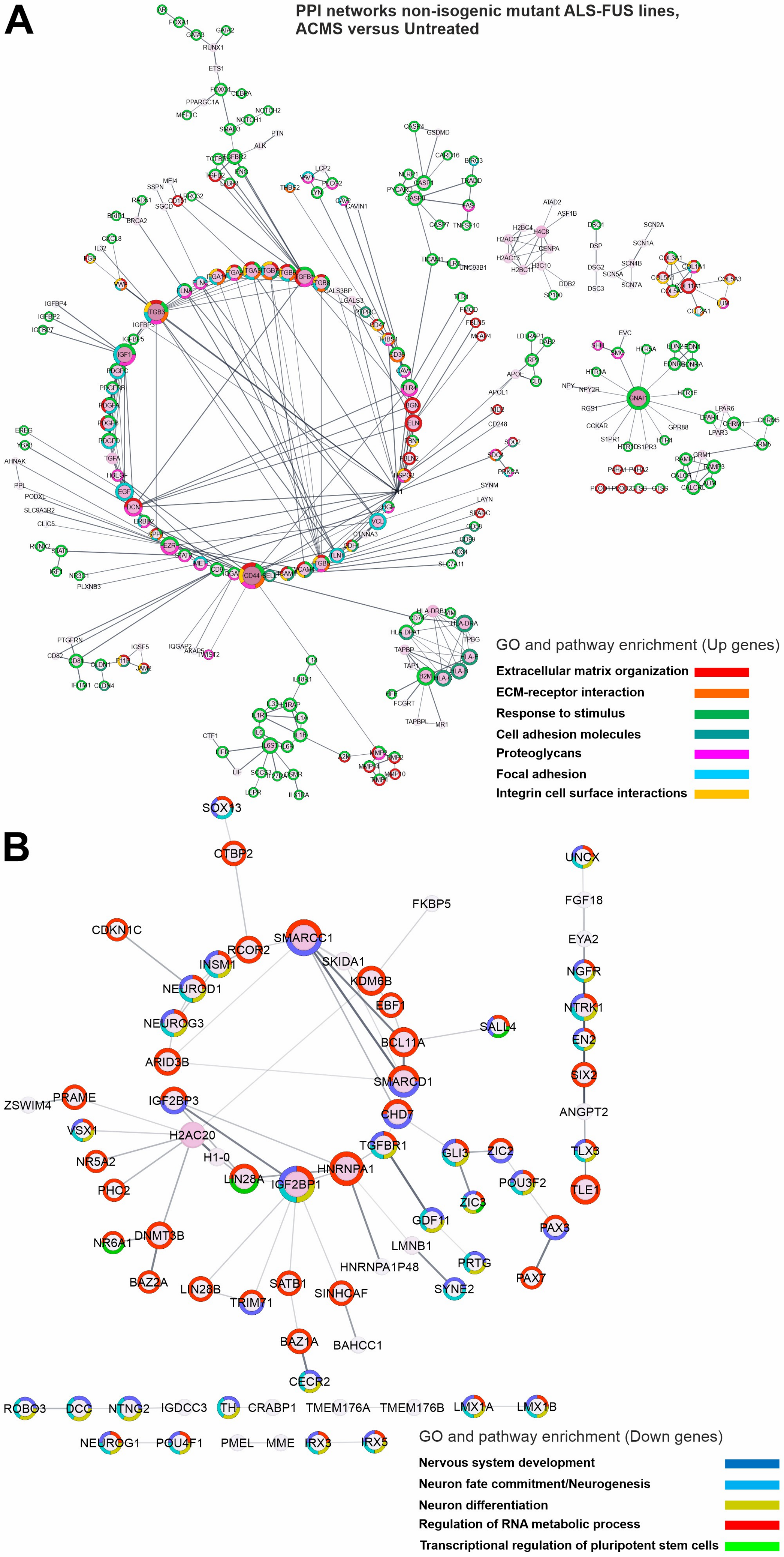
A
PPI networks non-isogenic mutant ALS-FUS lines, ACMS versus Untreated
GO and pathway enrichment (Up genes)
Extracellular matrix organization
ECM-receptor interaction
Response to stimulus
Cell adhesion molecules
Proteoglycans
Focal adhesion
Integrin cell surface interactions
B
SOX13
CTBP2
CDKN1C
RCOR2
SMARCC1
FKBP5
SKIDA1
INSM1
NEUROD1
KDM6B
EBF1
NEUROG3
BCL11A
SALL4
ARID3B
SMARCD1
PRAME
ZSWIM4
IGF2BP3
CHD7
VSX1
H2AC20
TGFBR1
GLI3
ZIC2
NR5A2
H1-0
HNRNPA1
LIN28A
IGF2BP1
POU3F2
PHC2
ZIC3
GDF11
NR6A1
DNMT3B
LMNB1
PRTG
HNRNPA1P48
BAZ2A
LIN28B
SATB1
SINHCAF
SYNE2
TRIM71
BAZ1A
BAHCC1
CECR2
PAX3
PAX7
UNCX
FGF18
EYA2
NGFR
NTRK1
EN2
SIX2
ANGPT2
TLX3
TLE1
ROBO3
DCC
NTNG2
IGDCC3
TH
CRABP1
TMEM176A
TMEM176B
LMX1A
LMX1B
NEUROG1
POU4F1
PMEL
MME
IRX3
IRX5
GO and pathway enrichment (Down genes)
Nervous system development
Neuron fate commitment/Neurogenesis
Neuron differentiation
Regulation of RNA metabolic process
Transcriptional regulation of pluripotent stem cells

Figure S22: Refers to Figure S11G, H. STRING analysis of common DEGs in the non-isogenic line pool of mutant FUS MNs (each ACMS versus untreated, respectively) involved in PPI between (**A**) upregulated and (**B**) downregulated specific genes showed distinct gene clusters. In the PPI network, each node represents a protein/DEG, and each edge represents an interaction. Node size indicates node degree value and edge color represents STRING enrichment score. Darker/thicker edges mean higher/stronger STRING score. The color of a node depends on its degree values (hubs); the darker the color, the higher the connectivity. Nodes depicted in darker color and with large shape represent their importance to the network. The rank and score of these key genes/clusters were identified with high degree of connectivity within the network (topological parameters). Corresponding top 15 up- and downregulated hub genes deduced from these PPI networks are shown in Figure S11G, H.

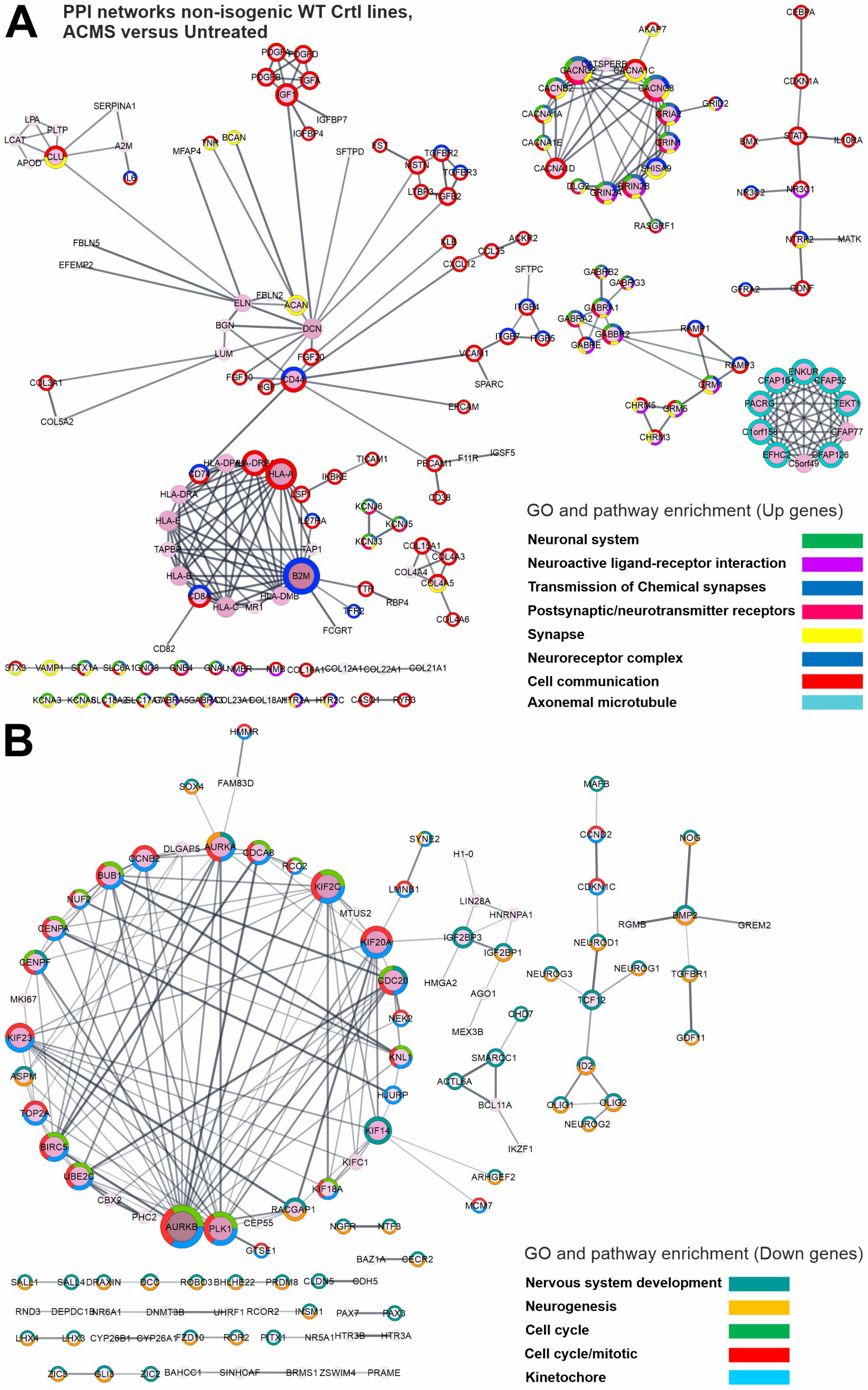
A
PPI networks non-isogenic WT Crtl lines, ACMS versus Untreated
GO and pathway enrichment (Up genes)
Neuronal system
Neuroactive ligand-receptor interaction
Transmission of Chemical synapses
Postsynaptic/neurotransmitter receptors
Synapse
Neuroreceptor complex
Cell communication
Axonemal microtubule
B
GO and pathway enrichment (Down genes)
Nervous system development
Neurogenesis
Cell cycle
Cell cycle/mitotic
Kinetochore

Figure S23. Refers to Figure S12G, H. STRING analysis of common DEGs in the non-isogenic line pool of WT Ctrl MNs (each ACMS versus untreated, respectively) involved in PPI between (**A**) upregulated and (**B**) downregulated specific genes showed distinct gene clusters. In the PPI network, each node represents a protein/DEG, and each edge represents an interaction. Node size indicates node degree value and edge color represents STRING enrichment score. Darker/thicker edges mean higher/stronger STRING score. The color of a node depends on its degree values (hubs); the darker the color, the higher the connectivity. Nodes depicted in darker color and with large shape represent their importance to the network. The rank and score of these key genes/clusters were identified with high degree of connectivity within the network (topological parameters). Corresponding top 15 up- and downregulated hub genes deduced from these PPI networks are shown in Figure S12G, H.

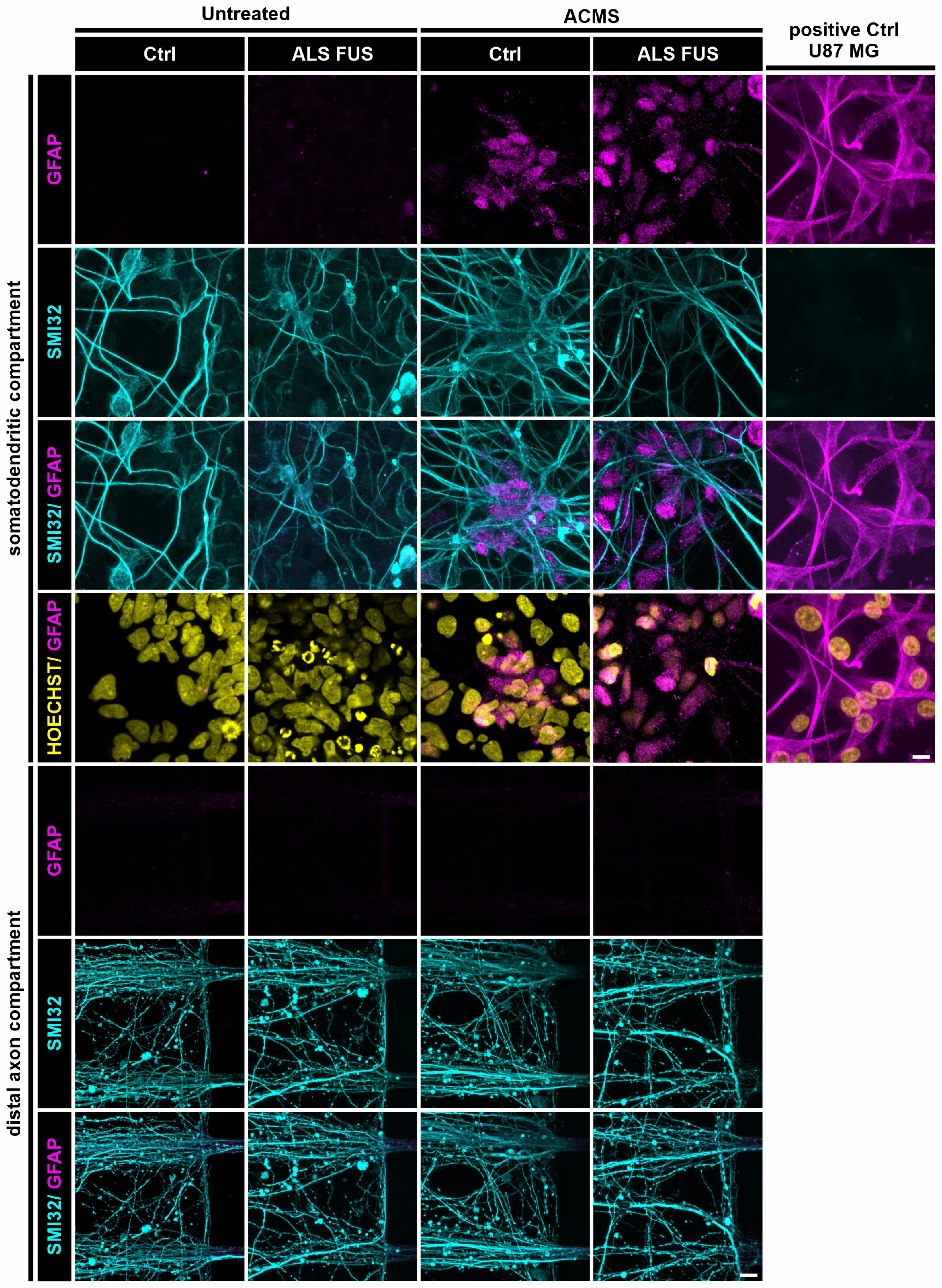
Untreated
ACMS
positive Ctrl
U87 MG
Ctrl
ALS FUS
Ctrl
ALS FUS
somatodendritic compartment
GFAP
SMI32
SMI32/ GFAP
HOECHST/ GFAP
distal axon compartment
GFAP
SMI32
SMI32/ GFAP

Figure S24. Induction of *GFAP* gene expression through ACMS in iPSC-derived spinal MNs leads apparently to no formation of cytoskeletal GFAP-positive filaments and to no cell fate change. Isogenic FUS-GFP WT (Ctrl) and P525L (ALS FUS) (Table 1) MNs matured in MFCs (Figure 1 H, I) either untreated or ACMS-treated were fixed at 48 DIV (Figure 1J) and co-immuno-stained for the glial cell marker GFAP (in magenta) and the MN marker SMI32 (neurofilament heavy chain, NFH, in cyan). Nuclei were revealed with Hoechst 33342 (in yellow). The human gliablastoma cell line U87 MG served as a positive control in which the typical cytoskeletal filaments were revealed, thereby validating the specificity of the GFAP antibody. No GFAP was detected in either untreated cell line, neither in the somatodendritic (top galleries), nor the distal axon compartment (bottom galleries), whereas correct motoneuronal differentiation was confirmed in both compartments with SMI32 antibody. Conversely, ACMS led to the occurrence of GFAP-positive cytosolic puncta in the somatodentritic compartment in both Ctrl and ALS FUS cells (top galleries) which were structurally were distinct from typical cytoskeletal filaments in the U87 MG positive control, thereby suggesting that ACMS-induced GFAP-expression was not leading to the formation of the typical filamentous structures. These GFAP-positive puncta were outside Hoechst-positive nuclei within the same confocal plane, thereby confirming their cytosolic/perinuclear location. Conversely, in the distal axon compartment (bottom galleries) GFAP remained undetectable after ACMS and SMI32-positive neurofilaments unaltered in both compartments, thereby arguing against a change in cell fate through induction of GFAP. N = 10 confocal images per condition, scale bars = 10 μm.

2. Tables S1-7

Table S1: full DEG results from all eight lines (iPSC-derived MNs, Table 1), each ACMS versus untreated, as well as isogenic FUS-GFP P525L versus FUS-GFP WT, both untreated.

Table S2: unfiltered ranked DEG lists of all eight lines (iPSC-derived MNs, Table 1), each ACMS versus untreated, as well as isogenic FUS-GFP P525L versus FUS-GFP WT, both untreated.

Table S3: GO and Reactome term analyses of top 500 absolute gene loadings on PC1 of the PCA plot in Figure 4A, separated by positive and negative loadings.

Table S4: short list of key DEGs common in all lines, separated by up- and downregulated candidates.

Table S5: GO and Pathway term analyses results of the DEGs from isogenic FUS-GFP P525L and FUS-GFP WT (Table 1) MNs (each ACMS versus untreated, Fig. 5B-D, 6B-D) and FUS-GFP P525L versus FUS-GFP WT (both untreated, Fig. S10B-D) as well as of the common DEGs (Venn

diagrams, Fig. S11A, B, S12A, B) from the non-isogenic pools of three mutant ALS-FUS (Table 1, Fig. S11C-E) and three Ctrl lines (Table 1, Fig. S12C-E), each ACMS versus untreated.

Table S6: GSEA analysis results for normalized gene counts from isogenic FUS-GFP P525L and FUS-GFP WT (Table 1) MNs (each ACMS versus untreated, Fig. 5E, 6E, S14) and FUS-GFP P525L versus FUS-GFP WT (both untreated, Fig. S10E, S19) as well as from pooled datasets of the three non-isogenic mutant ALS-FUS (Table 1, Fig. S11F, S21A, B) and three non-isogenic Ctrl lines (Table 1, Fig. S12F, S21C, D), each ACMS versus untreated.

Table S7: PPI analysis results of the DEGs from isogenic FUS-GFP P525L and FUS-GFP WT (Table 1) MNs (each ACMS versus untreated, Fig. S17, S18) and FUS-GFP P525L versus FUS-GFP WT (both untreated, Fig. S20) as well as of the common DEGs (Venn diagrams, Fig. S11A, B, S12A, B) from the non-isogenic pools of three mutant ALS-FUS (Table 1, Fig. S22) and three Ctrl lines (Table 1, Fig. S23), each ACMS versus untreated.

3. Movies S1-7

Common remarks about Movies S1-4 and S7:

Axonal organelle motility at the distal MFC channel ends as illustrated in Figure 1I acquired at 48 DIV (Figure 1J) with lyso- and mitotracker at 3.3 fps over 2 min at 100x magnification oil immersion, NA 1.46. Refer to Material and Methods for details. Shown are representative examples of the more motile lysosomes.

Common remarks about Movies S5 and S6:

Recruitment-dissociation of FUS-GFP to nuclear DDSs (laser cuts) immediately after laser irradiation acquired at 1 fps over 15 min at 100x magnification oil immersion, NA 1.45. Fluorescence emission of eGFP is shown in the LUT "Green Fire Blue" of the FIJI software, i.e. low eGFP intensities are shown in blue and high intensities in green shades, no nuclear HOECHST staining or other fluorescent dyes were used as detailed in Material and Methods.

Movie S1: Refers to the corresponding maximum intensity projections in Figure 2A, S3A and documents the rescue of axonal organelle motility in mutant FUS MNs depending on ACMS frequency with an optimum at 25 Hz at otherwise 10 mT and perpendicular field orientation as illustrated in Figure 1A.

Movie S2: Refers to the corresponding maximum intensity projections in Figure S4A and documents the more efficient rescue of axonal organelle motility in mutant FUS MNs at perpendicular as

compared to parallel field orientation as illustrated in Figure 1A at otherwise optimal 25 Hz and 10 mT.

Movie S3: Refers to the corresponding maximum intensity projections in Figure S5A and documents the sustained rescue of axonal organelle motility in mutant FUS MNs over at least eight days after the ACMS treatments at optimal 25 Hz and perpendicular field orientation at 10 mT as illustrated in Figure 1J.

Movie S4: Refers to the corresponding maximum intensity projections in Figure S6A and documents the critical threshold of field strength around 7 mT in the ACMS treatments (Figure 1J) at otherwise optimal 25 Hz and perpendicular field orientation to achieve the sustained rescue of axonal organelle motility in mutant FUS MNs.

Movie S5: Refers to the corresponding image gallery at 150 sec in Figure 3A and documents the rescue of nuclear DDR after laser irradiation, i.e. the recruitment-dissociation cycle of FUS-GFP at laser cuts, in mutant FUS MNs (FUS-GFP P525L, Table 1) depending on ACMS frequency (Figure 1J) with an optimum at 25 Hz at otherwise 10 mT and perpendicular field orientation as illustrated in Figure 1A.

Movie S6: Same as Movie 5 but for the ALS BAC HeLa cell model stably expressing FUS-GFP P525L (Table 1). Refers to the corresponding image gallery at 150 sec in Figure 3D.

Movie S7: Refers to Figure S7C and documents no ad hoc effect of ACMS during the movie recording after 10 min in mutant FUS MNs at optimal 25 Hz and 10 mT.
